# Evolution of SET-domain protein families in the unicellular and multicellular Ascomycota fungi

**DOI:** 10.1186/1471-2148-8-190

**Published:** 2008-07-01

**Authors:** Chendhore S Veerappan, Zoya Avramova, Etsuko N Moriyama

**Affiliations:** 1 School of Biological Sciences, University of Nebraska-Lincoln, Lincoln, NE, USA; 2 School of Biological Sciences and Center for Plant Science Innovation, University of Nebraska-Lincoln, Lincoln, NE, USA

## Abstract

**Background:**

The evolution of multicellularity is accompanied by the occurrence of differentiated tissues, of organismal developmental programs, and of mechanisms keeping the balance between proliferation and differentiation. Initially, the SET-domain proteins were associated exclusively with regulation of developmental genes in metazoa. However, finding of *SET-domain *genes in the unicellular yeasts *Saccharomyces cerevisiae *and *Schizosaccharomyces pombe *suggested that SET-domain proteins regulate a much broader variety of biological programs. Intuitively, it is expected that the numbers, types, and biochemical specificity of SET-domain proteins of multicellular *versus *unicellular forms would reflect the differences in their biology. However, comparisons across the unicellular and multicellular domains of life are complicated by the lack of knowledge of the ancestral *SET-domain *genes. Even within the crown group, different biological systems might use the epigenetic 'code' differently, adapting it to organism-specific needs. Simplifying the model, we undertook a systematic phylogenetic analysis of one monophyletic fungal group (Ascomycetes) containing unicellular yeasts, Saccharomycotina (hemiascomycetes), and a filamentous fungal group, Pezizomycotina (euascomycetes).

**Results:**

Systematic analysis of the *SET-domain *genes across an entire eukaryotic phylum has outlined clear distinctions in the *SET-domain *gene collections in the unicellular and in the multicellular (filamentous) relatives; diversification of *SET-domain *gene families has increased further with the expansion and elaboration of multicellularity in animal and plant systems. We found several ascomycota-specific *SET-domain *gene groups; each was unique to either Saccharomycotina or Pezizomycotina fungi. Our analysis revealed that the numbers and types of *SET-domain *genes in the Saccharomycotina did not reflect the habitats, pathogenicity, mechanisms of sexuality, or the ability to undergo morphogenic transformations. However, novel genes have appeared for functions associated with the transition to multicellularity. Descendents of most of the *SET*-*domain *gene families found in the filamentous fungi could be traced in the genomes of extant animals and plants, albeit as more complex structural forms.

**Conclusion:**

*SET-domain *genes found in the filamentous species but absent from the unicellular sister group reflect two alternative evolutionary events: deletion from the yeast genomes or appearance of novel structures in filamentous fungal groups. There were no Ascomycota-specific *SET-domain *gene families (*i.e*., absent from animal and plant genomes); however, plants and animals share *SET-domain *gene subfamilies that do not exist in the fungi. Phylogenetic and gene-structure analyses defined several animal and plant *SET*-*domain *genes as sister groups while those of fungal origin were basal to them. Plants and animals also share SET-domain subfamilies that do not exist in fungi.

## Background

The genes encoding proteins that contain SET-domain sequences (*SET-domain *genes) are ancient, existing in the Bacterial Domain of life [1], but have proliferated and evolved novel functions connected with the appearance of eukaryotes. Because SET-domain proteins modify chromatin by methylating specific lysines on the histone tails [2-4], it is not surprising that *SET-domain *genes are present in eukaryotes from the simple unicellular organisms to the multicellular animals and plants. The pattern and the complexity of epigenetic marks 'written' by the SET-domain proteins correlate with the increased requirements of multicellular organisms [5,6] including regulation of proliferation, ontogenesis, adhesion-mediated silencing, and disease [7-11].

The fungal division Ascomycota provides a unique model to trace the fate of the *SET-domain *genes in connection to multicellularity within one monophyletic group. It contains the unicellular Saccharomycotina (hemiascomycetes) and the multicellular filamentous fungi Pezizomycotina (or euascomycetes) as sister-groups. *Schizosaccharomyces pombe *(*S. pombe*), which belongs to the Taphrinomycotina (or archiascomycete), is an outgroup [12,13]. The fungi selected from the Saccharomycotina for this study include species existing as unicellular yeasts [*Saccharomyces cerevisiae *(*S. cerevisiae*), *Candida glabrata (C. glabrata), Debaryomyces hansenii *(*D. hansenii*), and *Yarrowia lipolytica *(*Y*.* lipolytica*)], as a permanently filamentous yeast, *Ashbya gossypii *(*A. gossypii*), or as an organism that changes morphology from the yeast to the filamentous forms in response to environmental cues, the dimorphic *Candida albicans *(*C. albicans*).

The Saccharomycotina has diverged from the filamentous fungi around 400 million years ago and at estimated >1 billion years ago from animals and plants [13-15]. Substantial events might have taken place during such periods of time; thereby, by comparing the *SET-domain *genes in unicellular and filamentous Ascomycetes, it might be possible to identify families that have evolved in connection with the appearance of the filamentous (multicellular) forms. *Neurospora crassa (N. crassa) *is a standard model for the Pezizomycotina (euascomycetes) group because it is a generalist species, less specialized in its biology than many pathogens, symbionts, and fungi of narrow habitat [16,17]. *N. crassa *is a bearer of the ancestral characteristics, allowing comparisons of *SET-domain *gene representation in the closely related pathogen *Magnaporthe grisea *(*M. grisea*), in the slightly more distant *Fusarium graminearum *(*F*. *graminearum*), and in the more distantly related *Aspergillus fumigatus *(*A. fumigatus*).* SET-domain *genes found in *N. crassa *and in the other filamentous fungi, but not in their unicellular relatives, could illustrate evolution of genes connected with transition to multicellular functions; unshared *SET-domain *genes between nonpathogenic and pathogenic relatives might be related to pathogenicity. Extended comparisons with SET-domain types and families present in the metazoan and plant genomes could outline evolutionary relationships between plant, animal, and fungal kingdoms. A few multicellular genomes, including an invertebrate (*Drosophila melanogaster*), a mammal (*Mus musculus*), and a plant (*Arabidopsis thaliana*), are provided as a reference and are not discussed in detail.

The main questions asked here were: *first*, whether the type and the number of *SET-domain *genes in species from the same phylum would correlate with their existence as unicellular or multicellular (filamentous) forms; *second*, whether the ability of some yeasts to acquire dimorphic forms, to exist as a permanently filamentous yeast, or to occupy a specific niche (*i.e*., to act in fermentation or as pathogens) would be reflected by the *SET-domain *gene collection within the genome; *third*, whether presence of certain *SET*-*domain *genes in a fungal genome would suggest occurrence of novel *SET-domain *genes or loss of existing *SET-domain *genes; and *fourth*, to examine the phylogenetic relationships between the *SET*-*domain *gene families of yeast, of higher filamentous fungi, of animals, and of plants to see if their evolution would parallel the transition steps from unicellular, to simple multicellular, and to more complex multicellular systems.

## Results

### Overall genome representation, distribution, and phylogenetic analysis of the fungal SET-domain proteins

**Figure 1:**
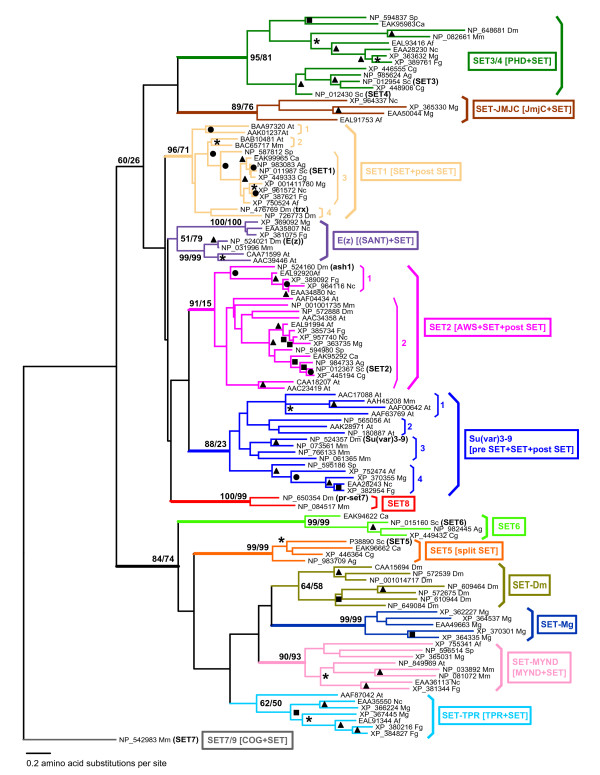
**Maximum likelihood phylogeny of 113 representative SET-domain sequences. **Bootstrap values for the major SET-domain families that are higher than 60% by either of the maximum likelihood (ML) or the maximum parsimony (MP) methods are shown at the node (the two % values are ML/MP). Internal branches supporting the major SET-domain families with higher than 80% ML bootstrap values are also indicated by thick lines. Within the major SET-domain protein groups, bootstrap values by the ML analysis greater than 60%, 70%, 80%, and 90% are indicated by stars (*), filled circles (●), filled squares (■), and filled triangles (▲), respectively. SET-domain protein subgroups discussed in the main text are indicated by numbers. Representative domain names are shown for the major SET-domain protein families (see Additional file 3 for the domain names). Species abbreviations are given in Additional file 1. For this ML phylogeny, the gamma shape parameter and the proportion of invariant sites were estimated to be 0.779 and 0, respectively.

**Figure 2:**
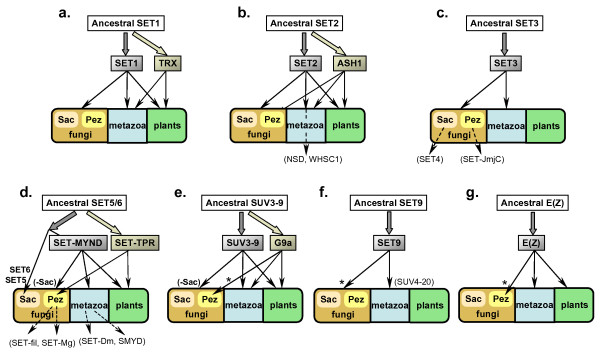
Distribution of SET-domain genes in the three kingdoms. Saccharomycotina and Pezizomycotina fungi are shown as "Sac" and "Pez", respectively, among the fungal kingdom. Species or group-specific duplication events of SET-domain genes are illustrated by dashed arrows. SET5/6 related filamentous fungi are shown as "SET-fil". Arrows marked with * indicate that such relationship is not significant and inconclusive. SET-MYND and SUV3-9 are found in *S. pombe *as well as Pezizomycotina fungi, but missing from Saccharomycotina fungi. This is indicated by the arrows marked with "(-Sac)".

Current phylogenetic studies of the kingdom Fungi define Ascomycota as a monophyletic group [12,13]. For an insight into the evolution of fungal *SET-domain *genes and their relationship with the genes of higher eukaryotes, we reconstructed phylogenetic trees using the highly conserved SET-domain region (~150 amino acids) [18]. First, we performed a series of similarity searches including profile hidden Markov models against fourteen genomes: eleven fungal (*Ascomycetes*), two animal (one mammalian and one invertebrate), and one plant (*Arabidopsis*) genomes (see Additional file 1). One hundred and eighty two SET-domain sequences were identified (see Additional file 2). Phylogenetic analyses were performed to identify the SET-domain protein families and subfamilies. Maximum likelihood phylogeny reconstructed from the selected 113 representative sequences is shown in Fig. 1. The phylogenetic clustering based on SET-domain sequences reflected presence/absence of group-specific architectural motifs (see Additional file 3). We also note that the SET-domain based phylogeny does not seem to support consistently any particular evolutionary relationships among the three kingdoms: fungi, animals, and plants (see also Fig. 2).

**Figure 3:**
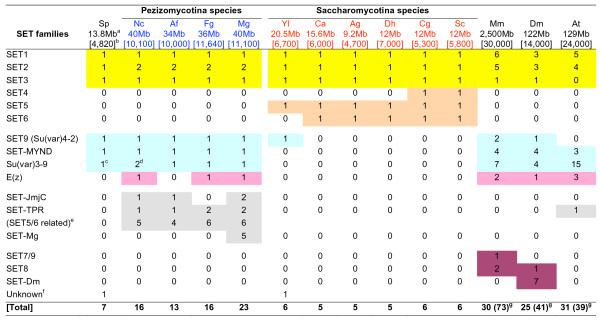
**Distribution of SET-domain families in fourteen genomes.** Shaded in yellow are genes found in all tested species; peach-colored genes were found only in the Saccharomycotina. Genes found in tested genomes except in the Saccharomycotina are shaded in turquoise, while those found only in multicellular species are shown in pink. Genes specific for the filamentous fungi are shown in grey. A related gene found in *Arabidopsis *is shaded in grey as well. Metazoa-specific genes are shaded in Bordeaux red. Footnotes: ^a ^genome size; ^b ^approximate numbers of predicted open reading frames; ^c ^the *Su(var)3-9 *gene in *S. pombe *is known as the *Clr4*; ^d ^one of the two copies is known as the *DIM-5 *gene in *N. crassa *and the second shows a weak similarity to G9a; ^e ^filamentous fungal genes belonging to the large SET5/6 family; ^f ^"unknown" genes have no significant support to cluster with any SET-domain family identified in this study; ^g ^numbers in parentheses indicate numbers of total SET-domain genes found in these animal/plant genomes [20,21].

The distribution of the SET-domain proteins across 14 genomes is summarized in Fig. 3. All 108 SET-domain sequences retrieved from the fungal genomes that belong to the group of the histone methyltransferases are included. SET-domain containing proteins from the RuBisCo, cytochrome C, and the recently discovered ribosomal protein lysine methyltransferases [19] are excluded. Among the SET-domain sequences of animal and plant origin, included are only those that are used as reference for the fungal proteins.

The sizes of the fourteen genomes and the total numbers of ORFs are not linearly correlated; the genomes of the filamentous fungi are approximately three times the size of the yeasts (except *Y. lipolytica*) while the overall number of identified ORFs is only 1.6-to-2 fold higher than in yeasts. On the other hand, the numbers of SET-domain containing genes in the Pezizomycotina species (13–23) are about 3-fold higher compared to those of the yeasts (5–6; Fig. 3). Non-linear increasement in the number of *SET-domain *genes is even more pronounced in the animal and plant genomes: about 70 predicted *SET-domain *genes in the human and mouse genomes, around 40 in *D. melanogaster *[20], and around 40 in *A. thaliana *[21]. Increased numbers and diversification of encoded SET-domain proteins reflect, perhaps, increased diversity and complexity of multicellular programs.

Despite the smaller number of ORFs than most Saccharomycotina fungi [22], the single-living Taphrinomycotina (archiascomycete), *S. pombe*, carries three *SET-domain *genes (*SET9, MYND-SET*, and a *Su(var)3-9 *homologue). These *SET-domain *genes are present in the Pezizomycotina and in animals/plant, but remarkably absent from the other yeasts (except *Y. lipolytica*, which carries a *SET9 *gene) (Fig. 3). Presence of these genes supports a conclusion that *S. pombe *and *Y. lipolytica *share many common features with the filamentous relatives [22,23]. Lastly, *S. pombe *and *Y. lipolytica *carry one copy of a *SET-domain *gene of unclear origins. These *S. pombe*-specific and the *Y. lipolytica*-specific putative SET-domain proteins, unrelated to each other or to any of the 182 sequences analyzed, are annotated as 'unknown' (Fig. 3) and were excluded from further analyses.

Saccharomycotina genomes have been subjected to expansions (whole-genome duplications) and deletions (reductive evolution), which have shaped the genome sizes of extant yeasts [23,24] varying between 9.2 Mb (*A. gossypii*) to 20 Mb (*Y. lipolytica*) and overall putative gene numbers between ~4,700 and ~7,000 (Fig. 3). However, the total number of the *SET-domain *genes in all tested Saccharomycotina is remarkably constant: five or six. Most yeast *SET-domain *genes are single-copy representatives of distinct subgroups. The *SET4 *gene, an apparent duplication of *SET3 *(see below for further discussion) accounts for the difference in the number of *SET-domain *genes of *S. cerevisiae *and *C. glabrata versus *other yeasts; *SET6 *might have been deleted from the genome of *Y. lipolytica *or, alternatively, a duplication of *SET5 *to yield *SET6 *might have taken place, after the separation of *Y. lipolytica *from the other yeasts [23]. We note also that both *SET5 *and *SET6 *are absent from *S. pombe*, indicating that these genes encode functions limited to the specific needs of the Saccharomycotina group.

### Distribution of each SET-domain group among fungi

Analyses of the distribution of the fungal *SET-domain *types will be carried out in the context of the transition from unicellular to multicellular filamentous, and to animal/plant multicellular systems. We shall follow genes that are present in: all studied genomes, Saccharomycotina-specific genes, families that are excluded from the Saccharomycotina, and those that are found in specific fungal groups.

#### 1. *SET-domain *genes present in all studied genomes

Genes preserved in all tested genomes, across the kingdoms, suggest that they are involved in 'core' cell functions rather than in functions associated with multicellularity. Proteins that satisfy this criterion belong to SET1, SET2, and SET3 subfamilies (Fig. 3). It is specifically noted that these subfamilies belong in larger families containing groups that might have evolved later, possibly in connection with multicellularity. Fungal SET-domain proteins are analyzed in reference with the *S. cerevisiae *families because they have been studied best and because the *SET-domain *genes of this yeast have been a model and a reference for histone lysine methyltransferase analysis in multicellular systems.

##### The SET1/TRITHORAX family

**Figure 4:**
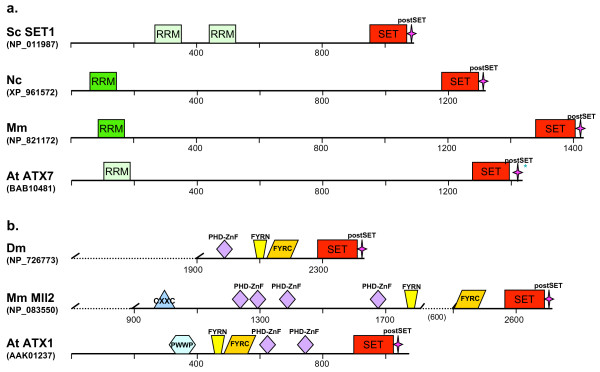
**Domain architecture of the SET1/TRX family (a: the SET1 subfamily, b: the TRX subfamily).** The divergent RNA recognition motifs (RRM) found in *Arabidopsis *and yeast (not identified in domain databases) are indicated by the pale color in the figure. Domains are not drawn to scale. For the TRX subfamily, representatives of two major animal subgroups differing by the positioning of the FYRN-FYRC (DAST) domains are shown. Note the presence of conserved domains between the animal and the plant representatives. For more structures see Additional file 3.

The *SET1 *gene of *S. cerevisiae *encodes a member of the large TRITHORAX family (named after the *Drosophila *Trithorax protein). The SET domain and the adjacent cysteine-rich motif (post-SET) are the two most highly conserved sequences defining a protein's belonging to the family (Fig. 4). Two subfamilies, the SET1 and the Trithorax (TRX), are distinguished here. We note that in all fungi examined (Saccharomycotina, Pezizomycotina, as well as *S. pombe*), the family is represented by a single copy of the SET1-type; by comparison, animal/plant genomes contain several genes from this family, including the SET1- and the TRX-subtypes. Distribution of SET1/TRX proteins is summarized also in Fig. 2a.

##### The SET1 subfamily

The ancestral gene encoding SET1 in *S. cerevisiae *has been conserved throughout the evolution and is present in all species examined in this study (Fig. 3). The SET-post-SET sequences (located at the C-terminus) are highly conserved in all examined SET1 proteins (Fig. 4a, see also Additional file 3). In the Saccharomycotina, the upstream regions are highly conserved as well: *C. glabrata *and *A. gossippi *are 56% similar (37% identical) and 54% similar (34% identical) to the upstream *S. cerevisiae *sequence, respectively; the region is more diverged between the Saccharomycotina, *S. pombe*, and the Pezizomycotina fungi, the similarity with *S. cerevisiae *ranging from 41 to 39%, respectively. The upstream regions contain an RRM (RNA-recognition motif) [25,26], which may affect the level of K4 methylation [27,28]. Most likely, it does not bind RNA but may interact with protein, instead [29,30]. Congruent with the domain architecture, the fungal SET1 proteins form a well-supported group (SET1 group 3 in Fig. 1) with the *S. pombe *SET1 as the most outgroup. SET1-related proteins, with conserved RRM upstream of the SET domain, are present also in the animal and plant genomes suggesting preservation of the *SET1*-encoded function in the evolution from fungi to metazoa/plants. One gene copy in *Drosophila *encodes a SET1 homologue (EAL24599, identified by the Heterochromatin Genome Project, which was recently released and not included in the sequence set we analyzed), whereas in mouse, as well as in human, the gene has undergone duplication (see Additional file 2); in *Arabidopsis*, the *ATXR7 *gene (*At5g42400*) encodes a SET1 counterpart (BAB10481). SET1-homologues of animal and plant origin cluster in SET1 group 2 with bootstrap supporting values between 60% and 70% (Fig. 1).

Thereby, if the conserved *SET1*-related genes from the genomes of unicellular fungi, of filamentous fungi, and of metazoa/plant were orthologues, they would play 'core' cellular roles not connected with the occurrence of multicellularity. This does not preclude involvement of SET1-related genes in regulation of development in multicellular organisms.

##### The Trithorax (TRX) subfamily

This group, shown as SET1 groups 1 and 4 in Fig. 1, contains no gene of fungal origin and will not be discussed in detail. However, we emphasize that in animal/plant genomes, an ancestral *SET1-*related gene has multiplied and diversified its structure and, most likely, function. The PHD (plant homeodomain) and the FYRN- and FYRC-domains (collectively called Domain associated with SET in Trithorax, DAST, in [31]) are considered to be signature motifs for the members of the Trithorax subfamily (Fig. 4b). It is noted that the two subgroups of animal Trithorax proteins differ by the position of the FYRN- and FYRC-domains (juxtaposed or separated), while the plant Trithorax homologues, ATX3, ATX4, and ATX5, do not carry DAST motifs [31]. Hallmark of the SET1/Trithorax family proteins is their biochemical activity methylating histone H3-lysine4 (H3K4). SET1 is responsible for the overall chromatin mono-, di-, and tri-methylation of H3K4 in *S. cerevisiae*, while known animal and plant Trithorax enzymes modify only a limited fraction of target nucleosomes [32-34]. The roles of the additional motifs are largely unknown but acquisition of new building blocks reflects the evolution of the proteins in parallel with the requirements for novel functions emerging in animals and plants.

##### The SET2/ASH1 family

**Figure 5:**
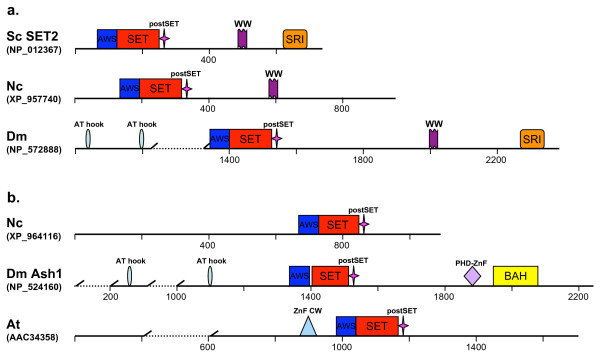
**Domain architecture of the SET2/ASH1 family (a: the SET2 subfamilies, b: the ASH-1 subfamily).** Domains are not drawn to scale. For more structures see Additional file 3.

All proteins from this family carry a SET domain (sufficiently different from that of SET1 family) preceded by a signature cysteine-rich peptide called AWS (associated with SET) (Fig. 5, see also Additional file 3). A single *SET2 *gene is present in the Saccharomycotina fungi and in *S. pombe*, two *SET2*-related genes are found in the filamentous fungi, and multiple copies are present in animal/plant genomes. Conserved in all examined species, the SET2/ASH1 proteins form a monophyletic group divided further into two major subfamilies as summarized in Fig. 2b.

##### The SET2 subfamily

Apparently, these genes are of an ancient origin existing before the divergence of the three kingdoms. They are present in all examined genomes (Fig. 3, SET2 group 2 in Fig. 1). In addition to the AWS-SET domains, all fungal members as well as animal (both vertebrate and invertebrate) members of the SET2-subgroup 2 carry a downstream WW-peptide participating, most likely, in protein-protein interactions and the SRI motif mediating RNA polymerase II interaction (Fig. 5a) [35,36]. It is conceivable, then, that *SET2 *subtype genes have been conserved for RNase polymerase II-interaction as a core cellular function and, thus, unlinked with multicellularity. We note also that the sole *S. pombe *SET2 copy (NP_594980) does not carry the WW motif. The *Arabidopsis *SET2 homologue candidates either cluster with animal SET2 proteins (AAC34358) or are located as the outgroup (AAF04434, as well as CAA18207 and AAC23419) (Fig. 1). The SET2 subfamily also includes metazoa-specific groups: NSD (nuclear receptor binding SET domain protein) and WHSC (Wolf-Hirschhorn syndrome candidate) (represented by NP_001001735 in Fig. 1; see also Fig. 2b).

##### The ASH1 subfamily

The second *SET2 *gene from the filamentous fungi was defined as an *ASH1*-family member by phylogenetic analysis (SET2 group 1 in Fig. 1). The family is named after the ASH1 protein of *Drosophila*. Notably, the fungal ASH1-members do not carry recognizable structural motifs other than SET, post-SET, and AWS, while animal and plant members contain additional domains (Fig. 5b, see also Additional file 3).

The SET2 family cluster is well supported (91% bootstrap value by maximum likelihood phylogeny) indicating that the SET2-ASH1 paralogues are phylogenetically and structurally closely related. It is noteworthy then, that the proteins from the two subfamilies have histone methyltransferase activities with different substrate specificities. The SET2 methyltransferases of *S. cerevisiae *and *S. pombe *are responsible for mono-, di-, and tri-methylation of H3K36 [35,36]; the SET2 orthologue in *N. crassa*, required for normal growth and expression of genes in the asexual and sexual differentiation pathways, also methylates H3K36 [37]. However, the epigenetic activator ASH1 of *Drosophila *is a multi-catalytic histone methyl-transferase (HMTase) methylating H3K4, H3K9, and H4K20 [38], while the activity of an ASH1-related protein has not been reported for any orthologue of fungal origin.

##### The SET3 family

*SET3*-related genes are found as single copies across the genomes of yeasts, multicellular fungi and animal genomes suggesting that a *SET3 *gene has existed in the common ancestor before the separation of the animal and fungal domains of life (summarized also in Fig. 2c). The fungal SET3 proteins form a well-supported cluster with subgroups, each carrying proteins from the Saccharomycotina fungi, the Pezizomycotina (filamentous) fungi, or animals. Amino acid substitutions in the catalytic site by arginine (R) residues are a hallmark of SET3-type proteins. The unusual SET domain (**RRSC**QPN, see Additional file 4) divergent from the motif critically involved in the HMTase enzyme function (**NHSC**DPN) accounts for the lack of histone methyltransferase activity of the *S. cerevisiae *SET3. However, it is a component of a histone deacetylase complex involved in the meiosis-specific repression of sporulation genes [39]. In this context, it is difficult to predict the role of metazoan SET3 counterparts but their conservation implies biological significance.

Two *Arabidopsis *proteins, ATXR5 and ATXR6 (NP_196541 and NP_197821, respectively), were reported earlier to be clustering with the *S. cerevisiae *SET3 and SET4 proteins [40]. However, in our analysis, the *Arabidopsis *proteins did not segregate with the yeast SET3/4 (see the draft phylogeny in Additional file 2). Furthermore, a detailed comparative analysis with the yeast sequences did not reveal significant conservation. Most importantly, the *Arabidopsis *SET domain sequences do not carry the hallmark amino acid substitutions. ATXR5 and ATXR6 interact with the proliferating cell nuclear antigen (PCNA) and are critically involved in DNA replication, DNA repair, maintenance and heterochromatin formation [41].

##### The SET4 family

**Figure 6:**
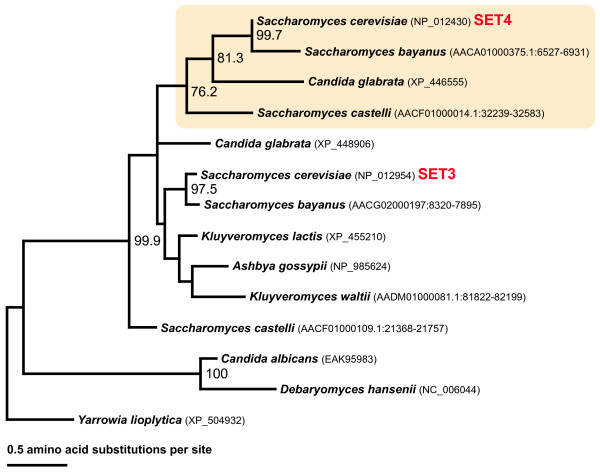
**Maximum likelihood phylogeny of the SET3/4 families among ten Saccharomycotina species.** Bootstrap values greater than 60% are shown. The genomic position is indicated with accession numbers for the sequences from unannotated genomes. The gamma-shape parameter and the proportion of invariant sites were estimated to be 0.879 and 0, respectively.

The SET4 proteins are phylogenetically related to SET3, clustering together with 95% bootstrap value (Fig. 1). However, SET4 proteins are found only in *S. cerevisiae *and its closest relatives. A whole-genome duplication event in the history of *Saccharomyces *has generated 'twin genes' found in the genomes of extant species [42] and it is plausible that *SET3 *and *SET4 *genes are a consequence of this event. To elucidate the timing of the putative SET3/4 duplication, we performed additional similarity searches in *S. cerevisiae *and nine other Saccharomycotina species. SET4 proteins were found in *S. bayanus*, *S. castelli*, in addition to *S. cerevisiae *and *C. glabrata*. The result is consistent with reports placing the whole genome duplication after the divergence of related species: *A. gossypii*, *K. lactis*, and *K. waltii *[24,42]. The maximum likelihood phylogenetic tree is also consistent with a SET 3/4 duplication occurring in the ancestral lineage leading to *Saccharomyces *species and *C. glabrata *(the shaded cluster in Fig. 6). Furthermore, the SET3 and SET4 sequences are more highly conserved between the *S. cerevisiae *and *S. bayanus *compared to *S. castelli *suggesting that *SET3 *gene duplication could place the whole-genome duplication event before the divergence of *C. glabrata *and the *Saccharomyces *species.

##### The JmjC family

Although JmjC-SET/SET3 cluster is not supported by bootstrap analysis (Fig. 1), the structural relationship between the SET domains of JmjC-SET and the yeast SET3/4 families is reflected by the characteristic R-substitutions in the catalytically relevant sequences (see Additional files 4 and 5). In contrast to SET3/4 families, this family is found only in filamentous fungi. It is represented by a single gene in *N. crassa *and in *A. fumigatus*, by a duplication in *M. grisea*, but has not been identified in *F. graminearum*. Therefore, the activity encoded by *JmjC*-*SET *plays subphylum-specific role but may be species-specific as well.

None of the known JmjC proteins appears together with a SET-domain outside the filamentous fungi. The striking feature of this combination is that the JmjC domain is a catalytic module for histone lysine-demethylation [43,44]. Although their activity is evolutionarily conserved from human to yeast, as shown recently for seven JmjC proteins from *S. pombe *[45], none of these proteins appears together with a SET. It re-enforces the conclusion that the combination of the two motifs is a feature uniquely occurring only in the filamentous fungal proteins. Whether the SET domain of JmjC has a methyltransferase activity has not been demonstrated. However, its possible relatedness with the SET domain from the SET3/4 families suggests that it might not be active as a histone methyltransferase.

#### 2. The SET5, SET6, and related families

Based on our phylogenetic analysis (Fig. 1), the distribution of SET5, SET6, and related families is summarized also in Fig. 2d. The yeasts' SET5 and SET6 families belong in a larger cluster that includes families represented across the kingdoms (SET-MYND), as well as families specific for the filamentous fungi (SET-TPR), and species-specific groups (SET-Mg and SET-Dm). Apparently, the cluster has originated from an ancestral gene that has undertaken different evolutionary paths at the separation of the fungi from the other kingdoms and at the separation of the Ascomycetal sister groups.

##### The SET5 and SET6 families

*SET5 *and *SET6*, each segregating into a well-supported cluster, are yeast-specific genes absent from the genome of *S. pombe*. *SET6*, implicated in ergosterol biosynthesis [46], is absent from the more distant *Y. lipolytica *suggesting that the gene encodes a narrow Saccharomycotina-specific function. *SET5 *and *SET6 *encode related ~500 amino acid proteins containing the SET domain as the only recognizable conserved structural motif. A signature feature is the long SET-I (insertion region of ~125 a.a.) producing a split SET domain. No role has been established for SET5 yet but, apparently, it is yeast-specific.

##### Filamentous fungal genes related to the SET5/SET6 families

Several copies of genes encoding SET-domain proteins similar to the SET5/6 protein group were retrieved from the genomes of the filamentous fungi (shown in the draft phylogeny in Additional file 2). These genes encode relatively short (300–500 a.a.) proteins containing only SET domains. They were not included in Fig. 1 because their sequences could not be aligned with confidence. No function is known for any of these proteins, but absence of apparent orthologues from yeast, as well as from animal/plant genomes, illustrates a highly specific evolution of these genes in the filamentous fungal genomes. In addition, a highly supported (99% bootstrap value) cluster of five *SET-*domain genes was found in *M. grisea*, encoding species-specific (SET-Mg) proteins. It is interesting to note also the *D. melanogaster *specific cluster (SET-Dm) in this group suggesting an intriguing property of the *SET5/6*-related genes towards proliferation and adaptation for species-specific needs by diverse organisms.

##### The TPR-SET family

Found only in the filamentous fungi, this family is a combination of a SET 5/6-related domain with an upstream tetratrico- peptide repeats (TPR, involved in protein-protein interactions) [47]; two members of the family are present in *M. grisea *and *F. graminearum *genomes. We note that one *Arabidopsis *gene (*At1g26760*) encodes a SET-domain protein combined with TPR (AAF87042), which clusters with the fungal TPR-SET cluster (Fig. 1). However, this relationship is not strongly supported, making their evolutionary relationship unresolved.

##### The MYND-SET family

This family is noteworthy because it contains genes found in *S. pombe*, in the filamentous group, in metazoa, and in *Arabidopsis*, but not in the Saccharomycotina fungi. The SET domains of the MYND-SET family form a well-supported cluster within the larger SET5/SET6 family (Fig. 1), suggesting shared ancestry. MYND-SET proteins carry a Zn-binding domain called the MYND-finger (myeloid, nervy, and DEAF-1 factor) involved in H3K36- and in H3K4-specific methylation [48,49]. The metazoan subfamily, SMYD, is critically involved in suppressing cell proliferation and carcinogenesis [48,49]. No role is known for any MYND-SET protein of either plant or fungal origin.

#### 3. Gene families conserved in all genomes, except in the Saccharomycotina

The SUV3-9 and SET9 (SUV4-20) families are represented in the genomes of the unicellular *S. pombe*, in the Pezizomycotina, as well as in the animal and plant genomes, but are not found in the Saccharomycotina. Absence from the entire Saccharomycotina group suggests that these genes are unlikely to be involved in 'core' functions but, rather, in mechanisms used by the fission, filamentous, and multicellular eukaryotes; alternatively, these orthologous genes might be used differently and, consequently, be involved in different cellular processes in the fungi than in animals and plants (discussed below).

##### The SUV3-9 family

Genes of this family have undergone extensive proliferation in animal and plant genomes, particularly in *Arabidopsis *(Fig. 3) [21]. The family is divided into multiple subgroups illustrating its internal heterogeneity (Fig. 1) [34,50]. Here, analysis will be limited to the two subtypes, Su(var)3-9 and G9a, because they are relevant for Ascomycota (summarized also in Fig. 2e).

##### The Su(var)3-9 subfamily

**Figure 7:**
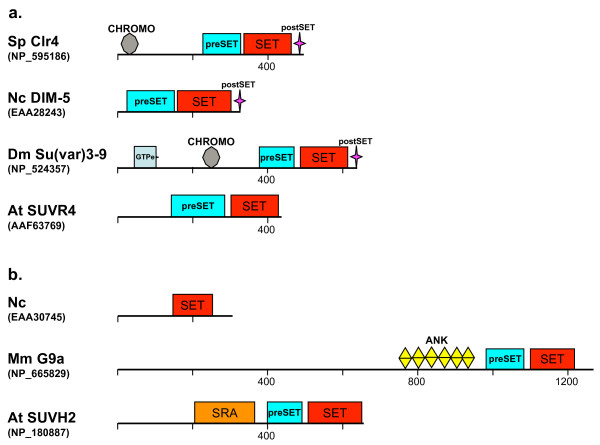
**Domain architecture of the SUV39/G9a family (a: the SUV39 subfamily, b: the G9a subfamily).** Domains are not drawn to scale. For more structures see Additional file 3.

The defining feature of the proteins (group 3 in the SUV3-9 cluster in Fig. 1) is the Su(var)3-9-type of SET domain and the pre- SET (PRS) motif located immediately upstream of the SET domain (Fig. 7a, see also Additional file 3). PRS contains nine invariant cysteine residues, coordinating three zinc ions, involved in the structural stability of the SET domain [51]. The Su(var)3-9 protein discovered initially in *D. melanogaster *(NP_524357) is the founding member of the family. It belongs in a subgroup that carries an additional CHROMO domain. It is important to point out that the proteins of filamentous fungal origin (group 4), as well as the *Arabidopsis *family members (group 1; or the SUVR subgroup in [21]), do not have the CHROMO domain but, nonetheless, belong in the Su(var)3-9 subfamily based on the SET-domain phylogeny (Fig. 1) and the pre-SET domain (Fig. 7a). A notable exception among the fungi is the *S. pombe *protein, which contains a chromodomain.

The Su(var)3-9 subfamily proteins catalyze methylation of K9 of histone H3 [2] essential for heterochromatin formation in metazoa [52] and in *S. pombe *[53], and link histone H3K9 methylation with DNA methylation in *N. crassa *[54-56]. Absence of *Su(var)3-9 *genes from the Saccharomycotina fungi has had enormous consequences for the evolution of heterochromatin-like gene-silencing mechanisms in yeast (see Discussion).

##### The G9a subfamily

Discovered initially in the human major histocompatibility complex locus, the *G9a *genes are ubiquitously expressed. It is represented by a *M. musculus *sequence (NP_766133) in the SUV3-9 group 3 in Fig. 1 (it forms a small cluster including *D. melanogaster *sequence in Additional file 2). The mammalian G9a proteins contain pre-SET and SET domains of the Su(var)3-9-type, plus ankyrin (ANK) repeats (Fig. 7b, see also Additional file 3) with a strong HMTase activity towards H3K9. In contrast to its paralogue Su(var)3-9, G9a plays a role as a transcriptional suppressor of genes in euchromatic regions but not in heterochromatin [56]. *Arabidopsis *G9a proteins (group 2 in the SUV3-9 cluster in Fig. 1) carry SRA (SET and RING finger associated domain) instead of the ANK repeats. These *Arabidopsis *genes have proliferated (at least 10 genes belong to the this subfamily [21]) and are involved in mediating DNA methylation [57-59]. Among the examined fungi, an *N. crassa *protein (EAA30745) showed weak similarities against mammalian G9a SET-domain sequences (~30% identities). This fungal protein has only the SET domain (Fig. 7b). The phylogenetic relationship of the *N. crassa *sequence (EAA30745) has not been resolved (see the draft phylogeny in Additional file 2).

##### The SET9 (SUV4-20) family

The fungal *SET9 *genes belong to the *SUV4-20 *family characterized in metazoa [60,61]. Due to the lack of confidence in alignment, this family is not included in Fig. 1. In the draft phylogeny (see Additional file 2), these sequences are clustered and indicated in blue. Putative SET9 homologues are present in *S. pombe *(NP_588078) as well as in the filamentous fungi. Members of the family are absent from yeasts, except *Y. lipolytica *(XP_504243). *SET9*/*Su(var)4-20 *genes have not beenfound in *Arabidopsis *(nor in rice nor maize).

Proteins from the SUV4-20 family carry only a SET domain located at the N-terminal half (see Additional file 3). These genes encode histone methyl transferases, specifically tri-methylating H4K20, an evolutionarily conserved epigenetic mark for heterochromatin [61]. Remarkably, in the fission yeast, SET9 generates methylated H4K20 but it is used as a sign for DNA repair mechanisms, not for heterochromatin [62].

#### 4. The Enhancer of zeste (E(Z)) family: a SET-domain family found only in the filamentous fungi and in higher multicellular organisms

In our analysis, *E(z)-*type genes were not found in the Saccharomycotina nor in the *S. pombe *genome. It is tempting to suggest that the ancestral *E(z) *gene has appeared as a novel SET-domain function inherited by extant filamentous fungi, animals, and plants (see also Fig. 2g). The structure of the E(z)-SET domain and its biochemical activity (see Discussion) are consistent with the idea that the gene has appeared later in the evolution.

Three proteins found in the filamentous fungi (XP_381075, EAA35807, and XP_369092) cluster with the animal/plant E(z) family but with low bootstrap values (51% by the maximum likelihood and 79% by the maximum parsimony). No *E(z)*-related gene was identified in *A. fumigatus*, possibly representing gene-loss (incomplete genome sequence is also possible). The fungal proteins do not carry additional structural domains, which are uniquely conserved in the E(z) proteins of animal and plant origin (see Additional file 3) and belonging of the fungal genes to the *E(z)*-type group is inconclusive (marked with a star in Fig. 2g). E(z) methylates specifically H3K27 within Polycomb PRC2 complexes, a landmark for gene silencing mechanisms [63,64]. Absence of *E(z)*-related genes in the unicellular yeasts indicates that Polycomb mechanisms do not operate in these systems.

## Discussion

Correlations between genome evolution, overall gene content, and organismal complexity, revealed in whole-genome comparative analyses, have outlined evolutionary trends associated with the occurrence of multicellularity [17,65]. It was suggested that core biological functions, common for both unicellular and multicellular organisms, would be carried out by a comparable number of orthologous proteins, while specialized processes unique to multicellulars would use novel proteins [66]. Our analyses support a positive correlation between the numbers and types of *SET-domain *genes found in multicellular versus unicellular fungi; a similar tendency was observed when the simpler multicellular (filamentous) fungi were compared with multicellular animal and plant genomes. Analysis of the distribution of individual *SET-domain *fungal genes, however, revealed some unexpected trends, particularly within the Saccharomycotina group.

### Morphology, habitats, mating mechanisms and pathogenicity of yeasts and their *SET-domain *gene contents

Despite a broad range of lifestyles, life cycles, and mating mechanisms [67], the numbers of *SET-domain *genes in the respective yeast species is remarkably constant and the gene structure is highly conserved. Neither the large *Y. lipolytica *genome size nor the extensive gene-loss in *C. glabrata *is reflected by the numbers of the *SET-domain *genes suggesting that in yeasts, the collection of *SET-domain *genes has been selectively maintained. The permanently filamentous yeast *A. gossypii *displays high genomic synteny with *S. cerevisiae *but has a very different morphology and occupies a specific niche: a plant pathogen. *A. gossypii *grows as multinucleated hyphae in the subtropics, while *S. cerevisiae *proliferates as single cells associated with sugar-containing fruits [23,67-69]. *C. glabrata*, closely related to *S. cerevisiae*, is a pathogen living on mucosal human tissues, while the more distant pathogenic *C. albicans *grows as yeast but may switch to hyphal growth when exposed to host serum. Genes from pathogenic species that differ from *S. cerevisiae *might have a role in the virulence [23,70]. Despite the strikingly different biology of thee yeasts, their *SET-domain *genes are fully shared suggesting orthologous functions. In such a context, the yeast *SET-domain *genes cannot be considered critical for the dimorphic transitions, for hyphae formation, for long-range nuclear dynamics, or as factors contributing to the pathogenicity.

*SET-domain *genes do not seem to be underlying differences in the life cycles and sexual mechanisms adopted by the individual species, either. *D. hansenii *is homothallic with essentially haplontic life cycle and *Y. lipolytica *is heterothallic (self-sterile) with a haplo-diplontic cycle, while sexual cycles are unknown for *C. glabrata *[23,67] and, until recently, for *C. albicans *[71]. Although *C. albicans *has mating-type-like (MTL) genes that resemble the mating-type genes of *S. cerevisiae*, *C. albicans *is able to mate under anaerobic conditions reflecting its adaptation as an anaerobic parasite [72]. Finally, *D. hansenii *and *Y. lipolytica *have one mating type-locus, whereas *C. glabrata *possesses three mating type-like loci with configurations similar to that of *S. cerevisiae *[73]. Given the importance of the diversity of sexual mechanisms for the evolution of the species, it is remarkable that these mechanisms are, most likely, not connected with the evolution of the yeast *SET-domain *genes. We emphasize, however, that this conclusion does not preclude participation of *SET-domain *genes in these processes; rather, it suggests that the different biology of the species is not connected to specific diversifications of the *SET-domain *genes *per se*. It agrees with observations from whole-genome analyses that almost identical gene sets control diverse cellular functions in yeasts suggesting that orthologous genes might not play identical cellular roles in different yeast systems [23,67,69]. Differences, like the presence of SET4 in *S. cerevisiae *and *C. glabrata *and the absence of SET6 from *Y. lipolytica *are, most likely, connected to the fermentative lifestyle [74] and to metabolic specificities of organisms, rather than with differences in the morphology, in sexual mechanisms, or in pathogenicity.

### Evolution of the activating and repressive marks in the fungi; Consequences of lost *SET-domain *genes in Saccharomycotina

The *SET9 *(*SUV4-20*), *SET-MYND*, and *Su(var)3-9 *genes, found in *S. pombe*, in filamentous fungi, in animals, and in plants, but absent from the Saccharomycotina, suggest that the encoded activities are not involved in 'core' functions. They are involved in generating marks for silencing mechanisms implying that these silencing mechanisms do not exist in the Saccharomycotina. SET9 (SUV4-20) tri-methylates H4K20, a mark for pericentric heterochromatin [61] and MYND-SET family members (studied only in animals so far) generate H3K36 marks involved in suppression of cell proliferation [48,49], while SUV39 methylates H3K9 critical for heterochromatin formation [52]. Recent findings have suggested that epigenetic 'ON' marks are reduced in evolution, while the 'OFF' signs have significantly increased; unicellular organisms contain more marks associated with transcriptional activation, whereas mammals contain more modifications associated with repression [5,6]. Indeed, the only known histone methylation marks in *S. cerevisiae *(H3K4, H3K36 and H3K79) and the enzymes generating them (SET1, SET2, and DOT1, an unrelated to SET domain that methylases K79) are associated with gene-activation [75]. Absence of *SET9, MYND- SET*, and *Su(var)3-9 *genes from the Saccharomycotina is consistent with this evolutionary trend. However, their presence in *S. pombe *(and of *SET9 *in *Y. lipolytica*) suggests that the 'silencing' marks established by the encoded activities are not signature features of multicellular genomes; moreover, the epigenetic marks might be 'read' differently. For example, SET9 of *S. pombe *methylates H4K20 but it is a sign for DNA damage response in *S. pombe *[62] rather than for heterochromatin [60,61]. Absence of genes encoding silencing marks in the Saccharomycotina, thereby, raises an important question described next.

### How do yeasts silence genes and genomic regions?

Answers may be found in the remarkable ability of yeasts to adopt available means to achieve ends that are functionally similar but molecularly different from mechanisms employed by other systems. The most striking example is the loss of the *Su(var)3-9 *gene from the Saccharomycotina because loss of SUVR39 protein entailed loss of the epigenetic H3K9me mark, and of the entire machinery involved in making heterochromatin [56]. Nonetheless, silencing processes involving the MAT cassettes, the ribosomal loci, and the telomeric regions are achieved through mechanisms similar to the assembly of heterochromatin [76]. Even more surprising is that close *S. cerevisiae *relatives do not use the same tools but have evolved species-tailored mechanisms for achieving effects functionally similar to heterochromatin. There is no Sir1 (Silent information regulator 1) in *C. glabrata*, no Sir1 and Sir3 in *A. gossypii*; neither Sir nor the RNAi-pathways are conserved in *D. hansenii *and none of the *S. cerevisiae *heterochromatin factors was found in *Y. lipolytica *[67]. Collectively, the data illustrate the great evolutionary divergence of 'invented' mechanisms producing silencing effects in systems that have lost the epigenetic H3K9me mark.

Furthermore, loss of *SET9 *and *MYND-SET *related genes encoding gene-silencing functions has resulted in evolving mechanisms that take advantage of the degree (mono-, di-, or tri-) methylation of the lysine NH2- groups to achieve different transcriptional outcomes for pertinent genes. In *S. cerevisiae*, di- (H3K4me2) or tri- (H3K4me3) methylated lysines are associated with non-active, or actively transcribed sequences, respectively [77,78]; in *Arabidopsis*, H3K4me2 marks are found at genes, independently of whether they were transcribed or not, while the H3K4me3 marks were enriched at active loci [79]. In metazoa, both modifications are associated with actively transcribed genes but are differentially distributed along the gene sequence with the H3K4me3 marks accumulated at the transcription start-sites [20,33].

Thereby, the amount of methyl tags on the same lysine residue may function as repressive or activating signs in *S. cerevisiae*, while the fission, filamentous, and higher multicellular systems use additional signs carried by a larger number and diversity of methylation marks.

### *SET-domain *genes specific for the filamentous fungi

Consistent with their greater morphological and developmental complexity, the filamentous fungi have a greater number of genes than their unicellular relatives. Conserved proteins among the filamentous fungi may be implicated in their morphogenesis, while non-conserved, species-specific genes might be associated with pathogenic capabilities [16,17,80-85]. According to these criteria, the different types and numbers of *SET-domain *genes in the Pezizomycotina compared to the yeasts (Fig. 3) might encode functions linked with hyphal growth, mycelia development, or other processes underlying cellular morphogenesis and virulence. Furthermore, the genes from the JmjC-SET, TPR-SET, and SET5/6 related filamentous fungal genes encode fungal functions specific for the entire filamentous group, while the cluster of five-related genes present in *M. grisea*, but not in *N. crassa*, might be associated with life as a pathogen. Whether the *M. grisea*-specific SET-domain cluster is related to its pathogenicity is unknown. Variations in the *JmjC-SET *and the *TPR-SET *gene numbers in particular genomes (Fig. 3) are, most likely, due to species-specific deletion/duplication events. Whether these differences in copy numbers are linked with the pathogenicity of the species has not been demonstrated.

We note also the phylogenetic relationship between the filamentous fungi-specific TPR-SET and SET5/6 related proteins, and the Saccharomycotina-specific SET5 and SET6 proteins (Fig. 1). It illustrates the divergent evolutionary paths of a common ancestor at the separation of the two Ascomycota subgroups. Similarly, the phylogenetically related SET domains of the JmjC-SET and SET3/SET4 subgroups suggest that they result from subphylum-specific divergence of a shared ancestor. The *SET3*-lineages has been conserved throughout the evolution and its duplication in *S. cerevisiae *and *C. glabrata *has produced *SET4*, while duplication in the filamentous group has produced the JmjC-SET cluster (Fig. 2c).

### *SET-domain *genes found exclusively in multicellular organisms

*SET-domain *genes of multicellular organisms not found in any unicellular fungi may be involved in multicellular, rather than in 'core' functions. Members of the ASH1, the G9A subfamilies, and of the E(z) family are such candidates.

In contrast to the 'core' function encoded by *SET2*, the *ASH1*-related copy found in the filamentous fungi has relatives only in multicellular organisms. The descendants of the ancestral *ASH1 *lineage in extant animal and plant genomes has evolved further by acquiring additional structural motifs. This process could be linked with the evolution of more complex morphology and communication systems operating in the higher multicellular systems.

No paralogues of the *E(z) *genes have been recognized in the genomes of unicellular yeasts suggesting a later occurrence in the evolution. The structure of the E(z)-type SET-domain peptide may illustrate a discrete step in the evolution of the SET-domain protein structure/function. A Zn-finger involved in substrate specificity of histone methyltransferases [86] is formed by the Cys in the consensus NHXC sequence and the post-SET domain motif (CxCxxxxC) conserved in a subset of SET-domain proteins. Loss of this structure is, most likely, a secondary event triggered by a single substitution of the C in the NHXC box leading to the loss of the post-SET domain and subsequent divergence of the biochemical specificity.

Occurrence of E(z) might be linked with the transition to multicellularity, as suggested by the lack of K27 marks and *E(z) *genes from the unicellular yeasts, *Dictyostelium*, and *Chlamydomonas*. However, abundant H3K27me marks were found in *Tetrahymena *[5], suggesting that H3K27me marks have evolved for species-specific needs as well. It will be very informative to establish the roles of the *E(z) *genes in *Tetrahymena*, particularly in view of the ability of animals and plant E(z) paralogues to assemble specific Polycomb-group complexes [87-90]. Furthermore, H3K27me and H3K4me marks establish a bivalent chromatin state at the nucleosomes of developmentally regulated genes in animal stem cells [91] and in *Arabidopsis *[92], defining them as histone marks for differentiation processes.

Particularly important is the presence of conserved peptide domains related to the SANT-domain family, present in the E(z) proteins of animal and plant origins. Furthermore, in the reconstructed phylogeny based solely on SET-domain sequences, the E(z) animal and plant proteins cluster together with very high bootstrap values (99%). The conserved structure suggests that the last shared ancestor for the *E(z)-*lineage carrying these domains has existed before the divergence of the animal and plant genes. This putative ancestor has either originated after the separation of the fungal lineage, or has been deleted in the fungal ancestor. A similar pattern was noted for the evolution of the *TRX *genes, suggesting that the PcG/TrxG mechanism has been evolutionarily conserved in the animal and plant kingdoms but not in the fungal kingdom.

### Origin of the 'orphan' *SET-domain *genes in the filamentous fungi

Recent large-scale comparison of sequence data has found over 40 *N. crassa *genes with no identifiable *S. cerevisiae *homologues [16]. It was suggested that these "orphan" genes might have resulted from either loss of related genes from the *S. cerevisiae *lineage, from horizontal gene transfer into the *N. crassa *lineage, from newly generated 'innovative' genes, or from exceptional gene-divergence in *S. cerevisiae *[16]. Our analysis is congruent with processes of deletion from the Saccharomycotina genomes and with the assembly of novel genes in the filamentous fungi, as credible fates for the evolution of orphan *SET-domain *genes.

The *SET9*, *SET-MYND*, and *Su(ver)3-9 *genes appear to have been lost from the Saccharomycotina genomes or from the lineage leading to the Saccharomycotina. Supporting evidence comes from the finding of highly conserved sequences for each of these three genes in *Rhizopus orizae*, which is an outgroup to Ascomycota and a member of the basal group, Zygomycetes. Existence of the orthologous protein candidates in the *R. orizae *genome (RO3G_14129: 48% identical and 66% similar to *S. pombe*'s SET9, RO3G_13695: 25% identical and 41% similar to *S. pombe*'s MYND-SET, and RO3G_16553: 43% identical and 59% similar to *S. pombe*'s *Su(ver)3-9*) suggests that ancestors of these genes have existed before the divergence of the Ascomycetes, and subsequently have been lost from the Saccharomycotina. The yeast *SET9-*gene lineage has been lost even later, after the divergence from *Y. lipolytica*.

An interesting feature in the evolution of the *Su(var)3-9 *gene is the presence of the chromodomain in animal and *S. pombe *proteins. Because a chromodomain is absent from the proteins of the filamentous fungi and plants, it was suggested that the ancestral form, before the divergence of animal and fungal lineages, has carried the combination of the two motifs but the chromodomain has been lost from the filamentous fungi after their separation from the fission yeast [50]. However, absence of a chromodomain in the *Su(var)3-9 *orthologue in *R. orizae *and in *Ustillago *(a member of the sister group Basidomycetes) suggests an alternative scenario: the ancient fungal *Su(var)3-9 *lineage did not have a chromodomain and acquisition of chromodomain encoding sequences by the *S. pombe *and animal *Su(var)3-9 *genes were two independent events. *Su(var)3-9*-related genes have remarkably proliferated in *Arabidopsis*, but no plant homologue carries a chromodomain.

Homologues of *E(z), SET2(ASH1*)-like, *G9a*-like, *JmjC-SET, and TPR-SET *genes were not found in the *R. orizae *genome. It is plausible that these genes have occurred in the Pezizomycotina, required by multicellularity-related functions. The mechanism of genetic innovation in the filamentous lineage is unclear. Predicted trends underlying the occurrence of novel proteins may include evolution of new protein architecture from preexisting domains (including reshuffling of existing domains) and/or expansion of particular domain families by series of duplications, followed by specialization, to meet the specific needs of a species. It is clear that the *JmjC-SET and TPR-SET *genes have been retained in the Pezizomycetes for uniquely fungal processes, while the *E(z), SET2 (ASH1*), and *G9a*-lineages have been inherited in the animal and plant ancestors, where they have evolved further (Fig. 2).

The limited number of animal and plant *SET-domain *genes included here represent descendants of lineages found in the unicellular fungal group (*SET1, SET2, SET3, Su(var)4-20, Su(var)3-9*, and *MYND-SET*) and of lineages found in the filamentous group (*ASH1*, *G9a*, and *E(z)*). Distinct subgroups, shared by animals and plants, have evolved within the larger families existing in the fungi (*i.e*., Trithorax, E(z)), as well as plant- or animal-kingdom specific lineages [21,93-95]. Furthermore, some animal-specific SET-domain families (SET8) are distributed throughout metazoan genomes, while others (SET7) are found only in vertebrates.

### In Summary

The phylogenetic analysis allowed us to trace clear distinctions between species-, subphylum-, and kingdom-specific SET domains, as well as to recognize factors involved in core-cellular roles *versus *those likely to be associated with multicellular requirements. Surprisingly, the collection of *SET-*domain genes within yeast did not appear critical for differences in lifestyles, abilities to morph, sexual mechanisms, and pathogenicity of hemiascomycetes. However, *SET4, SET5*, and *SET6 *encode Saccharomycotina-specific functions and appearance of *SET4 *parallels the genome duplication of *Saccharomycetes *believed to be important for their fermentative abilities [42]. *SET-domain *genes found in the filamentous species, but absent from the unicellular sister group, reflect two evolutionary events: deletion from the yeasts genomes (*SET9, MYND-SET*, and *Su(var)3-9*) and appearance of novel structures. The latter group involves genes (*JmjC-SET and TPR-SET*) originating for the subphylum Pezizomycetes-specific roles and genes apparently connected with the occurrence of multicellularity; descendants of these genes are found also in animal and plant genomes (*E(z), SET2(ASH1*), and *G9a*). There is no Ascomycota-specific SET-domain family or gene (present in fungal genomes but absent from animals and plants), while there are families found exclusively in plant and animal genomes, as well as plant-specific or animal-specific subgroups. Animal and plant *SET-domain *genes are ancestrally related with complex fungal proteins with diverse modular elements. A combinatorial assembly of various peptide domains generates enormous possibilities for variation and precision required for functioning and adaptation of multicellular organisms.

## Methods

### Sequences used

The SET-domain sequences were searched from fourteen complete genomes listed in Additional file 1. Twenty-four SET-domain sequences used as queries were collected from various sources, as listed in Additional file 6.

### SET-domain protein mining

Four search methods were used to mine new SET-domain proteins from the fourteen genomes: BLAST protein similarity searches were conducted by BLASTP [96] using the 24 SET-domain sequences as the queries against the non-redundant database available at National Center for Biotechnology Information (NCBI) with the default settings. To find similar protein regions from unannotated genomic regions, TBLASTN [96] was used to perform similarity searches against nucleotide sequences of the fourteen genomes translated in all six frames. More sensitive searches were performed using the position specific iteration BLAST (PSI-BLAST) [97]. Each query was used against individual genomes with the inclusion E-value threshold of 0.001 and four search iterations.

#### Profile hidden Markov model searches

Profile hidden Markov models (HMMs), probabilistic models of multiple sequence alignments, were built and used to search for sequences with remote similarities [98]. Using the sequences obtained from the BLAST searches and the query sequences, we selected 27 well-aligned sequences (see Additional file 7). A profile HMM was built using these sequences with the Sequence Alignment and Modeling System (SAM) [99,100]. Two programs of the SAM package were used: *buildmodel *for building the profile HMMs and *hmmscore *(with *-sw 2 *and-*calibrate 1 *options) for searching similar protein sequences from the genomes. The searches were conducted in each genome individually. The resulting hits from each organism were analyzed for the presence of the SET-domain and previously unidentified sequences were collected. We did not use a strict E-value threshold. Rather each hit within the default E-value threshold (10) was examined one by one for the existence of the SET-domain.

After these similarity searches, 214 non-redundant hits were compiled from the twelve genomes (data not shown). Each of these 214 sequences was examined to confirm the presence of the SET domain by searching the Conserved Domain Database (CDD) available from NCBI [101], as well as the Simple Modular Architecture Research Tool (SMART) database [102,103]. Some dubious hits including too highly diverged sequences and those with very short SET-domain-like sequences were removed. Three fungal SET-MYND sequences (EAA36113, XP_381344, and XP_360530; see Additional file 2) were further used to search more SET-MYND sequences from fungal genomes using BLAST. After these analyses, we obtained 182 non-redundant SET-domain sequences. These sequences were used in our further analyses.

### Multiple alignments of SET-domain sequences

CLUSTALX (version 1.83) [104] was used to generate multiple alignments of SET-domain sequences (with the GONNET series protein weight matrices and gap opening penalty = 10 and gap extension penalty = .20). Due to the highly variable length of the SET-I region, poorly conserved sites across the sequences were removed. Some other highly variable positions were also removed and the alignments were adjusted manually (see Additional file 8).

### Phylogenetic analyses

A draft phylogeny was reconstructed using all of the 182 SET-domain sequences found in this study using the maximum likelihood method implemented in PHYML (version 2.4.4) [105]. This draft phylogeny (see Additional file 2) was used in the further analyses. Protein domain architectures for each protein group are shown in Additional file 3. In order to produce a more reliable multiple alignment and phylogenies, we reduced the number of sequences by choosing representative SET-domain sequences using the draft phylogeny as a guide tree. Poorly aligned sequences and those not clustering clearly with any known major SET-domain families were removed. All fungal sequences were retained, while only one each representative hit from plants and animals was chosen from each SET-domain cluster of SET1, SET2, Su(var)3-9, and E(z). The selected representative sequences are indicated in the phylogeny in red in Additional file 2. The final multiple alignment including 113 sequences is shown in Additional file 5. Phylogenetic reconstruction was done using the maximum likelihood method (implemented in PHYML version 2.4.4) and the maximum parsimony method (implemented in PHYLIP version 3.65). The neighbor-joining method was not used because the estimated distance matrix using the JTT substitution model (implemented in PHYLIP 3.65 [106]) generated estimation errors due to too many substitutions. For the maximum likelihood method, two sets of trees were reconstructed: one with no invariable site and a constant substitution rate among sites, and the other with the proportion of invariable sites and the gamma shape parameter estimated from the data. Reconstructed phylogenies were largely consistent. In our further analysis, we used the maximum likelihood phylogeny using the estimated proportion of invariable sites and gamma shape parameter. For the maximum parsimony method, the input sequence order was jumbled 10 times (see Additional file 9). Phylogenetic confidence was estimated by the bootstrap analysis [107] with 500 pseudoreplicates for all phylogenetic analysis.

## Authors' contributions

CSV collected data, carried out all of the bioinformatics analyses, and drafted the manuscript. ZA conceived of the study, contributed the discussion, and revised the manuscript. ENM conceived of the study, supervised the entire process of the study, and revised the manuscript. All authors read and approved the final manuscript.

## Supplementary Material

Additional file 1 Fourteen genomes used in this study. Click here for file

Additional file 2 The draft phylogeny including all 182 SET-domain sequences found in this study. Click here for file

Additional file 3 Gene architecture of SET-domain protein families. Click here for file

Additional file 4 SET3 and SET4 multiple sequence alignment. Click here for file

Additional file 5 The multiple sequence alignment of 113 representative SET-domain sequences. Click here for file

Additional file 6 SET-domain query sequences used to search new SET-domain proteins. Click here for file

Additional file 7 SET-domain proteins used for building the profile hidden Markov models. Click here for file

Additional file 8 The multiple sequence alignment of all 182 non-redundant SET-domain sequences. Click here for file

Additional file 9 The maximum parsimony tree reconstructed from the 113 representative SET-domain sequences. Click here for file
