# Supplementary material for: Evolution of SET-domain protein families in the unicellular and multicellular Ascomycota fungi
Source: BMC Evol Biol. 2008 Jul 1;8:190. doi: 10.1186/1471-2148-8-190 (PMC2474616; doi:10.1186/1471-2148-8-190)
Supplement: Additional file 1 — Fourteen genomes used in this study. [file 1471-2148-8-190-S1.pdf]

Fourteen genomes used in this study.

Species[a]	Genome size/ gene number	Sources and accession numbers
**Fungi** [Saccharomycotina (hemiascomycetes)]		
Yarrowia lipolytica (Yl)	20.5 Mb/ 6,700	European Molecular Biology Laboratory [http://www.ebi.ac.uk/genomes/]
Candida albicans (Ca)	15.6 Mb/ 6,090	CandidaDB [http://genolist.pasteur.fr/CandidaDB/]
Debaryomyces hansenii (Dh)	12 Mb/ 7,000	European Molecular Biology Laboratory [http://www.ebi.ac.uk/genomes/]
Ashbya gossypii[b] (Ag)	9.2 Mb/ 4,718	NCBI[c]: NC005782, NC005788 (Chromosomes 1-7)
Candida glabrata (Cg)	12.3 Mb/ 5,283	NCBI[c]: NC005967, NC005968, NC006026, NP006037 (Chromosomes A-M)
Saccharomyces cerevisiae (Sc)	12.1 Mb/6,294	*Saccharomyces* Genome Database [http://www.yeastgenome.org/]
**Fungi** [Pezizomycotina (euascomycetes)]		
Aspergillus fumigatus (Af)	29.4 Mb/ 9,926	Sanger Institute [http://www.sanger.ac.uk/Projects/Fungi/]
Neurospora crassa (Nc)	40 Mb/ 10,082	Fungal Genome Initiative [http://www.broad.mit.edu/annotation/]
Fusarium graminearum[b] (Fg)	40 Mb/ 11,640	Fungal Genome Initiative [http://www.broad.mit.edu/annotation/]
Magnaporthe grisea (Mg)	37.8 Mb/ 11,109	Fungal Genome Initiative [http://www.broad.mit.edu/annotation/]
**Fungi** [Taphrinomycotina (archeascomycetes)]		
Schizosaccharomyces pombe (Sp)	14 Mb/ 4,824	NCBI[c]: NC003424, NC003423, NC003421 (Chromosomes 1-3)
**Plant**		
Arabidopsis thaliana (At)	120 Mb/ 25,498	MIPS Arabidopsis genome database [http://mips.gsf.de/]
**Animals**		
Mus musculus (Mm)	2.5 Gb/ 24,174	NCBI[c]
Drosophila melanogaster (Dm)	165 Mb/ 13,600	FlyBase [http://flybase.bio.indiana.edu/]

[a]Species abbreviations used in this study are shown in parentheses.
[b]*Fusarium graminearum* is known also as *Gibberella zeae. Ashbya gossypii* is known also as *Eremothecium gossypii.*
[c]National Center for Biotechnology Information [http://www.ncbi.nlm.nih.gov/]
