# Supplementary material for: Evolution of SET-domain protein families in the unicellular and multicellular Ascomycota fungi
Source: BMC Evol Biol. 2008 Jul 1;8:190. doi: 10.1186/1471-2148-8-190 (PMC2474616; doi:10.1186/1471-2148-8-190)
Supplement: Additional file 2 — The draft phylogeny including all 182 SET-domain sequences found in this study. [file 1471-2148-8-190-S2.pdf]

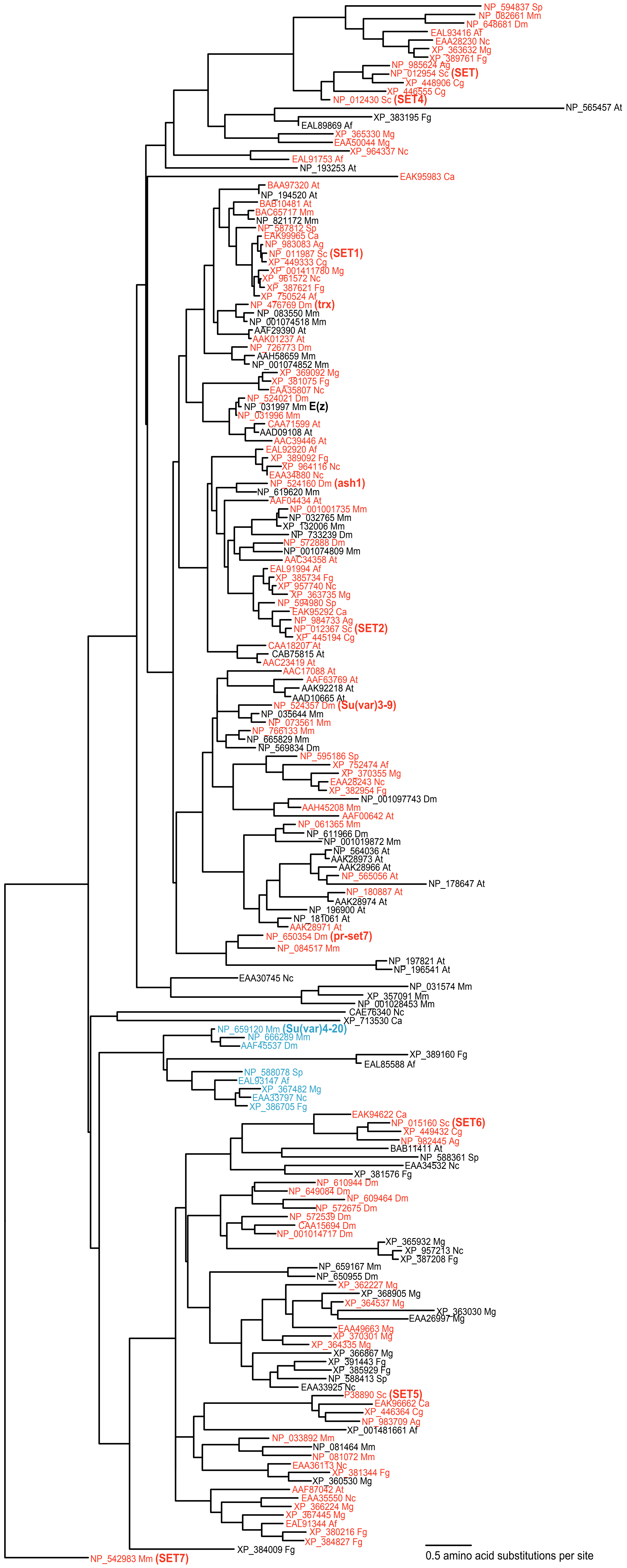

Maximum likelihood phylogeny reconstructed using 182 non-redundant SET-domain sequences.
Red font indicates 113 representative sequences used to reconstruct the phylogeny shown in Fig. 1.
