# Supplementary material for: Evolution of SET-domain protein families in the unicellular and multicellular Ascomycota fungi
Source: BMC Evol Biol. 2008 Jul 1;8:190. doi: 10.1186/1471-2148-8-190 (PMC2474616; doi:10.1186/1471-2148-8-190)
Supplement: Additional file 3 — Gene architecture of SET-domain protein families. [file 1471-2148-8-190-S3.pdf]

Domain architectures of SET-domain protein families

Symbols	Domain names
SET	SET domain
SET	SET domain (conservation only in C terminal region)
SET	SET domain (conservation only in N terminal region)
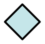	PHD Zn-finger
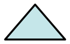	Zn-finger MYND
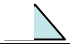	Zn-finger MYND (conservation only in C terminal region)
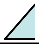	Zn-finger MYND (conservation only in N terminal region)
TUDOR	Tudor domain
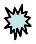	MORN (Membrane Occupation and Recognition Nexus)
IPT	IPT (Immunoglobulin-like fold)
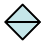	ANK (ankyrin repeats)
TPR	TPR (Tetratricopeptide repeat domain)
TPR	TPR (conservation only in C terminal region)
DUF	DUF (Domain of Unknown Function)
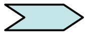	SANT ('SWI3, ADA2, N-CoR and TFIIIB' DNA-binding domains)
JMJC	JMJC (conservation only in C terminal region)
JMJC	JMJC
BAH	BAH (Bromo Adjacent Homology domain)
SRI	SRI (Set2 Rpb1 interacting domain)
AWS	AWS (associated with SET domains)
HMG	HMG (High Mobility Group-box)

Symbols	Domain names
PRS	PRS (pre-SET domain)
PRS	PRS (conservation only in C terminal region)
PRS	PRS (conservation only in N terminal region)
HMT M	HMT MBD (Histone Methyltransferase Methyl-CpG binding domain)
SRA	SRA (SET and RING finger associated domain)
GTPe	GTP elongation factor
RRM	RRM (RNA recognition motif)
UUP	UUP (ABC transporters ATPase domains)
BROMO	BROMO
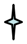	Post SET (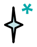 Diverged form of the post SET motif: CXCXXXXC)
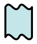	WW
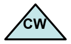	CW-type Zn-finger
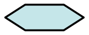	PWWP motif
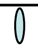	AT Hook
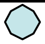	CHROMO
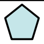	Zn-finger
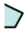	Zn-finger (conservation only in C terminal region)
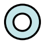	RING-finger
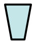	FYRN (F/Y-rich N-terminus)
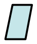	FYRC (F/Y-rich C-terminus)
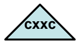	Zn-finger CXXC

In the following figures, each protein is represented by a solid horizontal line proportional to the length. Positions of domains are illustrated using the symbols above. The sizes of these symbols are not proportional to their actual size. When some regions are abbreviated for too long proteins, such regions are indicated with dashed lines with start and end position numbers as: 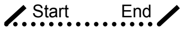

[SET 1 family]

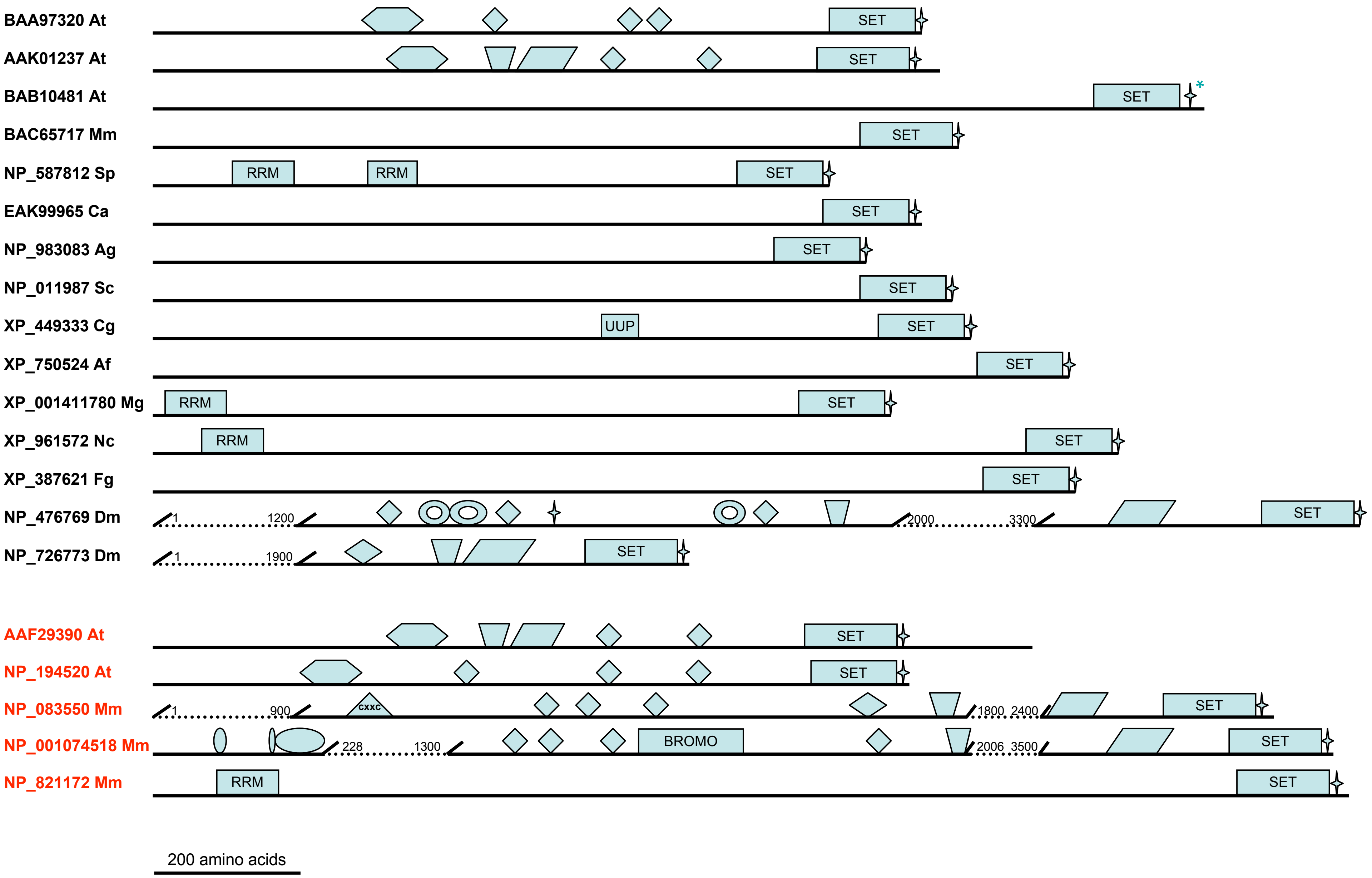

(NOTE: Sequences included only in the draft phylogeny, Additional file 2, are shown in red font.)

[SET 2 family]

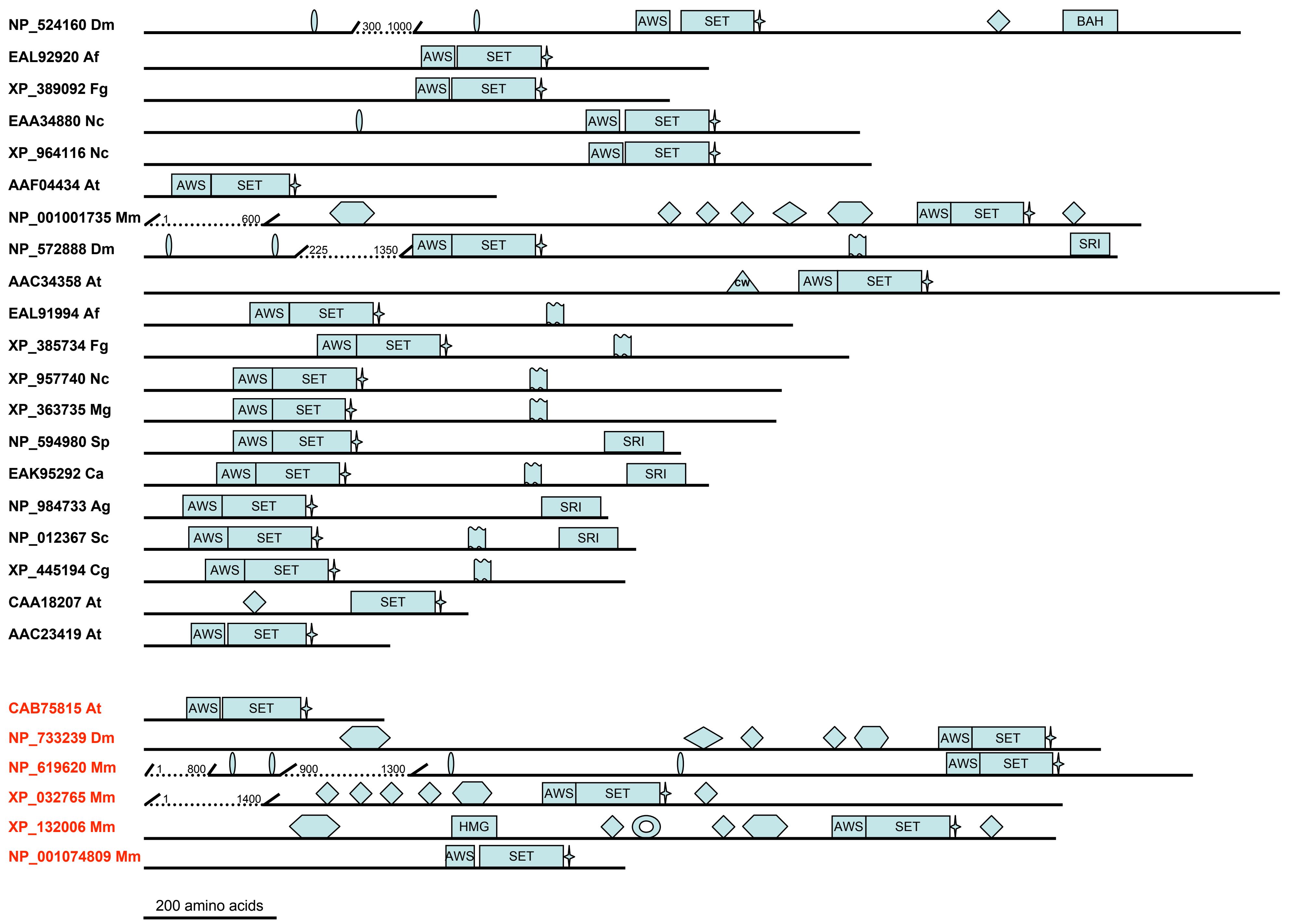
NP_524160 Dm
EAL92920 Af
XP_389092 Fg
EAA34880 Nc
XP_964116 Nc
AAF04434 At
NP_001001735 Mm
NP_572888 Dm
AAC34358 At
EAL91994 Af
XP_385734 Fg
XP_957740 Nc
XP_363735 Mg
NP_594980 Sp
EAK95292 Ca
NP_984733 Ag
NP_012367 Sc
XP_445194 Cg
CAA18207 At
AAC23419 At
CAB75815 At
NP_733239 Dm
NP_619620 Mm
XP_032765 Mm
XP_132006 Mm
NP_001074809 Mm
AWS
SET
BAH
SRI
HMG
CW
300
1000
1
600
225
1350
800
900
1300
1400
200 amino acids

[Su(var)3-9 family]

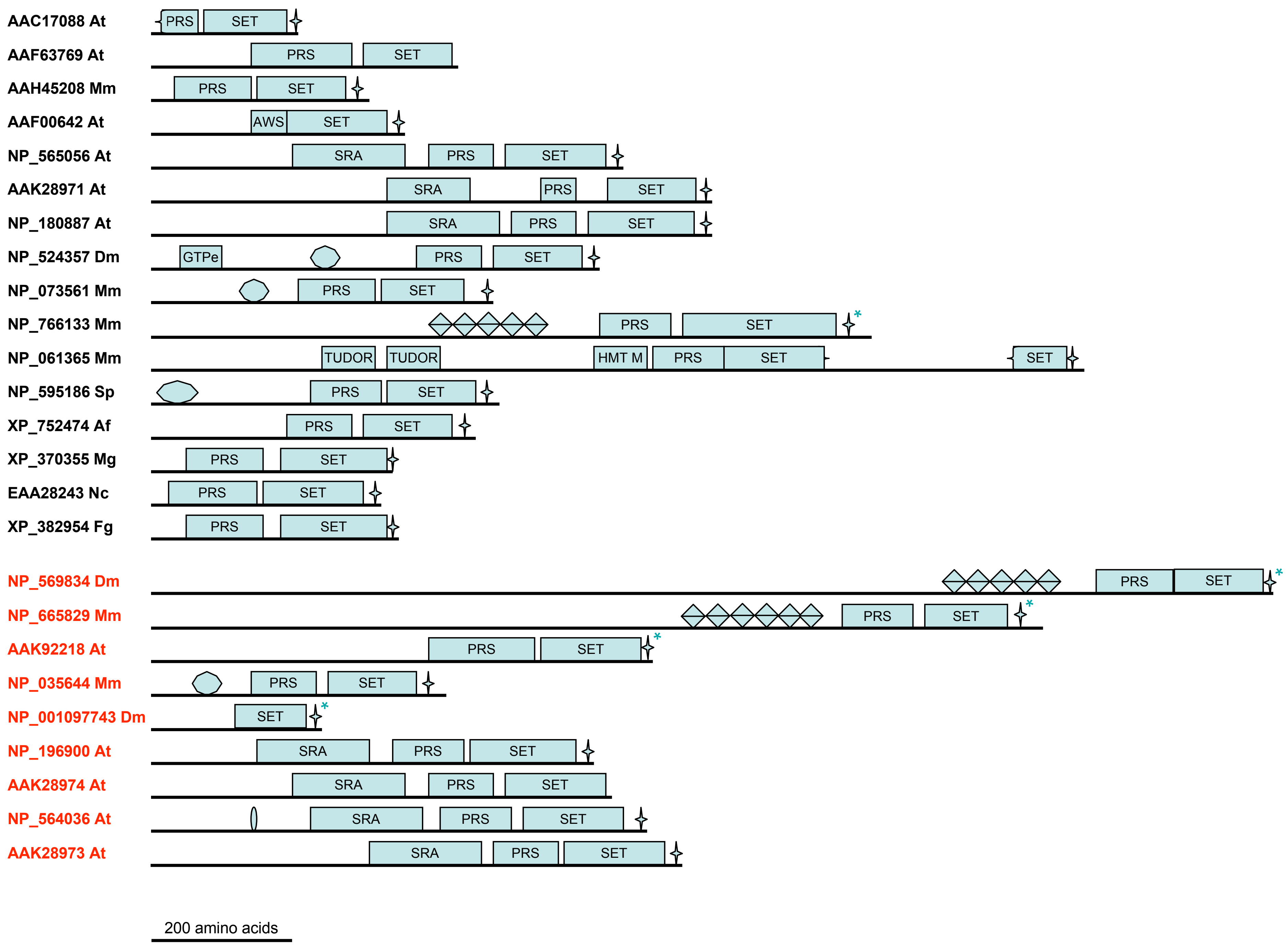

[SET 3/4 family]

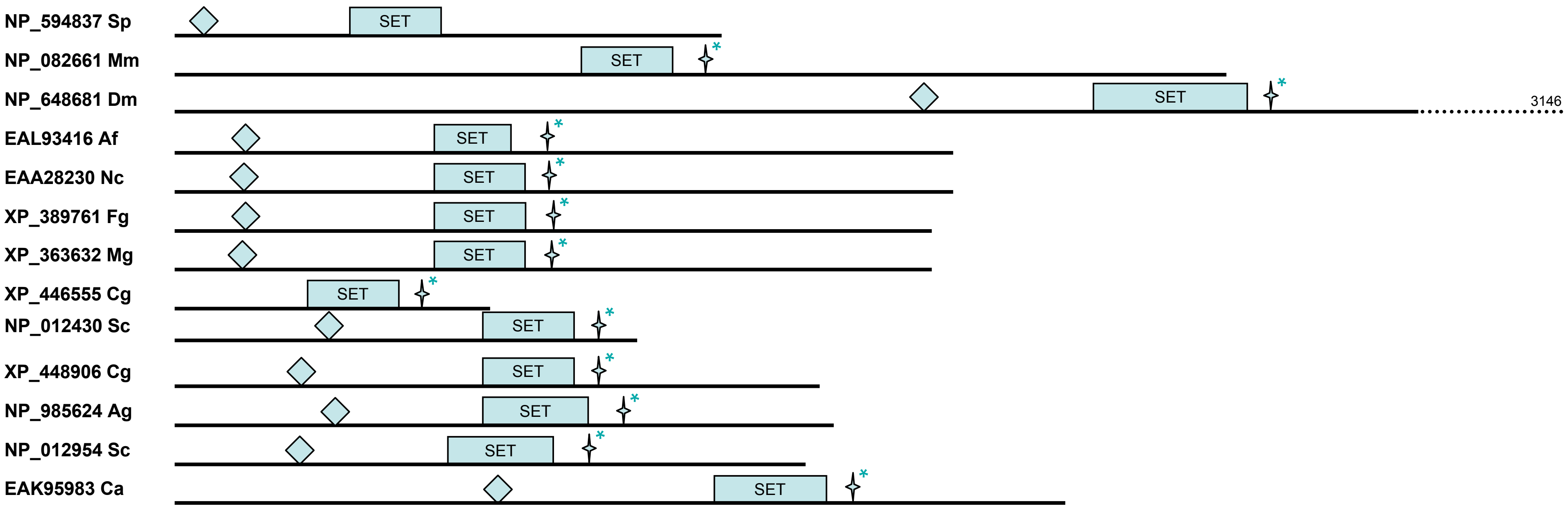

[SET JmjC family]

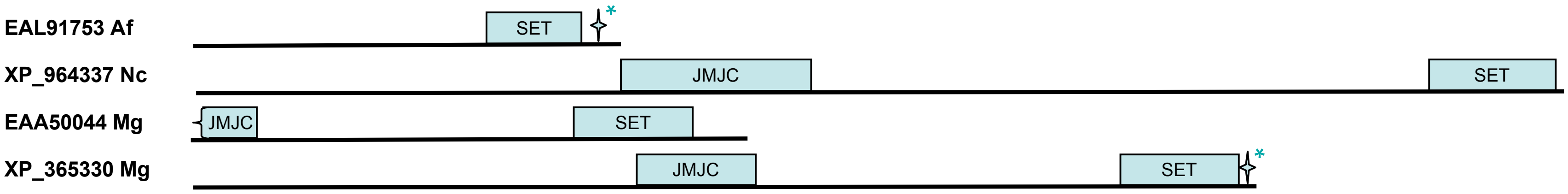

[SET 8 family]

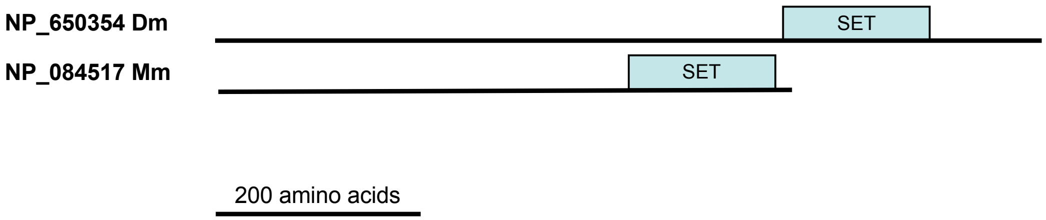

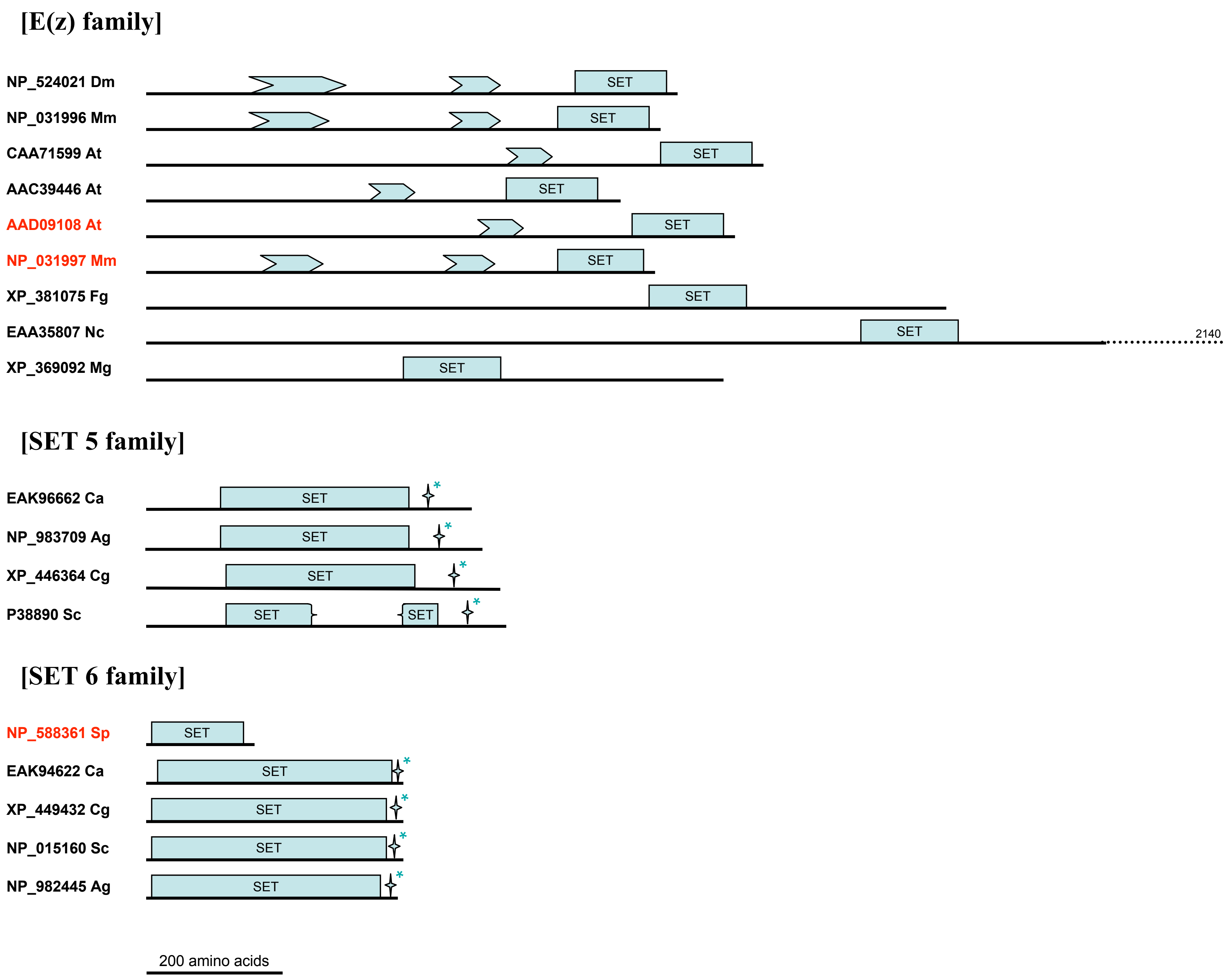
[E(z) family]
NP_524021 Dm
SET
NP_031996 Mm
SET
CAA71599 At
SET
AAC39446 At
SET
AAD09108 At
SET
NP_031997 Mm
SET
XP_381075 Fg
SET
EAA35807 Nc
SET
2140
XP_369092 Mg
SET
[SET 5 family]
EAK96662 Ca
SET
*
NP_983709 Ag
SET
*
XP_446364 Cg
SET
*
P38890 Sc
SET
SET
*
[SET 6 family]
NP_588361 Sp
SET
EAK94622 Ca
SET
*
XP_449432 Cg
SET
*
NP_015160 Sc
SET
*
NP_982445 Ag
SET
*
200 amino acids

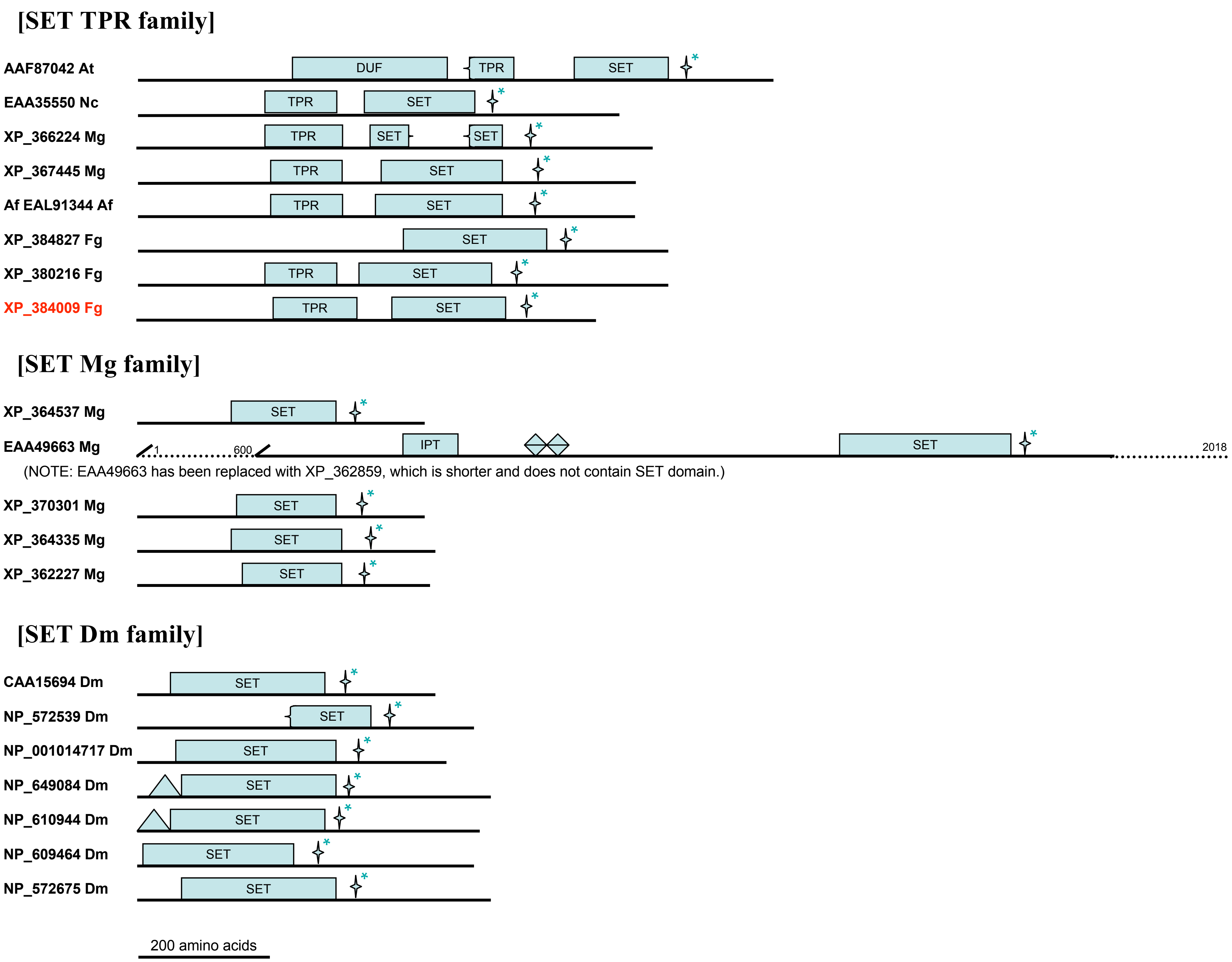
[SET TPR family]
AAF87042 At
DUF
TPR
SET
EAA35550 Nc
TPR
SET
XP_366224 Mg
TPR
SET
SET
XP_367445 Mg
TPR
SET
Af EAL91344 Af
TPR
SET
XP_384827 Fg
SET
XP_380216 Fg
TPR
SET
XP_384009 Fg
TPR
SET
[SET Mg family]
XP_364537 Mg
SET
EAA49663 Mg
1
600
IPT
SET
2018
(NOTE: EAA49663 has been replaced with XP_362859, which is shorter and does not contain SET domain.)
XP_370301 Mg
SET
XP_364335 Mg
SET
XP_362227 Mg
SET
[SET Dm family]
CAA15694 Dm
SET
NP_572539 Dm
SET
NP_001014717 Dm
SET
NP_649084 Dm
SET
NP_610944 Dm
SET
NP_609464 Dm
SET
NP_572675 Dm
SET
200 amino acids

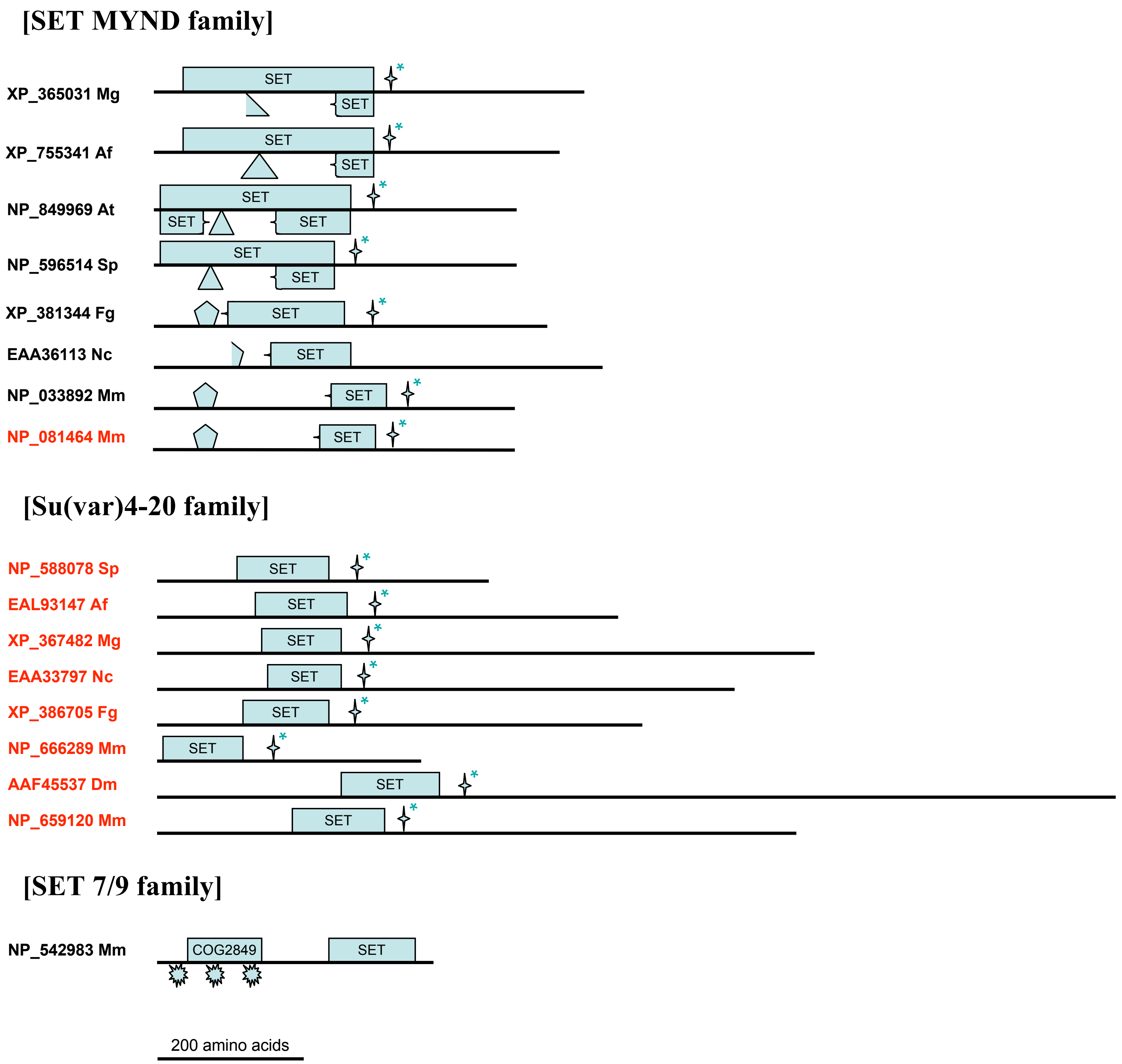
[SET MYND family]
XP_365031 Mg
SET
SET
XP_755341 Af
SET
SET
NP_849969 At
SET
SET
SET
NP_596514 Sp
SET
SET
XP_381344 Fg
SET
EAA36113 Nc
SET
NP_033892 Mm
SET
NP_081464 Mm
SET
[Su(var)4-20 family]
NP_588078 Sp
SET
EAL93147 Af
SET
XP_367482 Mg
SET
EAA33797 Nc
SET
XP_386705 Fg
SET
NP_666289 Mm
SET
AAF45537 Dm
SET
NP_659120 Mm
SET
[SET 7/9 family]
NP_542983 Mm
COG2849
SET
200 amino acids
