# Supplementary material for: Evolution of SET-domain protein families in the unicellular and multicellular Ascomycota fungi
Source: BMC Evol Biol. 2008 Jul 1;8:190. doi: 10.1186/1471-2148-8-190 (PMC2474616; doi:10.1186/1471-2148-8-190)
Supplement: Additional file 4 — SET3 and SET4 multiple sequence alignment. [file 1471-2148-8-190-S4.pdf]

**CLUSTAL X (1.83) multiple alignment of SET-domain sequences from the SET 3/4 family.** Species abbreviations: *Saccharomyces cerevisiae* (Sc), *S. bayanus* (Sb), *Schwanniomyces castelli* (Sca), *Candida glabrata* (Cg), *Ashbya gossypii* (*Ag*), *Candida albicans* (Ca), *Kluyveromyces lactis* (Kl), *Kluyveromyces waltii* (Kw), *Yarowia lioplytica* (Yl) and *Debaryomyces hansenii* (Dh).

```
NP_012430 Sc                         EVRKSSNERDFGVFAADSCVKGELIQEYLGKIDFQKNYQTDPNNDYRLMGTTKPKVLFHPHWPLYIDSRETGGLTRYIRRSCEPNVEL
AACA01000375.1(6257-6931) Sb         DIKKSSNGKDLGVFISNSCVKGQLIQN-IAKLTFKKITRQHASNNYRLMGTTQPKVLFHPHWPLFIDSREVGGLTRYIRRSCEPNV-L
XP_446555 Cg                         KPLASKDIQDFGVFTSIPCSKKDYIQEYSGMISFSKQYVNNAENKYDILGTPTRNILFHPHWPLYINTSGTKGAHEHLRCSCNPNVEL
AACF01000014.1(32239-32583) Sca      ------------------CLKGDLIDEFLGVVDFQKNYIMDPINQYRLWGTTKRDVIFHSQWPLLIDAREKSGRFRSLRRSCNPNVEL
NP_012954 Sc                         YSRTYPGFTKLGVYLKKDCIKGDFIQEILGELDFYKNYLTDPRNHYRIWGTAKRRVIFHSHWPIYIDARLSGNSTRYLRRSCQPNVEL
AACG02000197.1(8320-7895) Sb         YSRTYPGFTKLGVYLKRDCMKGDFVQEFLGELDFRKNYLTDSRNHYRIWGTPKRRVIFHPHWPIYIDARSSGNLTRYLRRSCQPNVEL
XP_448906 Cg                         YARTFPAHTKLGVFTKEYCNNGDLIEEFTGQVDFLKTYLDDTKNHYRIWGTAKNRVIFHPHWPLYIDARSKGNLTRFLRRGCKPNVEL
XP_455210 Kl                         HSRLFPGYPKLAVYLKQGCDEGDYIDEFLGEIDFQRKYLEDPRNNYRVWGTAKPKVIIHPHWPIYIDARLSGNLTRYLRRSCNPNVEL
NP_985624 Ag                         HSRVFHGFPKLGVYSQQPCAKDTLIAEFLGEVDFQRKYLEDPRNNYRLLGIPNPKVLFHPHWPIYIDARLCGNLTRYLRRSCYPNVEL
AADM01000081.1(81822-82199) Kw       HSRSFTGLSKMGLFVKQACPVGTYIEEVLGEVDFSKKYYADPRNNYRIFGTTKPKVFLHPHWPIYIDCRLSGNITRFMRRSCYPNVEL
AACF01000109.1(21368-21757) Sca      YSRTFPGFPKLGTFLPEGCNESALIQEFLGELNFKEDYLDDPRNMYRIWGTVKSKVVFHPNWPLCIDARSCGNLARYIRRCCNPNVGL
XP_504932 Yl                         PKSKFCGFSKHGLVANQPIQKDRFIIEYVGHVSHKDQYKADPINQYRIHPVPKSSVLFHPTLPLVIDGRLVGNDARFMRRSCNPNCRV
EAK95983 Ca                          -ALFISSNGGGSGGGSLTIPENTPIIEYLGEIDLFKNYCRDSINQYRMWGSPKPKVLKTTILDIVLDSRFVGNESRFIRKACSANCRI
NC_006044 Dh                         --------KDLKYEKEIIVPQETPIIEYLGEIDLFDNYVNDQVNQYSAFGTPKPKVLKVDLLEIVSDSRFVGNESRFIRKACTSNCKI

NP_012430 Sc                         VTVRPLDEKPRGDND-CRVKFVLRAIRDIRKGEEISVEWQWDLRNP
AACA01000375.1(6257-6931) Sb         ITVRPLNEKQRGESD-CRVTFVLMATRDIKVGEEISLKWQWDLRNP
XP_446555 Cg                         VTVRIMDNKPR-------IKFVIRAIRDIAEGEELQIAWQWDINHP
AACF01000014.1(32239-32583) Sca      VTVKLGQDQDKHWGD-STVKFMIRAKRTIQAGEELHINWQWDLRHP
NP_012954 Sc                         VTIKLQDTDNRNDKSSSRIKFVLRALRDISEDEELYIKWQWDSKHP
AACG02000197.1(8320-7895) Sb         VTIRLQDLDHENVNSVSKIKFVLRALRDISEDEELYIKWQWDLKQP
XP_448906 Cg                         VTVCLPSAGEEK-----DIKFILRATRDIDEGEELLIDWKWDLRHP
XP_455210 Kl                         VTIKLTNSNNQD-----EIKFVLRALRFIDKGEELHIKWDWDLRHP
NP_985624 Ag                         VTIKMPADDRQASDVNKSVKFVLKALRDLERGEELHIKWDWDLRHP
AADM01000081.1(81822-82199) Kw       VSMRMNDE--------SNVKFVLRALRDIDDDEELQIGWQWDLSHP
AACF01000109.1(21368-21757) Sca      STVKIKETN--------EIKFVLKALRDINPGEELHLSWHWDKKHP
XP_504932 Yl                         ATVVVNNTD---------IIFVVFATEPIKPGTELTLAWEWDTHHP
EAK95983 Ca                          ETVYVPEQNKFR---FLVFTSKPITLKSENQDEELRLPWEWDVDHP
NC_006044 Dh                         KPIYIPETNTFR---LLVVTSKPIILSSNTNEEELRLDWEWDRLHP
```
