# Supplementary material for: Evolution of SET-domain protein families in the unicellular and multicellular Ascomycota fungi
Source: BMC Evol Biol. 2008 Jul 1;8:190. doi: 10.1186/1471-2148-8-190 (PMC2474616; doi:10.1186/1471-2148-8-190)
Supplement: Additional file 6 — SET-domain query sequences used to search new SET-domain proteins. [file 1471-2148-8-190-S6.pdf]

SET-domain query sequences used to search new SET-domain proteins

Accession numbers	Species	References
**Su(var)3-9**		
NP_726483	*D. melanogaster*	Alvarez and Avramova (2002) *Gene* **285**:25-37
NP_524357	*D. melanogaster*	Alvarez and Avramova (2002) *Gene* **285**:25-37
NP_665829	*M. musculus*	Alvarez and Avramova (2002) *Gene* **285**:25-37
AAK28966	*A. thaliana*	Baumbusch *et. al.* (2001) *Nucleic Acids Res*. **29**: 4319-4333
AAD10665	*A. thaliana*	Baumbusch *et. al.* (2001) *Nucleic Acids Res*. **29**: 4319-4333
**E(z)**		
AAC39446	*A. thaliana*	Baumbusch *et. al.* (2001) *Nucleic Acids Res*. **29**: 4319-4333
AAC27124	*C. elegans*	Alvarez and Avramova (2002) *Gene* **285**:25-37
NP_031997	*M. musculus*	Alvarez and Avramova (2002) *Gene* **285**:25-37
**Trithorax**		
NP_011987	*S. cerevisiae*	Pijnappel *et. al.* (2001) *Genes Dev*. **15**:2991-3004
AAF29390	*A. thaliana*	Baumbusch *et. al.* (2001) *Nucleic Acids Res*. **29**: 4319-4333
BAA97320	*A. thaliana*	Baumbusch *et. al.* (2001) *Nucleic Acids Res*. **29**: 4319-4333
BAB10481	*A. thaliana*	Baumbusch *et. al.* (2001) *Nucleic Acids Res*. **29**: 4319-4333
NP_587812	*S. pombe*	Alvarez and Avramova (2002) *Gene* **285**:25-37
XP_11067	*M. musculus*	Alvarez and Avramova (2002) *Gene* **285**:25-37
**SET2**		
NP_012367	*S. cerevisiae*	Pijnappel *et. al.* (2001) *Genes Dev*. **15**:2991-3004
AAC23419	*A. thaliana*	Baumbusch *et. al.* (2001) *Nucleic Acids Res*. **29**: 4319-4333
CAA18207	*A. thaliana*	Baumbusch *et. al.* (2001) *Nucleic Acids Res*. **29**: 4319-4333
NP_524160	*D. melanogaster*	Schotta *et. al.* (2002) *Genes Dev.* **18**: 1251-1262
**SET3**		
NP_012954	*S. cerevisiae*	Pijnappel *et. al.* (2001) *Genes Dev*. **15**:2991-3004
**SET4**		
NP_012430	*S. cerevisiae*	Pijnappel *et. al.* (2001) *Genes Dev*. **15**:2991-3004
**SET5**		
P38890	*S. cerevisiae*	Pijnappel *et. al.* (2001) *Genes Dev*. **15**:2991-3004
**SET6**		
NP_015160	*S. cerevisiae*	Pijnappel *et. al.* (2001) *Genes Dev*. **15**:2991-3004
**SET8**		
AAH50346	*H. sapiens*	Fang *et. al.* (2002) *Curr. Biol.***12:** 1086-1099
**SET7/9**		
Q8WTS6	*H. sapiens*	Nishioka *et. al.* (2002) *Genes Dev*. **16**:479-489
