# Supplementary material for: Evolution of SET-domain protein families in the unicellular and multicellular Ascomycota fungi
Source: BMC Evol Biol. 2008 Jul 1;8:190. doi: 10.1186/1471-2148-8-190 (PMC2474616; doi:10.1186/1471-2148-8-190)
Supplement: Additional file 7 — SET-domain proteins used for building the profile hidden Markov models. [file 1471-2148-8-190-S7.pdf]

SET-domain proteins used for building the profile hidden Markov model.

SET-domain family	Accession numbers[a]	Species
E(z)	NP_031996	*Mus musculus*
E(z)	AAC39446	*Arabidopsis thaliana*
E(z)	AAF00642	*Arabidopsis thaliana*
SET1	NP_587812	*Schizosaccharomyces pombe*
SET1	NP_011987	*Saccharomyces cerevisiae*
SET1	BAC65717	*Mus musculus*
SET1	CAB71104	*Arabidopsis thaliana*
SET1	NP_726773	*Drosophila melanogaster*
SET2	NP_032765	*Mus musculus*
SET2	NP_012367	*Saccharomyces cerevisiae*
SET2	CAA18207	*Arabidopsis thaliana*
SET2	NP_619620	*Mus musculus*
SET3	NP_012954	*Saccharomyces cerevisiae*
SET3/4	NP_594837	*Schizosaccharomyces pombe*
SET4	NP_012430	*Saccharomyces cerevisiae*
SET5	P38890	*Saccharomyces cerevisiae*
SET6	NP_015160	*Saccharomyces cerevisiae*
SET7/9	NP_542983	*Mus musculus*
SET8	NP_081464	*Mus musculus*
Su(var)3-9	NP_611966	*Drosophila melanogaster*
Su(var)3-9	AAK28966	*Arabidopsis thaliana*
Su(var)3-9	NP_524357	*Drosophila melanogaster*
Su(var)3-9	NP_595186	*Schizosaccharomyces pombe*
Su(var)3-9	EAA28243	*Neurospora crassa*
Unknown	EAK94622	*Candida albicans*
Unknown	EAA58228	*Aspergillus nidulans*
Unknown	XP_384009	*Fusarium graminearum*

[a]All accession numbers are from National Center for Biotechnology Information (NCBI; http://www.ncbi.nlm.nih.gov/).
