# Supplementary material for: Evolution of SET-domain protein families in the unicellular and multicellular Ascomycota fungi
Source: BMC Evol Biol. 2008 Jul 1;8:190. doi: 10.1186/1471-2148-8-190 (PMC2474616; doi:10.1186/1471-2148-8-190)
Supplement: Additional file 8 — The multiple sequence alignment of all 182 non-redundant SET-domain sequences. [file 1471-2148-8-190-S8.pdf]

CLUSTAL X (1.83) multiple alignment of all 182 non-redundant SET-domain hits.

```
XP_387621 Fg     -PVKFARSAIH--NWGLYA--MENIAKDDMIIEYVGEQVRQQISEIRENR------YLKSGIGS-------------SYLFRID-------------DNT
XP_961572 Nc     -PVKFARSAIH--NWGLYA--MENINKDDMIIEYVGEEVRQQIAELREAR------YLKSGIGS-------------SYLFRID-------------DNT
XP_750524 Af     -PVRFARSAIH--NWGLYA--EENISANDMIIEYVGEKVRQQVADMRERQ------YLKSGIGS-------------SYLFRID-------------ENT
XP_449333 Cg     -PVTFARSAIH--NWGLYA--LEPINAKEMVIEYVGERIRQPVAEMRERR------YIKNGIGS-------------SYLFRID-------------EHT
NP_983083 Ag     -PVTFARSAIH--NWGLYA--LEPISAKEMIIEYVGERIRQPVAEMREKR------YLKSGIGS-------------SYLFRVD-------------EST
NP_011987 Sc     -PVMFARSAIH--NWGLYA--LDSIAAKEMIIEYVGERIRQPVAEMREKR------YLKNGIGS-------------SYLFRVD-------------ENT
EAK99965 Ca      -PVTFARSAIH--NWGLYA--MEPIAAKEMIIEYVGERIRQQVAEHREKS------YLKTGIGS-------------SYLFRID-------------DNT
XP_001411780 Mg -PVKFERSAIH--NWGLYA--MEHIPKDDMIIEYVGEEVRPSVAQVREAR------YDRSGIGS-------------SYLFRID-------------EDA
BAC65717 Mm      ------KSHIH--DWGLFA--MEPIAADEMVIEYVGQNIRQVIADMREKR------YEDEGIGS-------------SYMFRVD-------------HDT
NP_821172 Mm     ------RSRIH--EWGLFA--MEPIAADEMVIEYVGQNIRQMVADMREKR------YVQEGIGS-------------SYLFRVD-------------HDT
NP_587812 Sp     -QLHFGPSRIH--TLGLFA--MENIDKNDMVIEYIGEIIRQRVADNREKN------YVREGIGD-------------SYLFRID-------------EDV
BAB10481 At      -HLRFQQSKIH--DWGLVA--LEPIEAEDFVIEYVGELIRSSISEIRERQ------YEKMGIGS-------------SYLFRLD-------------DGY
NP_001074518 Mm ------RSPIH--GRGLFC--KRNIDAGEMVIEYAGNVIRSIQTDKREKY------YDSKGIG--------------CYMFRID-------------DSE
NP_083550 Mm     ------RSAIH--GRGLFC--KRNIDAGEMVIEYSGIVIRSVLTDKREKF------YDGKGIG--------------CYMFRMD-------------DFD
NP_476769 Dm     ------RSHIH--GRGLYC--TKDIEAGEMVIEYAGELIRSTLTDKRERY------YDSRGIG--------------CYMFKID-------------DNL
NP_001074852 Mm ------RSRIQ--GLGLYA--ARDIEKHTMVIEYIGTIIRNEVANRKEKL------YESQNRG--------------VYMFRMD-------------NDH
AAH58659 Mm      ------RSRIQ--GLGLYA--AKDLEKHTMVIEYIGTIIRNEVANRREKI------YEEQNRG--------------IYMFRIN-------------NEH
NP_726773 Dm     --VYLARSKIQ--GLGLYA--ARDIEKHTMIIEYIGEVIRTEVSEIREKQ------YESKNRG--------------IYMFRLD-------------EDR
AAK01237 At      -RLAFGKSGIH--GFGIFA--KLPHRAGDMMIEYTGELVRPSIADKREQL-----IYNSM-VGAG------------TYMFRID-------------DER
AAF29390 At      -RLAFGKSGIH--GFGIFA--KLPHRAGDMVIEYTGELVRPPIADKREHL-----IYNSM-VGAG------------TYMFRID-------------NER
BAA97320 At      -RVCFGRSGIH--GWGLFA--RRNIQEGEMVLEYRGEQVRGIIADLREAR------YRR--EGKD------------CYLFKIS-------------EEV
NP_194520 At     -RVCFGRSGIH--GWGLFA--RRNIQEGEMVLEYRGEQVRGSIADLREAR------YRR--VGKD------------CYLFKIS-------------EEV
CAA71599 At      -RVLLGISDIS--GWGAFL--KNSVSKHEYLGEYTGELISHKE--ADKRG----KIYD-RENC--------------SFLFNLN-------------DQF
AAD09108 At      -RILLGKSDVA--GWGAFL--KNSVSKNEYLGEYTGELISHHE--ADKRG----KIYD-RANS--------------SFLFDLN-------------DQY
NP_031996 Mm     -------SDVA--GWGTFI--KESVQKNEFISEYCGELISQDE--ADRRG----KVYD-KYMS--------------SFLFNLN-------------NDF
NP_031997 Mm     -------SDVA--GWGIFI--KDPVQKNEFISEYCGEIISQDE--DDRRG----KVYD-KYMC--------------SFLFNLN-------------NDF
NP_524021 Dm     -HLLMAPSDIA--GWGIFL--KEGAQKNEFISEYCGEIISQDE--ADRRG----KVYD-KYMC--------------SFLFNLN-------------NDF
AAC39446 At      -KILIGKSDVH--GWGAFT--WDSLKKNEYLGEYTGELITHDE--ANERG----RIED-RIGS--------------SYLFTLN-------------DQL
XP_381075 Fg     -GLALGQSQLEGVGYGLFT--VEPIAQDDFIIEYVGELITHDEGVEARRG----DVFDEESNI--------------SYVFTLLEN-----------EGI
EAA35807 Nc      --VLLGKSQLEGCGYGLFT--AEDISQDEFVIEYTGELITHDEGVEARRG----EGFGSQGTS--------------SYLFTLLEH-----------EGI
XP_369092 Mg     -PLCLGESGIEGCGYGLFT--AVDIAADEFIIEYVGELIQHDEGVEARRG----NVFDEESNV--------------SYLFTLLED-----------DGI
XP_385734 Fg     --ANVSVIKTEKKGFGLRA--DSDLQPNDFVFEYIGEVINEPTFRRRMIQ------YDEEGI-------------KHFYFMSLN-------------KSE
XP_957740 Nc     --ADVSVIKTEKKGFGLRA--NTDLQVNDFIFEYIGEVINEPTFRSRMVK------YDKEGI-------------KHFYFMSLT-------------KSE
EAL91994 Af      --ANVAVIKTEKKGFGLRA--ETDLRPHQFIFEYVGEVINEAQFRRRMRQ------YDEEGI-------------KHFYFMSLS-------------RGE
XP_363735 Mg     --ANVSVIKTENKGYGLRA--DANLEPNDFVFEYIGEVIGEELFRSRLMK------YDTQRL-------------EHFYFMSLT-------------RTE
NP_594980 Sp     --AKVDVFLTEKKGFGLRA--DANLPKDTFVYEYIGEVIPEQKFRKRMRQ------YDSEGI-------------KHFYFMMLQ-------------KGE
XP_445194 Cg     --ANIAVFKTKMKGYGVRA--ESDIEINDFIYEYKGEVIEEEEFRDRLVD------YDQKKF-------------RHFYFMMLQ-------------SGE
NP_012367 Sc     --APIAIFKTKHKGYGVRA--EQDIEANQFIYEYKGEVIEEMEFRDRLID------YDQRHF-------------KHFYFMMLQ-------------NGE
NP_984733 Ag     --ADIAVFQTEKKGYGVRA--ERDIEANEFIYEYIGEVISEADFRDRMVD------YDMRGF-------------KHFYFMMLQ-------------AGE
EAK95292 Ca      --SKVKVIQTELKGYGLIA--EQDIEENQFIYEYIGEVIDEISFRQRMIE------YDLRHL-------------KHFYFMMLS-------------NDS
NP_001074809 Mm -------------GWGLRA--AKDLPSNTFVLEYCGEVLDHKEFKARVKE------YARNKN-------------IHYYFMALK-------------NDE
NP_572888 Dm     ---PCRVFRTEKKGCGITA--ELLIPPGEFIMEYVGEVIDSEEFERRQHL------YSKDRN-------------RHYYFMALR-------------GEA
AAC34358 At      -YVKFERFQSGKKGYGLRL--LEDVREGQFLIEYVGEVLDMQSYETRQKE------YAFKGQ-------------KHFYFMTLN-------------GNE
XP_132006 Mm     --PETKIIKTDGKGWGLVA--KRDIRKGEFVNEYVGELIDEEECMARIKY-------AHENDI------------THFYMLTID-------------KDR
NP_032765 Mm     -------------GWGLRT--KTDIKKGEFVNEYVGELIDEEECRARIRY-------AQEHDI------------TNFYMLTLD-------------KDR
NP_001001735 Mm -------------GWGLRT--KRSIKKGEFVNEYVGELIDEEECRLRIKR-------AHENSV------------TNFYMLTVT-------------KDR
NP_733239 Dm     --------------FGLVN--REPIAVGDFVIEYVGEVINHAEFQRRMEQ------KQRDRD-------------ENYYFLGVE-------------KDF
AAC23419 At      -VKKMKLIQTEKCGSGIVA--EEEIEAGEFIIEYVGEVIDDKTCEERLWK------MKHRGE-------------TNFYLCEIT-------------RDM
CAB75815 At      -IKKMKLVQTEKCGYGIVA--DEDINSGEFIIEYVGEVIDDKICEERLWK------LNHKVE-------------TNFYLCQIN-------------WNM
CAA18207 At      -EKKIKIVKTEHCGWGVEA--AESINKEDFIVEYIGEVISDAQCEQRLWD------MKHKGM-------------KDFYMCEIQ-------------KDF
AAF04434 At      -YAKTKLIKCEGRGWGLVA--LEEIKAGQFIMEYCGEVISWKEAKKRAQT------YETHGV-------------KDAYIISLN-------------ASE
XP_389092 Fg     --VGVEVIKTSDRGYGVRS--NRCFRPNQIIMEYAGEIITEEECERRMTE-----VYKDNE---------------CYYLMSFD-------------QNM
EAA34880 Nc      -RIGVEVFKTEDRGYGVRS--NRCFEPHQIIMEYTGEIITDEECERRMNE-----EYKNNEVCFPRDCSS-----SCYYLMSFD-------------QNM
XP_964116 Nc     ----FQMLTERTK----KG--GRYRIGVEIIMEYTGEIITDEECERRMNE-----EYKNNE---------------CYYLMSFD-------------QNM
EAL92920 Af      --IGVEVIKTADRGYGVRS--NRTFEPNQIIVEYTGEIITQAECEKRMRT-----IYKNNE---------------CYYLMYFD-------------QNM
NP_524160 Dm     ---------TADKGWGVRT--KLPIAKGTYILEYVGEVVTEKEFKQRMAS-----IYLNDT---------------HHYCLHLD-------------GGL
NP_619620 Mm     --------RAEEKGWGIRT--KEPLKAGQFIIEYLGEVVSEQEFRNRMIE-----QYHNHS---------------DHYCLNLD-------------SGM
NP_196541 At     --PPLVVVFDPLEGYTVEA--DGPIKDLTFIAEYTG---DVDYLKNREKD------DCDSIMT---------------LLLSEDPSK----------TVI
NP_197821 At     --PPLMVVFDPYEGFTVEA--DRFIKDWTIITEYVG---DVDYLSNREDDY-----DGDSMMT---------------LLHASDPSQ----------CVI
```

```
NP_084517 Mm     --EGMKIDLIDGKGRGVIA--TKQFSRGDFVVEYHG-LIEITDAKKREALY-----AQDPSTG--------------CYMYYFQYL-----------STY
NP_650354 Dm     ---------------GVVA--DRPFKRNEFVVEYVG-LISIGEAAEREKRY-----ALDENAG--------------CYMYYFKHK-----------SQY
XP_752474 Af     -VRLEIFHTGN-RGFGLRS--PDWIRAGQFIDCYLGEVITKQEADVRE------EVVTSQHGH--------------SYLFELDFFHD-------D--EI
NP_595186 Sp     -LPLEIFKTKE-KGWGVRS--LRFAPAGTFITCYLGEVITSAEAAKRD------KNYD-DDGI--------------TYLFDLDMFDD-------A--SE
XP_382954 Fg     -IPLEIFRTED-RGWGVRS--PVSIRKGQFVDRYLGEIITSTEADRRRS----QSAISQRKD---------------VYLFALDKFTD-------KGPSL
EAA28243 Nc      -VPLQIFRTKD-RGWGVKC--PVNIKRGQFVDRYLGEIITSEEADRRRA----ESTIARRKD---------------VYLFALDKFSD-------AGQPL
XP_370355 Mg     -LPLQIFRTDDGRGWGVRA--TVDIKCGQFVDTYIGEVITDSEAVERR-------KATRKKD---------------LYLFDLDKFWQ-------DDSRL
NP_073561 Mm     -------------GWGVKT--LVKIKRMSFVMEYVGEVITSEEAERRGQ------FYDNKGI---------------TYLFDLDY----------ESDEF
NP_035644 Mm     -------------GWGVRT--LEKIRKNSFVMEYVGEIITSEEAERRGQ------IYDRQGA---------------TYLFDLDY----------VEDVY
NP_524357 Dm     ----VLFKTANGSGWGVRA--ATALRKGEFVCEYIGEIITSDEANERGK------AYDDNGR---------------TYLFDLDYNT--------QDSEY
NP_766133 Mm     --------RTQDMGWGVRS--LQDIPLGTFVCEYVGELISDSEADVRE---------ED------------------SYLFDLDNK---------DGEVY
NP_665829 Mm     -------------GWGVRA--LQTIPQGTFICEYVGELISDAEADVRE---------DD------------------SYLFDLDNK---------DGEVY
NP_569834 Dm     --------------WGVRA--LANVPKGTFVGSYTGEILTAMEADRRT---------DD------------------SYYFDLDN-----------G--H
AAD10665 At      -NKLQVFFTPNGKGWGLRT--LEKLPKGAFICEYIGEILTIPELYQRS--------FEDKP----------------TLPVILDAHWE-------GDKAL
AAK92218 At      -NKLQVFFTPNGRGWGLRT--LEKLPKGAFVCELAGEILTIPELFQR---------ISDRP----------------TSPVILDAYWG-------DDKAL
AAF63769 At      -CQLQVYFTQEGKGWGLRT--LQDLPKGTFICEYIGEILTNTELYDRNV-----RSSSERH----------------TYPVTLDADWK-------DEEAL
AAC17088 At      --AKLEVFRTESKGWGLRA--CEHILRGTFVCEYIGEVLDQQEANKRRN------QYGNGDC---------------SYILDIDANIE-------EELDY
AAH45208 Mm      -------------GWGLRT--LEFIPKGRFVCEYAGEVLGFSEVQRH-L-----QTSHDSN-----------------YIIAVREHI-------QIMET
NP_001097743 Dm  -KHLEIFDSPVYGSKGLRT--TAKITKGGYICEYAGELLTVPEARSHDN-----EKLGLMN-----------------YILVLNEYT-------KQQVT
AAF00642 At      -VSLKIVRD-EKKGWCLYA--DQLIKQGQFICEYAGELLTTDEARRNIY-----DKLRSTQSF--------------ASALLVVREHL-------ACLRI
NP_611966 Dm     --MKLQVFKTSNRGWGLRC--VNDIPKGAFICIYAGHLLTETMANEG-----GQDAGDEYFADL-------------DYIENSKD-----------EAPY
NP_061365 Mm     --VRLQLFKTQNKGWGIRC--LDDIAKGSFVCIYAGKILTDDFADKE-----GLEMGDEYFANL-------------DHIEKSTHGIG-------EESCY
NP_001019872 Mm  --VRLQVFKSEKKGWGVRC--LDDIDKGTFVCIYSGRLLRRATPEKTNIGE-GREQQHNSFSKK-------------RKLEASQSQQS-------KESLF
AAK28971 At      -KLPLEIFKTKSRGWGVRC--LKSIPIGSFICEYVGELLEDSEAERR-------IGNDEYLFDI------------GNRYESSG---------------F
NP_181061 At     --IKLEIFKTESRGWGVRS--LESIPIGSFICEYAGELLEDKQAESL-------TGKDEYLFDL------------G---EDDP---------------F
NP_196900 At     --FNLEVFRSAKKGWAVRS--WEYIPAGSPVCEYIGVVRRTADVDTI-------SDN-EYIFEI------------DCQQENAPE--------------F
AAK28974 At      -RNRLEVFRSLETGWGVRS--LDVLHAGAFICEYAGVALTREQANIL-------TMNGDTLVYP-------------ARFSIPPVD-------------F
NP_180887 At     --NRLEVFRSKETGWGVRT--LDLIEAGAFICEYAGVVVTRLQAEIL-------SMNGDVMVYP-------------GRFTLPPLD-------------F
AAK28966 At      -KVRLEVFKTANRGWGLRS--WDAIRAGSFICIYVGEAKDKSKVQQT-------MANDDYTFDT-------------TNVYEIPL-------------PL
NP_565056 At     --SRLEVFKTRNRGWGLRS--WDSLRAGSFICEYAGEVKDNGNLRGN-------QEEDAYVFDT-------------SRVFNLPS-------------PL
AAK28973 At      -KLHLEVFKTSNCGWGLRS--WDPIRAGTFICEFTGVSKTKEEVE----------EDDDYLFDT-------------SRIYNLPT-------------QV
NP_564036 At     --LHLEVFKTRNCGWGLRS--WDPIRAGTFICEFAGLRKTKEEVE----------EDDDYLFDT-------------SKIYNLPT-------------QV
NP_178647 At     -RPGLILEDLSNGAENLKV--CEVKENGPALFRYVTVINNIPSMVDR-------CACGRRSCGS-------------KHVFREKLS----------VSSL
EAA50044 Mg      ---GLYVSHYGKKGFGIR---VTFYGRRHVFGELVGNLVKPGSFSDGMG----------------------------LVFARDDLDEQ-------PVTAQ
XP_365330 Mg     ----PSVVYFDGKELGLQAM-AEAYRKGTLIGEIVGRIVPPNTYNDGRA----------------------------MNMVREDMDN--------EYVGQ
EAL89869 Af      ---VQIFSTASGKNNGVVA--RVNFDRGAAIGEFVGLITNGISGVD--------------------------------VMVGGTKSR----------TYQ
XP_383195 Fg     ---VQVFCTSNGRNNGLQA--LVAFERGTAIGEFVGLITKDIEEQD--------------------------------VMDSQAGGT----------RYQ
XP_357091 Mm     -CLMQTSFGDVP-HFGVFC--SDFIAKGVRFGPFRGRVVNASE-----------------------------------VKAKGS---------------
NP_031574 Mm     -LLFKYAANNSKEVIGVVS--KEYIPKGTRFGPLIGEVYTNDT-----------------------------------VPKNNEE--------------
NP_001028453 Mm  -CLCTSTVPGLA--YGICA--AQRIQQGTWIGPFQGVLLSPEK-----------------------------------VQTGVEP--------------
EAL91753 Af      ---VELVDYPQR-GIGVRA--LARFKKGDILDEYVGELRPLDYTDDRVYG--------------------------LLHESKMTEG----------HPL
XP_964337 Nc     -QIPRITQDGSR-GEGVRA--IGSFKADECLGELVGELRPPRTAGTSCSS--------------------------DHNHPNISSGQ----AQLLHDQLV
XP_449432 Cg     -YFDINETSW--GGRACFS--KRDLNNGQVVLEVEKAKFKGAGLWFC-----DTCRDKYLSIHDNTTTVL---LPDNLKNSLTVQAFRH---QD-EAVDS
NP_015160 Sc     -FFQVRQTKW--GGRACFS--NGNIPKGTTVLQVSPKKFLGAGLWFC-----EHCRTSYLQIPQSSESIL---LPSHLHRMLSIPLLRH---QE-EASDS
NP_982445 Ag     -LFEVKLTDY--GGRACFA--RQSQQRGDTVLKLTASSFGGGGLRFC-----EICKNAYMQMEEERENVV---LPIGLRDLLSSALFRK---EQ-EGNYS
EAK94622 Ca      -WFIVDETKY--GGRGCFA--PKVIPQ-HTVIHSCQHVYS---LFFC-----EECKSTFTHQ-DVKEALT---CTPELQAFIDSTTIRS---SEVDPSED
NP_983709 Ag     -KVQVTWS---EKGKGLVA--RRPLAKGELVFAED-ALSFIPP---------LEKSTLVHLSKACG-------MSMWEEGYGLFCDAFP---LRYVGKFN
P38890 Sc        -KVEVKFIDD-EHGRGLFA--KRDFSKGQIILKENKPIVYIPP---------LDKLFLISNGKACARSWR---ETVWEKCYELFCGAFP---LTMIGTFN
XP_446364 Cg     -KVEIRECEELRKGRGLYA--TRDIQQGELLFHEKVPIAMVPP---------MDKLKLIRSGKSCS-------GSLWKKAFDLFIRTFP---LEYLGRFH
EAK96662 Ca      -NVHIIMTS--KRGKGLYA--KRDIAKGDLIWSEE-PLFFIPP---------LANVNLMKTASACTYN-----EHLWKIGYEKFLRVFP---LFMMGTYN
XP_385929 Fg     -LFVVSDVPP--KGKGLIA--TTKIPKGTRIIAEPLLIKV---------------PRIIK---EECGEEV---GIAETNGLCAGP---------------
XP_391443 Fg     -MTELQEVPG--KGQGLIA--TRKIPKGTRILSEKAIIRV---------------PEIFANSDDDTSPYL---GIVRSNGL---P---------------
NP_588413 Sp     -IYKVVPIPN--KGMGMIA--KVKIPVGTRIFAETPLIRT---------------KSDAKEHPDTMGPFL---GPFYSNALTID----------------
EAA33925 Nc      -LYDIVPIPN--KGTGVIA--TQDIAKGTRIMAEKPLFIV---------------PTNVMEHGTTFSPFL---AICKTNMLGLGS---------------
EAA49663 Mg      -PYVAVPVPG--KGIGLRA--TQPIRTGHLVTARTPAVMVDER-------------TGLAD-AAKDH----------TNAYRTSIR--------------
XP_362227 Mg     -VYKSVMVPG--KGVGLVA--ARTLYRGDRIMSSTPAIVIDYG-------------ETMPDGLYENY----------TNAFDIDADPDD------------
XP_366867 Mg     IMYELRNSPG--KGKGLFA--SASVRQGDRIIAEAPILTVDLARFRL--------PPAELEGADENNPLS---SRFLTNALGTDEDDRQ---Q-----NP
XP_364335 Mg     -TYAVVPVPG--KGLGVVA--TRHIRAGEILMADDPLLVADVA--------------S--RG-SYSGG---------TNTFAVGVG--------------
XP_370301 Mg     -SWEVKKVPG--KGLGMVA--KRRIPRGETFLVDYAAILASVP--------------GRVRS-STQGA---------TNTFSLEID--------------
XP_368905 Mg     -LFERKEIPG--KGVGLVA--RRPISRGQLIMAQRPALVTDI-----------------KDISDQTDS-------ANTNSFNLNIAG-------------
XP_364537 Mg     -PFRAQAIPG--KGVGLVA--KRPIAKGELVMAETPAILVHK-----------------LEEATQGD--------LFTNSFQMALSGKA------------
EAA26997 Nc      KPFSESFILS--KGPGLTA--TIPLRRGKPLMSAAPVLLVHKD--------------FFVDIWRKSERNK---FLLLASPFEIDLGSNS---QA--TLQT
```

```
XP_363030 Mg      PPYFKADIPG--KGKGVIA--KRKIALGETIMILPPTLLVSDA--------------AYQAFGGARKALH---G--LDAAMKLDKNGFT---------YT
NP_572675 Dm      -PYRVEHSDIY--GRYLVA--NRQLEAGETLIREEPLAIGPCVSGDP------VCLGLEDEAEQ-VHEVC---GILDVNCFEIGQ-NGA-----------
NP_609464 Dm      -RTAVQWSPVC--GRYLVA--KGAIRGHGLLIEELPFAVGPKCNGPV------VCLGLANRSEELIMQVV---GVLEVNAFEARSPKGY-----------
NP_649084 Dm      -CFEIATNEVL--GRHLRA--TRDIKIGEQILKEAPLVLGPKVASAP------LCLGMKD-PEMDILRIA---AILDTNTFEVRQPRER-----------
NP_610944 Dm      -PFKIAHNEQL--GRHLVA--TRTIKPYEIVLKEAPLVRGPAQISAP------VCLGTQK-TEEEIMKAV---GALQINGHEVP-TTDP-----------
NP_001014717 Dm  -NWTISSSTVA--GRGVFA--TRDIAAGELIFQERALVTGPTARKGQ-----SSCICTTDRFMDQLFRIV---GVLNTNAFEAPC-RSG-----------
CAA15694 Dm       -SWRVADSPIS--GRGIFA--TREIAAGEELFREHTLLVGPTAHRSMN----RTCTLR-EQMLDYFYRTI---CAFNTNAFESRS-NVD-----------
NP_572539 Dm      -AWEIGVSKIA--GRGVVA--TRSLKRGEIIFRDSPLLIGLAAHEEDS----NACSVT-DKLIEIMNRTV---AVLRTNGFDKTTDRND-----------
NP_033892 Mm      -NVEVFTSEGK--GRGLKA--TKEFWAADVIFAERAYSAVVFD------------SLINCH----ISHIF---GVINCNGFTLSDQRLQ-----------
NP_081072 Mm      -GLERFCSAGK--GRGLRA--LRPFHVGDLLFSCPAYACVLTV------------GERGCE----LVVLF---AQVNCNGFTIEDEELS-----------
NP_081464 Mm      -KVEKFTTANR--GNGLRA--VAPLRPGELLFRSDPLAYTVCK------------GSRGCDPSFDLFEAF---AKVICNSFTICNAEMQ-----------
XP_360530 Mg      -AVHGTKGVRS--GRSLRA--TRDFQPGDLIAEFDNPLAAFP-------------RASTTCHHFLSLDVL---CKIQTNSFDR----FDAD---------
XP_381344 Fg      -APFRVKSHRK--GRGIFS--TKSFAPGDVILPFT-PTILIP-------------HINTICSHQKALTIL---CKIQTNAFHR----YDTD---------
EAA36113 Nc       -RIGGIPGVDR--GRSLFS--TERFGAGETIAIIENPLLALP-------------NMRTTCNYKQAMEVL---CKIQTNAFNR----FDAD---------
NP_650955 Dm      -NFEIRELPGK--GRAMIA--TKNFAKDEVIFEEEPFVSRQFSWNVAYG--YAKVRYCLTDSEKEQLDTV---IDGLYAKVGEF--EFLN----------
NP_659167 Mm      ----------------------------------------------------------LTPQDREQLDTF---IDQLYKDIEAA--EFLN----------
XP_384827 Fg      -PVEIRDSPGR--GRGLFT--TKSVSAGDLLLVEKAFSYSYMDENRLW-----DQITYMINLTSAFQDLV---DGG-LNSFG----SPT------SCRER
XP_380216 Fg      -PVEIRESPGR--GKALFT--TKAVSAGELLLCKKAFSYSFAGDEQST-----KQTKILMNLAAEFGDLH---HAD-LNGFG----APR------TGDEG
EAL91344 Af       -PVAVRPTESR--GRGLFT--TEAVKAGDLLFCEKAFAHAFHDADDPR-----RSIGLLINPQPAFIDLY---HDS-LNCFG----CPL------DHDEQ
XP_367445 Mg      -RTRIAKTDNR--GRGLFA--TVPLCEGDVIFVEKAFFTVHRDAG---------DLAVLININKRYYDLF---DGGKLNGFG----CPR-------VQEG
XP_366224 Mg      -RTEERPSATH--GRGLFA--TERIAAGELVFVEKATVMPPQYDE------------------RRLCRMH---AGDYRNCFS----APL-------GLAR
EAA35550 Nc       -NTRVADSGFH--GRGLFA--TRPLKAGDLVYVEKATLMPNQYDP------------------DTMLKLY---PGQLKNCFS----SPL-------TYN-
AAF87042 At       GSIEIKKSELS--GRGLFA--TKNIVAGTLVLVTKAVAIERGILGNGE-----CGEKAQLIMWPEIALFR---PDEAFETCGDWKQSLD------VMGKN
XP_387208 Fg      ---DYEIIPT-CKQLGVFA--KEDIAPGEAVLKEYSLLTANNRLKDSI-----CDACSPPKMDIDIYENL---DEYDLWVMN----GTA---------RD
XP_957213 Nc      ---EYEIIPT-CNQLGLFA--KEDIAPGEAVLKEYSLLTANNRHKEST-----CDACGPPKMDIDIFETL---PQHDLWVFN----GTA---------RD
XP_365932 Mg      ---GVAEQGT-CKQLGVFA--AADIGPGEVVLREFSALTANNRLQEPV-----CDACGPSTMDVDIYRTL---ARHDTWVMN----GTA---------RD
XP_367482 Mg      ----NRYTIFTH-EASVTA--RRPIRKNEIIKALCGIQVVISP---------------------------------AEEASK------------------
EAA33797 Nc       -NATNRYTIVTY-EASITA--RRFIQRNETIKYLAGIQVVITP---------------------------------EEELSK------------------
XP_386705 Fg      -SSTNRYTIVSH-EAAVTA--RRAIRRNEAIKYLSGVQVVITP---------------------------------EEEMSK------------------
EAL93147 Af       -TTTNRYTITEY-EAAVCA--RKFIKQGQEIKYLSGTLVPMTR---------------------------------EEERKK------------------
NP_588078 Sp      -CSTNQYFSSSKPEACVIA--RESINAGEDITDLCGTIIKLSP---------------------------------KEERLD------------------
AAF45537 Dm       --ACYRYTLEEQRGAKISS--TKRWSKNDKIECLVGCIAELTE--------------------------------AEEAAKN------------------
NP_659120 Mm      -LPCNRYSSE-QNGAKIVA--TKEWKRNDKIELLVGCIAELSE--------------------------------IEENMKN------------------
NP_666289 Mm      --------ME-TNGAKIVS--TRAWKKNEKLELLVGCIAELRE---------------------------------EDEDKR------------------
EAL85588 Af       LMFWSPSELRELQGSAIVS--KIGKEGALRLAHIMGSLIMAYA----------------FDIEKVEDEED---GVTDDEQDQS-----------------
XP_389160 Fg      PMFWSENELDQLQASHMRH--KIGKADAVEIAHRMGSTIMAYA----------------FDLENEETEE------VEDREGKS-----------------
XP_389761 Fg      ----------KALRWRFLKS-STPIEKDVPLVELNGAIGFQKDYCADPT--------NLWADLSSPL---------PFVFFHPVLP------------LY
XP_363632 Mg      --KQLSLARETVARWQYLMT-PTPVAKDVPLIELNGSIGFQKDYCADKH--------NRWDEFSSPL---------PFVFFHEMLP------------LY
EAA28230 Nc       --SRKTLGQGTVASWKCLKA-PSAIAHNVPLVELNGQIGIQSSYCADPD--------SRWQELTSPL---------PYVFFHPMLP------------IY
EAL93416 Af       ---PSINIEGQHPTWKVLRT-RESVSKDHIVGEITGKIGLLRDYCLDPS--------NRWQELRHPE---------PFVFFHPQLP------------IY
NP_648681 Dm      -----AQLIPYAG-AKVLIS-SVDLSPHAPIHELRGKYMLTTQFRTQNPTV------NSFKAHKTPG---------QFVFFYLKGPE-----------VC
NP_082661 Mm      ---QLGRVTRVQKHRKILRA-ARDLALDTLIIEYRGKVMLRQQFEVNG---------HFFKKPY------------PFVLFYFNGVE-----------MC
XP_448906 Cg      -ESSYARTFPAHTKLGVFT--KEYCNNGDLIEEFTGQVDFLKTYLDDTK--------NHYRIWGTAK---------NRVIFHPHWP------------LY
NP_012954 Sc      -DIAYSRTYPGFTKLGVYL--KKDCIKGDFIQEILGELDFYKNYLTDPR--------NHYRIWGTAK---------RRVIFHSHWP------------IY
NP_985624 Ag      -EPSHSRVFHGFPKLGVYS--QQPCAKDTLIAEFLGEVDFQRKYLEDPR--------NNYRLLGIPN---------PKVLFHPHWP------------IY
XP_446555 Cg      --VLKPLASKDIQDFGVFT--SIPCSKKDYIQEYSGMISFSKQYVNNAE--------NKYDILGTPT---------RNILFHPHWP------------LY
NP_012430 Sc      -ADIEVRKSSNERDFGVFA--ADSCVKGELIQEYLGKIDFQKNYQTDPN--------NDYRLMGTTK---------PKVLFHPHWP------------LY
EAA30745 Nc       -WLEIRHTGTAEKGYGVFVK-TNEIPKGAYLGHYVGEIIPGPTKGYNANN--------------------------KYLYEPQVG----------WDTD
XP_001481661 Af  -NKTQSKYIDESIGYGLFA--TGRIPAGEVVFADKVQAKTADDLVAERVRA---EEWHRGFLCLP----------NPQPAYAQIWYH---------MIAL
XP_384009 Fg      --GSTEVKSSPGAGRGLFT--TRDISPNEIIMCEKASLVCDTRPSQVERIL----LQGDYKGIG-------------QKLIELDEVPQQT-------EDE
BAB11411 At       --PPIRVGLTESAGRAVFA--TRKIGAGDLIHTAKPKLCLKKLFLDVETR------ADWSSFDDYFLTKQ---NAFRIDLVAAS-------------VE
NP_588361 Sp      ---PLEIRDTERKGRGVFA--LEPIPAQTCIEISPVLMFSKEEYEQHG-----------------------------QYTVTYVWS--------------
EAA34532 Nc       --NNFQVRLSRYGGFGTFA--TRDLKMGEVILNEKPLLRTPRDSFYTEFLK-----EEDQAKFMQLYTPH---DGSDYHIRGILKAN---------SFAI
XP_381576 Fg      --EYFQVRRSDLAGWGAFA--VRELKEGDQILLEKP-LFTATNLTLFDGFA--EVAYSLHANNN-----------VEVLIWKTNAFSTAN------PSKG
NP_193253 At      --RSDDKYVSYRKGLGVVC--NKEFGEEDFVVEFLGEVYPVWKWFKQDGIR----SLQENKTDPAP----------EFYNIYLERPK----------YDL
NP_594837 Sp      --VPISSSKFCCSRFGLVS--TCEIPPNTPIMEVKGRVCTQNEYKSDPKN-----QYNILGAPKP------------HVFFDSN--------------SQ
EAK95983 Ca       ----ALFISSNGGGSGGGS---LTIPENTPIIEYLGEIDLFKNYCRDSIN-----QYRMWGSP------------KPPTQYSDET-------------LD
XP_713530 Ca      -FDIGKRHFSDKLKLGIVSS-IKLIGHEIGLTLFKKNLIKVSKKNWKFID----DLVAIVFIN-------------YFTLFN-----------------Y
NP_542983 Mm      -RVYVADSLISSAGEGLFS--KVAVGPNTVMSFYNGVRITHQEVDSRDWAL------NTLSLD-------------EETVIDVP--------------EP
NP_565457 At      -RLLASSAHQKCQSCSLVS-FCSFASHTPWLCESLRRLHQSSSSAQPSDRQ---VFQILLSLQGSGSSNGDCSAGDSASLLSSVISPDLTG---MEPCSV
CAE76340 Nc       -----IEPDVTSSRKNLVHGRFAGITREEYETYVAGEMLIANPTEEDRQK----ACHDFFKHYAFQVKRPHLPSPITTNTLSPITSSSTAS------ASI
```

```
XP_387621 Fg     VIDATK-KGGIARFINHS--------------------FEGSK---RIVIYA-LRDIALNEELTYDYKFER
XP_961572 Nc     VIDATK-KGGIARFINHSCMP--N---CTAKIIK-----VEGSK---RIVIYA-LRDIAQNEELTYDYKFER
XP_750524 Af     VIDATK-RGGIARFINHSCTP--N---CTAKIIK-----VDGSK---RIVIYA-LRDIGRDEELTYDYKFER
XP_449333 Cg     VIDATK-KGGIARFINHCCEP--S---CTAKIIK-----VGGKR---RIVIYA-LRDIAANEELTYDYKFER
NP_983083 Ag     VIDATK-KGGIARFINHCCDP--S---CTAKIIK-----VGGMK---RIVIYA-LRDIAANEELTYDYKFER
NP_011987 Sc     VIDATK-KGGIARFINHCCDP--N---CTAKIIK-----VGGRR---RIVIYA-LRDIAASEELTYDYKFER
EAK99965 Ca      VIDATK-KGGIARFINHCCSP--S---CTAKIIK-----VEGKK---RIVIYA-LRDIEANEELTYDYKFER
XP_001411780 Mg  VIDATK-KGGIARFINHSCMP--N---CTAKIIR-----VEGTK---RIVIYA-LRDIARNEELTYDYKFEL
BAC65717 Mm      IIDATK-CGNFARFINHSCNP--N---CYAKVIT-----VESQK---KIVIYS-KQHINVNEEITYDYKFPI
NP_821172 Mm     IIDATK-CGNLARFINHCCTP--N---CYAKVIT-----IESQK---KIVIYS-KQPIGVDEEITYDYKFPL
NP_587812 Sp     IVDATK-KGNIARFINHSCAP--N---CIARIIR-----VEGKR---KIVIYA-DRDIMHGEELTYDYKFPE
BAB10481 At      VLDATK-RGGIARFINHSCEP--N---CYTKIIS-----VEGKK---KIFIYA-KRHIDAGEEISYNYKFPL
NP_001074518 Mm  VVDATM-HGNAARFINHSCEP--N---CYSRVIN-----IDGQK---HIVIFA-MRKIYRGEELTYDYKFPI
NP_083550 Mm     VVDATM-HGNAARFINHSCEP--N---CFSRVIH-----VEGQK---HIVIFA-LRRILRGEELTYDYKFPI
NP_476769 Dm     VVDATM-RGNAARFINHCCEP--N---CYSKVVD-----ILGHK---HIIIFA-LRRIVQGEELTYDYKFPF
NP_001074852 Mm  VIDATL-TGGPARYINHSCAP--N---CVAEVVT-----FERGH---KIIISS-NRRIQKGEELCYDYKFDF
AAH58659 Mm      VIDATL-TGGPA-----------------AEVVT-----FDKED---KIIIIS-SRRIPKGEELTYDYQFDF
NP_726773 Dm     VVDATL-SGGLARYINHSCNP--N---CVTEIVE-----VDRDV---RIIIFA-KRKIYRGEELSYDYKFDI
AAK01237 At      VIDATR-TGSIAHLINHSCVP--N---CYSRVIT-----VNGDE---HIIIFA-KRHIPKWEELTYDYRFFS
AAF29390 At      VIDATR-TGSIAHLINHSCEP--N---CYSRVIS-----VNGDE---HIIIFA-KRDVAKWEELTYDYRFFS
BAA97320 At      VVDATE-KGNIARLINHSCMP--N---CYARIMS-----VGDDES--RIVLIA-KTTVASCEELTYDYLFDP
NP_194520 At     VVDATD-KGNIARLINHSCTP--N---CYARIMS-----VGDEES--RIVLIA-KANVAVGEELTYDYLFDP
CAA71599 At      VLDAYR-KGDKLKFANHSPEP--N---CYAKVIM-----VAGDH---RVGIFA-KERILAGEELFYDYRYEP
AAD09108 At      VLDAQR-KGDKLKFANHSAKP--N---CYAKVMF-----VAGDH---RVGIFA-NERIEASEELFYDYRYGP
NP_031996 Mm     VVDATR-KGNKIRFANHSVNP--N---CYAKVVM-----VNGDH---RIGIFA-KRAIQAGEELFFDYRYSQ
NP_031997 Mm     VVDATR-KGNKIRFANHSVNP--N---CYAKVMM-----VNGDH---RIGIFA-KRAIQTGEELFFDYRYSQ
NP_524021 Dm     VVDATR-KGNKIRFANHSINP--N---CYAKVMM-----VTGDH---RIGIFA-KRAIQPGEELFFDYRY--
AAC39446 At      EIDARR-KGNEFKFLNHSARP--N---CYAKLMI-----VRGDQ---RIGLFA-ERAIEEGEELFFDYCYGP
XP_381075 Fg     WVDAAT-YGNLSRYINHASESDKN---ITPRILY-----VNGEY---RIKFTA-MRDIAAGEELFFNYGENF
EAA35807 Nc      WVDAAM-YGNLSRYINHASENDKN---ITPKIIY-----VNNEY---RIKFTA-LRDIKAGEELFFNYGDNF
XP_369092 Mg     WVDAAV-YGNLSRYMNHASESDRN---VVPKIVQ-----VNGDF---RIRFTA-LRDIKAGEELFFNYGENF
XP_385734 Fg     FVDATK-KGNYGRFCNHSCNP--N---CYVDKWV-----VGDKL---RMGIFT-SRKIQSGEELVFNYNVDR
XP_957740 Nc     FVDATK-KGNLGRFCNHSCDP--N---CYVDKWV-----VGDKL---RMGIFA-GRAIKAGEELVFNYNVDR
EAL91994 Af      FVDATK-KGNLGRFCNHSCNP--N---CYVDKWV-----VGEKL---RMGIFA-ERAIQAGEELVFNYNVDR
XP_363735 Mg     YVDATK-KGNLGRFCNHSCNP--N---CYVDKWV-----VGDKL---RMGIFA-MRAIKAGEELCFNYNVDR
NP_594980 Sp     YIDATK-RGSLARFCNHSCRP--N---CYVDKWM-----VGDKL---RMGIFC-KRDIIRGEELTFDYNVDR
XP_445194 Cg     FIDATI-KGSLARFCNHSCNP--N---AYVNKWV-----VAGKL---RMGIFA-KRKILKGEEITFDYNVDR
NP_012367 Sc     FIDATI-KGSLARFCNHSCSP--N---AYVNKWV-----VKDKL---RMGIFA-QRKILKGEEITFDYNVDR
NP_984733 Ag     FIDATE-RGCLARFCNHSCNP--N---AYVSKWD-----VAGKL---KMGIFA-HRKILKGEEITFDYNVDR
EAK95292 Ca      FIDATE-KGSLGRFINHSCNP--N---AFVDKWH-----VGDRL---RMGIFA-KRKISRGEEITFDYNVDR
NP_001074809 Mm  IIDATQ-KGNCSRFMNHSCEP--N---CETQKWT-----VNGQL---RVGFFT-TKLVPSGSELTFDYQF--
NP_572888 Dm     VIDATS-KGNISRYINHSCDP--N---AETQKWT-----VNGEL---RIGFFS-VKPIQPGEEITFDYQYLR
AAC34358 At      VIDAGA-KGNLGRFINHSCEP--N---CRTEKWM-----VNGEI---CVGIFS-MQDLKKGQELTFDYNYVR
XP_132006 Mm     IIDAGP-KGNYSRFMNHSCQP--N---CETLKWT-----VNGDT---RVGLFA-VCDIPAGTELTFNYNLDC
NP_032765 Mm     IIDAGP-KGNYARFMNHCCQP--N---CETQKWS-----VNGDT---RVGLFA-LSDIKAGTELTFNY----
NP_001001735 Mm  IIDAGP-KGNYSRFMNHSCNP--N---CETQKWT-----VNGDV---RVGLFA-LCDIPAGMELTFNY----
NP_733239 Dm     IIDAGP-KGNLARFMNHSCEP--N---CETQKWT-----VNCIH---RVGIFA-IKDIPVNSELTFNYLWD-
AAC23419 At      VIDATH-KGNKSRYINHSCNP--N---TQMQKWI-----IDGET---RIGIFA-TRGIKKGEHLTYDYQFVQ
CAB75815 At      VIDATH-KGNKSRYINHSCSP--N---TEMQKWI-----IDGET---RIGIFA-TRFINKGEQLTYDYQFVQ
CAA18207 At      TIDATF-KGNASRFLNHSCNP--N---CVLEKWQ-----VEGET---RVGVFA-ARQIEAGEPLTYDYRFVQ
AAF04434 At      AIDATK-KGSLARFINHSCRP--N---CETRKWN-----VLGEV---RVGIFA-KESISPRTELAYDYNFEW
XP_389092 Fg     IIDAT--TGSIARFVNHSCNP--N---CRMIKWI-----VSGQP---RMALFAGDKPIMTGDELTYDYNFDP
EAA34880 Nc      IIDAT--TGSIARFVNHSCSP--N---CRMIKWI-----VSGQP---RMALFAGDRPIQTGEELTYDYNFDP
XP_964116 Nc     IIDAT--TGSIARFVNHSCSP--N---CRMIKWI-----VSGQP---RMALFAGDRPIQTGEELTYDYNFDP
EAL92920 Af      IIDAT--RGSIARFVNHSCEP--N---CRMEKWT-----VAGKP---RMALFAGDHGIMTGEELTYDYNFDP
NP_524160 Dm     VIDGQR-MGSDCRFVNHSCEP--N---CEMQKWS-----VNGLS---RMVLFA-KRAIEEGEELTYDYNF--
NP_619620 Mm     VIDSYR-MGNEARFINHSCDP--N---CEMQKWS-----VNGVY---RIGLYA-LKDMPAGTELTYDYNF--
NP_196541 At     CPDKFGNISRFINGINNHNPVAKN---CKCVRYS-----INGEC---RVLLVA-TRDISKGERLYYDYNGYE
NP_197821 At     CPDRRSNIARFISGINNHSPEGRN---LKCVRFN-----INGEA---RVLLVA-NRDISKGERLYYDYNGYE
NP_084517 Mm     CVDATQETNRLGRLIN-HSKCG-N---CQTKLHD-----IDGVP---HLILIA-SRDIAAGEELLYDYGDRS
NP_650354 Dm     CIDATVDTGKLGRLIN-HSRAG-N---LMTKVVL-----IKQRP---HLVLLA-KDDIEPGEELTYDY----
XP_752474 Af     YVDGQK-FGSPTRFMNHSCNPNCK---LFPVTRT-Y---GDERLY--DLAFFS-LHNIPPNTELTFDYNPNW
```

```
NP_595186 Sp     YVDAQN-YGDVSRFFNHSCSPNIA---IYSAVRN-H---GFRTIY--DLAFFA-IKDIQPLEELTFDYAGAK
XP_382954 Fg     EVDGEF-MSGPTRFVNHSCEPNMR---IFARVGD-H---ADKHIH--DLALFA-IKDIPRGEELTFDYVDGV
EAA28243 Nc      EVDGEY-MSGPTRFINHSCDPNMA---IFARVGD-H---ADKHIH--DLALFA-IKDIPKGTELTFDYVNGL
XP_370355 Mg     VIDGEY-RSGPSRFFNHSCDPNMR---IFARVGA-H---AELNLH--DLAFFA-IRDISNGEELTFDYVDGQ
NP_073561 Mm     TVDAAR-YGNVSHFVNHSC-DPNL---QVFSVFIDN---LDTRLP--RIALFS-TRTINAGEELTFDYQ---
NP_035644 Mm     TVDAAY-YGNISHFVNHSC-DPNL---QVYNVFIDN---LDERLP--RIAFFA-TRTIWAGEELTFDY----
NP_524357 Dm     TIDAAN-YGNISHFINHSC-DPNL---AVFPCWIEH---LNVALP--HLVFFT-LRPIKAGEELSFDY----
NP_766133 Mm     CIDARF-YGNVSRFINHHC-EPNL---VPVRVFMSH---QDLRFP--RIAFFS-TRLIQAGEQLGFDYGERF
NP_665829 Mm     CIDARY-YGNISRFINHLC-DPNI---IPVRVFMLH---QDLRFP--RIAFFS-SRDIRTGEELGFDYGDRF
NP_569834 Dm     CIDANY-YGNVTRFFNHSC-EPNV---LPVRVFYEH---QDYRFP--KIAFFS-CRDIDAGEEICFDY----
AAD10665 At      CLDGMF-YGNISRFLNHRCLDANL---IEIPVQVET---PDQHYY--HLAFFT-TRDIEAMEELAWDYGIDF
AAK92218 At      SLEGTH-YGNISRFINHRCLDANL---IEIPVHAET---TDSHYY--HLAFFT-TREIDAMEELTWDYGVPF
AAF63769 At      CLDATI-CGNVARFINHRCEDANM---IDIPIEIET---PDRHYY--HIAFFT-LRDVKAMDELTWVSTMIK
AAC17088 At      AIDATT-HGNISRFINHSC-SPNL---VNHQVIVES---MESPLA--HIGLYA-SMDIAAGEEITRDYGRRP
AAH45208 Mm      FVDPTY-IGNIGRFLNHSCEP-NL---LMIPVRID------SMVP--KLALFA-AKDILPGEELSYDY----
NP_001097743 Dm  IVDPSR-RGNIGRYLNHSCEP-NC---HIAAVRI--------------------------------------
AAF00642 At      NIDATR-IGNVARFINHSCDGGNL---STVLLRSSG-----ALLP--RLCFFA-AKDIIAEEELSFSYGDVS
NP_611966 Dm     IMDAKT-TGNLGRYFNHSCSPNLF---VQNVFVD-T---HDLRFP--WVAFFS-AAHIRSGTELTWNYNYEV
NP_061365 Mm     IIDAKL-EGNLGRYLNHSCSPNLF---VQNVFVD-T---HDLRFP--WVAFFA-SKRIRAGTELTWDYNYEV
NP_001019872 Mm  LLDASK-EGNVGRFLNHSCCPNLW---VQNVFVE-T---HDRNFP--LVAFFT-NRYVKARTELTWDYGYEA
AAK28971 At      TIDAAS-KGNVGRFINHSCSPNLY---AQNVLYD-H---EDSRIP--HVMFFA-QDNIPPLQELCYDYNYAL
NP_181061 At     TINAAQ-KGNIGRFINHSCSPNLY---AQDVLYD-H---EEIRIP--HIMFFA-LDNIPPLQELSYDYNYKI
NP_196900 At     CIDAGS-TGNFARFINHSCEPNLF---VQCVLSS-H---QDIRLA--RVVLFA-ADNISPMQELTYDYGYAL
AAK28974 At      AMDVSK-MRNVACYISHSTDPNVI---VQFVLHD-H---NSLMFP--RVMLFA-AENIPPMTELSLDYGVV-
NP_180887 At     SMDVSR-MRNVACYISHSKEPNVM---VQFVLHD-H---NHLMFP--RVMLFA-LENISPLAELSLDYGLA-
AAK28966 At      IISAKN-VGNVARFMNHSCSPNVF---WQPVSYE-N---NSQLFV--HVAFFA-ISHIPPMTELTYDYGVSR
NP_565056 At     LISAKK-FGNVARFMNHSCSPNVF---WQPVIRE-G---NGESVI--HIAFFA-MRHIPPMAELTYDYGISP
AAK28973 At      LISAKE-KGNVGRFMNHNCWPNVF---WQPIEYDDN---NGHIYV--RIGLFA-MKHIPPMTELTYDYGISC
NP_564036 At     LISAKE-KGNVGRFMNHSCSPNVF---WQPIEYE-N---RGDVYL--LIGLFA-MKHIPPMTELTYDYGVSC
NP_178647 At     VISAKK-SGNVARFMNHSCSPNVF---WQSIARE-Q---NGLWCL--YIGFFA-MKHIPPLTELRYDYGKSR
EAA50044 Mg      LYTGD--KGNIFRFINHRCRS-PA---VKIEGMK-----ISGKYR--MMVVSV--RDVTAGEELTANWGRGF
XP_365330 Mg     IWTGE--EGNIFRFTNHKCAN-AE---AEVKGKK-----ISGAYR--MVLVAT--QDILHGMEVCVDFGQKF
EAL89869 Af      IYQGE--MGNFTRFINHSCR--PN---SQFQRFY-----WRGQER--IIVVSR---GVVAGREITVDYSDHY
XP_383195 Fg     IWQGK--QGNFTRFANHSCK--PN---AQFEKFV-----WLGTQH--IVMNN------TADIPLLFRFNMIN
XP_357091 Mm     --------GNWMSYVNCARFPKEQ---NLLAV---------QHQ---GQIFYESCRDIQRNQELLVWYGNGY
NP_031574 Mm     -------KSNWMRYVNPAHSAREQ---NLAAC---------QNG---MNIYFYTIKPIPANQELLVWYCRDF
NP_001028453 Mm  -------KSSWMRYIRCARHCGEQ---NLTVV---------QYR---SNIFYRACIDIPRGTELLVWYNDSY
EAL91753 Af      ALISAKQHGNWTRYLNHS-CKPST---TFLKMTVG------KR----TIMAVQAVRDIDIFEEITVDYG---
XP_964337 Nc     ADIYPQQMGNWVRKVRHDGVSPSA---VFKVMKITG-----KWR-----VMLVAMRDIRDGDEITAKYGRGY
XP_449432 Cg     REYFGYWVFPKASYFNHSCDPN------ITKT---------RID---RKMVFTLNRDVACGEELNIDYSGVL
NP_015160 Sc     REYFGYWVFPEASYFNHSCNPN------ITKY---------RKG---NSMLFTMNRDIKKDEQICIDYSGVL
NP_982445 Ag     RDYLGYWVLPEASYFNHSCAPN------LAKK---------RVG---REMYFVLTSDVAAGEQLCIDYKGIL
EAK94622 Ca      KEFLGFGVYPSASFFNHSCSPN------IVKT---------RNN---SEMVFTTSKDIEIGEELCISYGNYT
NP_983709 Ag     LNQVNGQIYMLLSHLNHSCEPN------IYYELEG------HH------INVYARKEIKSDEELTVSYVNPL
P38890 Sc        INQYNGQVYHWISFINHDCEPN------AYIEQVE------EHE---E-LRLHARKPIKKGEQIRITYVNPL
XP_446364 Cg     INQLSGQLYFLYSFLNHNCEPN------VRYDINN------KL----E-LKVYARKFIKKDEELVTTYVNPL
EAK96662 Ca      INNLDSNVFLTQSHLNHNCASN------TSVETEL------NRT---AGLKVIAGRDIKSGEELTTTYVNPS
XP_385929 Fg     -----DEVFLATSRINHSCKPN------AQNRWN-------QGL---GKITVHAVEDIEQGQEITITYLGNP
XP_391443 Fg     -----MDVFLQASRINHACDNN------AQKDYN-------EGI---KRHTVHALRDIEEGEEITITYLGIL
NP_588413 Sp     ------EMFLLGSRMNHDCSPN------VKHTWN-------PRL---DQVTVHAVRDIEAGEEILTTYIDLH
EAA33925 Nc      -----PPLFIEASRINHACNPN------TQNSWN-------SRI---NRETIHAVRDIKKGEEITISYIGHF
EAA49663 Mg      ---RFHSTFVEVSRMNHECSPN------CAAYFD-------PMT---MSQRIYAIRDIMPGEELTVSYIDPV
XP_362227 Mg     ---QFYVVFPEISRCNHDCRPN------ADYYYD-------PET---LTQHVHAVRQINIGEEISLSYLDPL
XP_366867 Mg     HRKAVPAVFRNASRLNHSCVPN------CHFSWD-------RVT---RTCLVHAITDVSPGDELTVAYDSRG
XP_364335 Mg     ---DYMGLYPEIARINHACSPN------SFSRFH-------PSD---LTMDVGAMRDIMPGEEITISYIPLG
XP_370301 Mg     ---GYMGLFPTISRINHACGPN------SQVKFD-------PAT---LSQKAFASHDIEAGEEITISYAEFG
XP_368905 Mg     ----HAGTFLGVSRLNHDCRPNNP----TSLAFHV------AKS---FVHTTHATRDIAAGEELTISYVDSY
XP_364537 Mg     ----HYGNFPNVSKLNHDCRPN--------MVFHI------DNN---LVHRTHAVRDIKPGEELTISYVDQM
EAA26997 Nc      DHSKHYINYPSMFLFTHSCRPN--------IAFHI------DGT---LALRTTVA--VAPGEELSIAYIDPL
XP_363030 Mg     EESQHLGVYAQAAAINHDCRPS------------------------INYRLNDITQIQPGEELSVSYVDLM
NP_572675 Dm     ----ARTLYPSAFLLAHDCTPN------TAHTDDPS-----S-----FEILLRTSRRVREREALTLSYAYTL
NP_609464 Dm     ----LRCLFPYTGILAHNCVPN------TSRSIYPS---E-G-----YKIRLRAMVDLEEGQPLHHSYTYTL
```

```
NP_649084 Dm     ----IRALYPGAAMISHDCVPN------MRHRFDDD----------MNIVFLAKRKIAKGEILSISYTQPL
NP_610944 Dm     ----VAVFYT-ASFTENSCLPN------LAKSFNKN----------GHCILWAPREIKKNAHLSICYSDAM
NP_001014717 Dm ---LLRGLFPLTAIMNHECTPN------ASHYFENG-----R------LAVVRAARDIPKGGEITTTYTKIL
CAA15694 Dm      ---LVRALFPLAGLLNHQCTPN------AAHHFENG-----E------TIVVCATERIPAGAEITMSYAKLL
NP_572539 Dm     ---NYRALYPLFGVVNHDCIPN------AYYTFEEK----TN------NMIVRAAVDIPEGFEVTTTYTKLF
NP_033892 Mm     ----GVGIFPNLGLVNHDCWPN------CTVIFN------NG------KIELRALGKISEGEELTVSYIDFL
NP_081072 Mm     ----GSAIFPDVALMNHSCCPN------VIVTYK-------GT-----LAEVRAVQEIHPGDEVFTSYIDLL
NP_081464 Mm     ----GVGLYPSMSLLNHSCDPN------CSIVFN-------GP-----HLLLRAVREIEAGEELTICYLDML
XP_360530 Mg     ----GTFLDPVLAMANHSCVPN------AVVLFW-------RR-----KAYLRAEMPIKQGSEISISYIDYT
XP_381344 Fg     ----GIFLGTKLAMANHSCIPN------AMVQFI-------GR-----RAILRAEKPIKIDDEIEISYTDYT
EAA36113 Nc     ----GIYLHPSLSMVNHSCVPN------AYIAFE-------KR-----KAFLKAERDLEPGDEILISYIGMA
NP_650955 Dm     ----GSGLYLLQSKINHSCVPN------ACSTFP------YSN----DIVVLKALAPIQQGEEICISYLDEC
NP_659167 Mm     ----GSGLFVLQSCCNHSCVPN------AETSFP------ENN----FVLHVTALEDIKPGEEICISYLDCC
XP_384827 Fg     PLFTTAGVWLLAARINHSCVGN------CRRSFIGD-----IQI---VR----AARDIPAGTELTFPYCPTG
XP_380216 Fg     FKYTTSGIWLLASRINHSCVGN------CRPSFIGD-----MQI---VR----ATKDVPAKTEIFFCYRPPV
EAL91344 Af     -KFHSCGVWLLASYINHSCCSN------ARRSFIGD-----MMI---VR----AAQDLAAGTEITFWYQSPL
XP_367445 Mg     GPESSTGMWLHAAYANHTCIPN------ATRAFIGD-----MMI---VR----AARDIPAGAEIFMGYASLA
XP_366224 Mg     QPPSARGLWPLAAAMNHACVAS------TVRAFVGD-----VFI---TR----ALRDIEPGEELTQQYVPVR
EAA35550 Nc     SVCMAKGLWAHSSLMNHSCVPN------TMRSFVGD-----MLI---CR----ATRDVQEGEELFQQYVPVK
AAF87042 At     KEYYGVGLWTLASFINHSCIPN------ARRLHVGD-----YVI---VH----ASRDIKTGEEISFAYFDVL
XP_387208 Fg     GRPDVAAVHPYWCLANHDCDPN------VTWEWGGR-----MVL---EARKERVVGGIKKGEEILNHYCDVN
XP_957213 Nc     GRPEVAAVHPFWCLANHDCDPN------VTWEWGGR-----MTL---FARETRVVGNIKAGEEILNHYCDVD
XP_365932 Mg     GRPDVAAVHPFWCLANHDCDPN------VTWEWSGR-----MVL---RAKEERVEWGIKAGDEILNHYCDVN
XP_367482 Mg     ---STSLFMGPARFANHDCGAN------ARLKT--------ADQ---SIMEVQALRNIEVGEEITVTYGDNY
EAA33797 Nc     ---STSLFMGPARFANHDCNAN------ARLIT--------RGQ---AGIEIIACRNIEVGEEITVTYSESY
XP_386705 Fg     ---CTSLFMGPARFANHDCDAN------AKLMR--------TSH---AGIEIVATRPIDAGEEITVTYGDNY
EAL93147 Af     ---TPSFFLGPARFANHDCNAN------GKLVT--------RGS---EGMQVVATRDIYIGEEITVSYGDDY
NP_588078 Sp     ---SMCLFLGPARFVNHDCNAN------CRFNT--------SGK---R-IWLRCVRDIKPGEEITTFYSSNY
AAF45537 Dm     ---CAQLWLGPAAYINHDCRAN------CKFLA--------TGR---DTACVKVLRDIEVGEEITCFYGEDF
NP_659120 Mm     ---CAQLWLGPAAFINHDCRPN------CKFVS--------TGR---DTACVKALRDIEPGEEISCYYGDGF
NP_666289 Mm     ---SAQLWLGPAAFINHDCKPN------CKFVP--------SDG---NTACVKVLRDIEPGDEVTCFYGEGF
EAL85588 Af     ----SKGMVPLADILNADADRNN-----ARLF---------QED---DSLVMKAIKPIRVGEEIFNDYGELP
XP_389160 Fg     ----MMGMVPMADILNADAEFN------AHVN---------HEE---ESLTVTSLRPIKAGEEILNYYGPHP
XP_389761 Fg     IDTRQEGSLA--RYVRRSCKPN-----AQLDTYLSDES-------E-YHFWIVSDRHISANEQITLP-----
XP_363632 Mg     IDTRQEGSRA--RYVRRSCKPN-----AVLDTFLSGGS-------E-YHFWLVSDRSIAANEQITIA-----
EAA28230 Nc     IDTRKEGSQA--RYIRRSCKPN-----AMLETYLSDGY-------E-LHFWLVTDRQVAAREEITLP-----
EAL93416 Af     IDSRHEGSIL--RYVRRSCRPN-----VTIKTYITNEV-------E-YHFCFVAKEDISANSEITAM-----
NP_648681 Dm     VDTRTYGNDA--RFVRRSCRPN-----AELQHYFEKG--------T-LHLYIVALTHIRAQTEITIR-----
NP_082661 Mm     VDARTFGNDA--RFIRRSCTPN-----AEVRHMIADG--------M-IHLCIYAVSAITKDAEVTIA-----
XP_448906 Cg     IDARSKGNLT--RFLRRGCKPN-----VELVTVCLPSAG-EEKD---IKFILRATRDIDEGEELLID-----
NP_012954 Sc     IDARLSGNST--RYLRRSCQPN-----VELVTIKLQDTD-NRNDKR-IKFVLRALRDISEDEELYIK-----
NP_985624 Ag     IDARLCGNLT--RYLRRSCYPN-----VELVTIKMPADD-RQAS-K-VKFVLKALRDLERGEELHIK-----
XP_446555 Cg     INTSGTKGAHNVEHLRCSCNPN-----VELVTVRIMDNK-PR-----IKFVIRAIRDIAEGEELQIA-----
NP_012430 Sc     IDSRETGGLT--RYIRRSCEPN-----VELVTVRPLDEK-PRGD-C-VKFVLRAIRDIRKGEEISVE-----
EAA30745 Nc     RIIDAGKMGNWTRFMNSSCDPN-----VSESIMQIG---------V-RVIAFFANKTLKSGDELCIYYGDDY
XP_001481661 Af QPENAKVLVLGLAFVNHACIAN---SLYYTLRYPRD---ENGEEDI-GRAVVRASRDIRPGEEITVAYFYAK
XP_384009 Fg     DISNASTGLWVRSYINHSCIPN-----AKKDLIG-------------DLILFRATRRIASGEEITHAYDEST
BAB11411 At     GEGAVGHAVYMPSFYNHDCDPN------AHIIWLHN-----------ADARLNTLRDVEEGEELRICYIDAS
NP_588361 Sp     --EKQGLALGLGSMFNHDRHPN-----VYWKKDNRN-----------NYISYYTLREIKTNEELCISYGDHL
EAA34532 Nc     NPYSSDIISVYTSRLNHACRSVAN----VLFDFDF------QDP---ECITLTVSKPVKAGSELFISYGGSP
XP_381576 Fg     RCYDEAGLFPIASRFNHACEPMNS----VKWHYN------HADG----NLKMTIQAKIEAGKELTITLPYFS
NP_193253 At     VVVDAMHMANYASRICHSCRPN-----CEAKVTAVDG---------HYQIGIYSVRAIEYGEEITFDYNSVT
NP_594837 Sp     LVVDSRVAGSKARFARKGCQSN----SVVSSVYMN----GSNSVP---RFILYSTTHIAPETEIIGD-----
EAK95983 Ca     IVLDSRFVGNESRFIRKACPSSAN---CRIETVYVP---EQNKFR---FLVFTSKPITLKSENQDEELRLP-
XP_713530 Ca     QRQELGIALDPFALINHSCIPN-----CCQITNDCN------------EFQIVSTLPINNGEELTVTYVSLG
NP_542983 Mm     YNHVSKYCASLGHKANHSFTPN-----CVYDLFVHP-----RFG---PIKCIRTLRAVEAEEELTVAYGYDH
NP_565457 At     SNEKRSVRAYGTSFFNHDCLPN-----ACRFDYVDS---ASDGN---TDIIIRMIHDVPEGREVCLSYFPVN
CAE76340 Nc     STSTPPTPTTNGSLINHCCTSPSAY--VARKRTERG---PNCEWG--LAHFVKPKHIIKEGEQLTWDYGKKE
```
