# Supplementary material for: Evolution of SET-domain protein families in the unicellular and multicellular Ascomycota fungi
Source: BMC Evol Biol. 2008 Jul 1;8:190. doi: 10.1186/1471-2148-8-190 (PMC2474616; doi:10.1186/1471-2148-8-190)
Supplement: Additional file 5 — The multiple sequence alignment of 113 representative SET-domain sequences. [file 1471-2148-8-190-S5.pdf]

Multiple sequence alignment of representative SET-domain sequences. The CLUSTAL X (1.83) multiple alignment of 113 representative SET-domain sequences used to reconstruct the maximum likelihood (Fig. 1) and maximum parsimony (Additional file 9) phylogenies.

```
XP_389761 Fg              ---------KALRWRFLKSSTPIEKDVPLVELNGAIGFQKDYCADPTNL---WADLSSPL--PFVFFHV-LPLYIDTRQEGSLARYVRRS-CKPNAQLDTYLSDE-SE--YHFWIVSD-RHISANEQITLPWDFRLEKS
XP_363632 Mg              -KQLSLARETVARWQYLMTPTPVAKDVPLIELNGSIGFQKDYCADKHNR---WDEFSSPL--PFVFFHM-LPLYIDTRQEGSRARYVRRS-CKPNAVLDTFLSGG-SE--YHFWLVSD-RSIAANEQITIAWDFRFPNP
EAA28230 Nc              -SRKTLGQGTVASWKCLKAPSAIAHNVPLVELNGQIGIQSSYCADPDSR---WQELTSPL--PYVFFHM-LPIYIDTRKEGSQARYIRRS-CKPNAMLETYLSDG-YE--LHFWLVTD-RQVAAREEITLPWDFRFPNE
EAL93416 Af              --PSINIEGQHPTWKVLRTRESVSKDHIVGEITGKIGLLRDYCLDPSNR---WQELRHPE--PFVFFHQ-LPIYIDSRHEGSILRYVRRS-CRPNVTIKTYITNE-VE--YHFCFVAK-EDISANSEITAMWYLDPQLF
NP_082661 Mm              QLGRVTRVQKH--RKILRAARDLALDTLIIEYRGKVMLRQQFEVNG-------HFFKKPY--PFVLF-G-VEMCVDARTFGNDARFIRRS-CTPNAEVRHMIADG----MIHLCIYAV-SAITKDAEVTIAFDYEYSNC
NP_648681 Dm              --AQLIPYAG---AKVLISSVDLSPHAPIHELRGKYMLTTQFRTQNPTV---NKAHKTPG--QFVFFQG-PEVCVDTRTYGNDARFVRRS-CRPNAELQHYFEKG----TLHLYIVAL-THIRAQTEITIRHEPHDLTA
XP_448906 Cg              -ESSYARTFPAHTKLGVFTKEYCNNGDLIEEFTGQVDFLKTYLDDTKNH---YRIWGTAK--NRVIFHH-WPLYIDARSKGNLTRFLRRG-CKPNVELVTVCLPSAEK--DKFILRAT-RDIDEGEELLIDWKWDLRHP
NP_012954 Sc SET3         -DIAYSRTYPGFTKLGVYLKKDCIKGDFIQEILGELDFYKNYLTDPRNH---YRIWGTAK--RRVIFHH-WPIYIDARLSGNSTRYLRRS-CQPNVELVTIKLQDTRN--DKFVLRAL-RDISEDEELYIKWQWDSKHP   SET3/4
NP_985624 Ag              -EPSHSRVFHGFPKLGVYSQQPCAKDTLIAEFLGEVDFQRKYLEDPRNN---YRLLGIPN--PKVLFHH-WPIYIDARLCGNLTRYLRRS-CYPNVELVTIKMPADQA--SKFVLKAL-RDLERGEELHIKWDWDLRHP
XP_446555 Cg              --VLKPLASKDIQDFGVFTSIPCSKKDYIQEYSGMISFSKQYVNNAENK---YDILGTPT--RNILFHH-WPLYINTSGTKGAHEHLRCS-CNPNVELVTVRIMDNR----KFVIRAI-RDIAEGEELQIAWQWDINHP
NP_012430 Sc SET4         -ADIEVRKSSNERDFGVFAADSCVKGELIQEYLGKIDFQKNYQTDPNND---YRLMGTTK--PKVLFHH-WPLYIDSRETGGLTRYIRRS-CEPNVELVTVRPLDERG--DKFVLRAI-RDIRKGEEISVEWQWDLRNP
EAK95983 Ca              --ALFISSNGGGSGGGSLT---IPENTPIIEYLGEIDLFKNYCRDSINQ---YRMWGSPK--PTQYSDT-LDIVLDSRFVGNESRFIRKA-CPSNCRIETVYVPE--K--FRFLVFTS-KPITLKSENQDPWEWDVDHP
NP_594837 Sp              --VPISSSKFCCSRFGLVSTCEIPPNTPIMEVKGRVCTQNEYKSDPKNQ---YNILGAPK--PHVFFDN-SQLVVDSRVAGSKARFARKG-CQSNSVVSSVYMNG--SV-PRFILYST-THIAPETEIIGDWTLDISHP
EAA50044 Mg              EGLYVSHYGKK--GFGIRVT-FYGRRHVFGELVGNLVKPGSFSDGMGLVF-ARDDLDEQ----------PVTAQLYTGDKGNIFRFINHRCRSPAVKIEGMKISG----KYRMMVVSV-RDVTAGEELTANWGRGFIEG
XP_365330 Mg              --PSVVYFDGK--ELGLQAMAAYRKGTLIGEIVGRIVPPNTYNDGRAMNM-VREDMDN-----------EYVGQIWTGEEGNIFRFTNHKCANAEAEVKGKKISG----AYRMVLVAT-QDILHGMEVCVDFGQKFDPC   SET-JMJC
EAL91753 Af              --VELVDYPQR--GIGVRALARFKKGDILDEYVGELRPLDYTDDRVYGLL-HESKMTEG----------HPLALISAKQHGNWTRYLNHS-CKPSTTFLKMTVGK----RTIMAVQAV-RDIDIFEEITVDYG------
XP_964337 Nc              QIPRITQDGSR--GEGVRAIGSFKADECLGELVGELRPPRTAGTSCSSDH-NHPNISSGQAQRRPDLHDQLVADIYPQQMGNWVRKVRHDGVSPSAVFKVMKITG----KWRVMLVAM-RDIRDGDEITAKYGRGYRKE
NP_031996 Mm              -------SDVA--GWGTFIKESVQKNEFISEYCGELISQDE----ADRR-GVYD--KYMSS---FLFNLNNDFVVDATRKGNKIRFANHS-VNPNCYAKVMVNGD----HRIGIFAK-RAIQAGEELFFDYRYSQAD-
NP_524021 Dm E(z)         KHLLMAPSDIA--GWGIFLKEGAQKNEFISEYCGEIISQDE----ADRR-GVYDK--YMCS---FLFNLNNDFVVDATRKGNKIRFANHS-INPNCYAKVMMVTGD----HRIGIFAK-RAIQPGEELFFDYRY-----
CAA71599 At              QRVLLGISDIS--GWGAFLKNSVSKHEYLGEYTGELISHKE----ADKR-GIYDR--ENCS---FLFNLNDQFVLDAYRKGDKLKFANHS-PEPNCYAKVIMVAGD----HRVGIFAK-ERILAGEELFYDYRYEPDRA   E(z)
AAC39446 At              KKILIGKSDVH--GWGAFTWDSLKKNEYLGEYTGELITHDE----ANER-GIEDR--IGSS---YLFTLNDQLEIDARRKGNEFKFLNHS-ARPNCYAKLMIVRGD----QRIGLFAE-RAIEEGEELFFDYCYGPEHA
XP_381075 Fg              --LALGQSQLEGVGYGLFTVEPIAQDDFIIEYVGELITHDEGVRREARR-GVFDEE-SNIS---YVFTLLENEWVDAATYGNLSRYINHA-SESNITPRILYVNGE----YRIKFTAM-RDIAAGEELFFNYGENFPNL
EAA35807 Nc              --VLLGKSQLEGCGYGLFTAEDISQDEFVIEYTGELITHDEGVRREARR-GGFGSQ-GTSS---YLFTLLEHEWVDAAMYGNLSRYINHA-SENNITPKIIYVNNE----YRIKFTAL-RDIKAGEELFFNYGDNFPNL
XP_369092 Mg              --LCLGESGIEGCGYGLFTAVDIAADEFIIEYVGELIQHDEGVRREARR-GVFDEE-SNVS---YLFTLLEDDWVDAAVYGNLSRYMNHA-SESNVVPKIVQVNGD----FRIRFTAL-RDIKAGEELFFNYGENFPNL
XP_387621 Fg              KPVKFARSAIH--NWGLYAMENIAKDDMIIEYVGEQVRQQISEIRENR----YLKSGIGSS---YLFRIDDNTVIDATKKGGIARFINHS-----------FEGS----KRIVIYAL-RDIALNEELTYDYKFEREIG
XP_961572 Nc              KPVKFARSAIH--NWGLYAMENINKDDMIIEYVGEEVRQQIAELREAR----YLKSGIGSS---YLFRIDDNTVIDATKKGGIARFINHS-CMPNCTAKIIKVEGS----KRIVIYAL-RDIAQNEELTYDYKFEREIG
XP_750524 Af              KPVRFARSAIH--NWGLYAEENISANDMIIEYVGEKVRQQVADMRERQ----YLKSGIGSS---YLFRIDENTVIDATKRGGIARFINHS-CTPNCTAKIIKVDGS----KRIVIYAL-RDIGRDEELTYDYKFEREWD
XP_449333 Cg              KPVTFARSAIH--NWGLYALEPINAKEMVIEYVGERIRQPVAEMRERR----YIKNGIGSS---YLFRIDEHTVIDATKKGGIARFINHC-CEPSCTAKIIKVGGK----RRIVIYAL-RDIAANEELTYDYKFERETD
NP_983083 Ag              KPVTFARSAIH--NWGLYALEPISAKEMIIEYVGERIRQPVAEMREKR----YLKSGIGSS---YLFRVDESTVIDATKKGGIARFINHC-CDPSCTAKIIKVGGM----KRIVIYAL-RDIAANEELTYDYKFERETD
NP_011987 Sc SET1         KPVMFARSAIH--NWGLYALDSIAAKEMIIEYVGERIRQPVAEMREKR----YLKNGIGSS---YLFRVDENTVIDATKKGGIARFINHC-CDPNCTAKIIKVGGR----RRIVIYAL-RDIAASEELTYDYKFEREKD
EAK99965 Ca              KPVTFARSAIH--NWGLYAMEPIAAKEMIIEYVGERIRQQVAEHREKS----YLKTGIGSS---YLFRIDDNTVIDATKKGGIARFINHC-CSPSCTAKIIKVEGK----KRIVIYAL-RDIEANEELTYDYKFERETN
XP_001411780 Mg          KPVKFERSAIH--NWGLYAMEHIPKDDMIIEYVGEEVRPSVAQVREAR----YDRSGIGSS---YLFRIDEDAVIDATKKGGIARFINHS-CMPNCTAKIIRVEGT----KRIVIYAL-RDIARNEELTYDYKFELEEK   SET1
NP_587812 Sp              KQLHFGPSRIH--TLGLFAMENIDKNDMVIEYIGEIIRQRVADNREKN----YVREGIGDS---YLFRIDEDVIVDATKKGNIARFINHS-CAPNCIARIIRVEGK----RKIVIYAD-RDIMHGEELTYDYKFPEEAD
BAC65717 Mm              ------KSHIH--DWGLFAMEPIAADEMVIEYVGQNIRQVIADMREKR----YEDEGIGSS---YMFRVDHDTIIDATKCGNFARFINHS-CNPNCYAKVITVESQK----KIVIYSK-QHINVNEEITYDYKFPIED-
BAB10481 At              KHLRFQQSKIH--DWGLVALEPIEAEDFVIEYVGELIRSSISEIRERQ----YEKMGIGSS---YLFRLDDGYVLDATKRGGIARFINHS-CEPNCYTKIISVEGKK----KIFIYAK-RHIDAGEEISYNYKFPLEDD
NP_476769 Dm trx          ------RSHIH--GRGLYCTKDIEAGEMVIEYAGELIRSTLTDKRERY----YDSRGIGC----YMFKIDDNLVVDATMRGNAARFINHC-CEPNCYSKVVDILGHK----HIIIFAL-RRIVQGEELTYDYKFPFEDE
NP_726773 Dm              --VYLARSKIQ--GLGLYAARDIEKHTMIIEYIGEVIRTEVSEIREKQ----YESKNRGI----YMFRLDEDRVVDATLSGGLARYINHS-CNPNCVTEIVEVDRDV----RIIIFAK-RKIYRGEELSYDYKFDIEDE
AAK01237 At              KRLAFGKSGIH--GFGIFAKLPHRAGDMMIEYTGELVRPSIADKREQL---IYNSMVGAG---TYMFRIDDERVIDATRTGSIAHLINHS-CVPNCYSRVITVNGDE----HIIIFAK-RHIPKWEELTYDYRFFSIGE
BAA97320 At              ERVCFGRSGIH--GWGLFARRNIQEGEMVLEYRGEQVRGIIADLREAR----YRREGKDC----YLFKISEEVVVDATEKGNIARLINHS-CMPNCYARIMSVGDDES---RIVLIAK-TTVASCEELTYDY-LFDPDE
AAC23419 At              KKMKLIQTEK--CGSGIVAEEEIEAGEFIIEYVGEVIDDKTCEERLWK----MKHRGET---NFYLCEITRDMVIDATHKGNKSRYINHS-CNPNTQMQKWIIDG----ETRIGIFAT-RGIKKGEHLTYDYQ-FVQFG
CAA18207 At              KKIKIVKTEH--CGWGVEAAESINKEDFIVEYIGEVISDAQCEQRLWDM-K---HKGMKD---FYMCEIQKDFTIDATFKGNASRFLNHS-CNPNCVLEKWQVEG----ETRVGVFAA-RQIEAGEPLTYDYR-FVQFG
NP_001001735 Mm          ------------GWGLRTKRSIKKGEFVNEYVGELIDEEECRLRIKR-----AHENSVT--NFYMLTVTKDRIIDAGPKGNYSRFMNHS-CNPNCETQKWTVNG----DVRVGLFAL-CDIPAGMELTFNY-------
XP_385734 Fg              ANVSVIKTEK--KGFGLRADSDLQPNDFVFEYIGEVINEPTFRRRMIQ----YDEEGIK---HFYFMSLNKSEFVDATKKGNYGRFCNHS-CNPNCYVDKWVVGD--KL--RMGIFTS-RKIQSGEELVFNYN-VDRYG
XP_957740 Nc              ADVSVIKTEK--KGFGLRANTDLQVNDFIFEYIGEVINEPTFRSRMVK----YDKEGIK---HFYFMSLTKSEFVDATKKGNLGRFCNHS-CDPNCYVDKWVVGD--KL--RMGIFAG-RAIKAGEELVFNYN-VDRYG
EAL91994 Af              ANVAVIKTEK--KGFGLRAETDLRPHQFIFEYVGEVINEAQFRRRMRQ----YDEEGIK---HFYFMSLSRGEFVDATKKGNLGRFCNHS-CNPNCYVDKWVVGE--KL--RMGIFAE-RAIQAGEELVFNYN-VDRYG
XP_363735 Mg              ANVSVIKTEN--KGYGLRADANLEPNDFVFEYIGEVIGEELFRSRLMK----YDTQRLE---HFYFMSLTRTEYVDATKKGNLGRFCNHS-CNPNCYVDKWVVGD--KL--RMGIFAM-RAIKAGEELCFNYN-VDRYG
NP_594980 Sp              AKVDVFLTEK--KGFGLRADANLPKDTFVYEYIGEVIPEQKFRKRMRQ----YDSEGIK---HFYFMMLQKGEYIDATKRGSLARFCNHS-CRPNCYVDKWMVGD--KL--RMGIFCK-RDIIRGEELTFDYN-VDRYG
XP_445194 Cg              ANIAVFKTKM--KGYGVRAESDIEINDFIYEYKGEVIEEEEFRDRLVD----YDQKKFR---HFYFMMLQSGEFIDATIKGSLARFCNHS-CNPNAYVNKWVVAG--KL--RMGIFAK-RKILKGEEITFDYN-VDRYG
NP_012367 Sc SET2         APIAIFKTKH--KGYGVRAEQDIEANQFIYEYKGEVIEEMEFRDRLID----YDQRHFK---HFYFMMLQNGEFIDATIKGSLARFCNHS-CSPNAYVNKWVVKD--KL--RMGIFAQ-RKILKGEEITFDYN-VDRYG   SET2
NP_984733 Ag              ADIAVFQTEK--KGYGVRAERDIEANEFIYEYIGEVISEADFRDRMVD----YDMRGFK---HFYFMMLQAGEFIDATERGCLARFCNHS-CNPNAYVSKWDVAG--KL--KMGIFAH-RKILKGEEITFDYN-VDRYG
EAK95292 Ca              SKVKVIQTEL--KGYGLIAEQDIEENQFIYEYIGEVIDEISFRQRMIE----YDLRHLK---HFYFMMLSNDSFIDATEKGSLGRFINHS-CNPNAFVDKWHVGD--RL--RMGIFAK-RKISRGEEITFDYN-VDRYG
AAF04434 At              AKTKLIKCEG--RGWGLVALEEIKAGQFIMEYCGEVISWKEAKKRAQT----YETHGVK---DAYIISLNASEAIDATKKGSLARFINHS-CRPNCETRKWNVLG----EVRVGIFAK-ESISPRTELAYDYN-FEWYG
AAC34358 At              VKFERFQSGK--KGYGLRLLEDVREGQFLIEYVGEVLDMQSYETRQKE----YAFKGQK---HFYFMTLNGNEVIDAGAKGNLGRFINHS-CEPNCRTEKWMVNG----EICVGIFSM-QDLKKGQELTFDYN-YVRVF
NP_572888 Dm              -PCRVFRTEK--KGCGITAELLIPPGEFIMEYVGEVIDSEEFERRQHL----YSKDRNR---HYYFMALRGEAVIDATSKGNISRYINHS-CDPNAETQKWTVNG----ELRIGFFSV-KPIQPGEEITFDYQ-YLR--
EAA34880 Nc              IGVEVFKTED--RGYGVRSNRCFEPHQIIMEYTGEIITDEECERRMNE---EYKNNEVCFP-CYYLMSFDQNMIIDAT-TGSIARFVNHS-CSPNCRMIKWIVSG--QP--RMALFAGDRPIQTGEELTYDYN-FDP--
XP_389092 Fg              VGVEVIKTSD--RGYGVRSNRCFRPNQIIMEYAGEIITEEECERRMTE---VYKDNE-----CYYLMSFDQNMIIDAT-TGSIARFVNHS-CNPNCRMIKWIVSG--QP--RMALFAGDKPIMTGDELTYDYN-FDPFS
XP_964116 Nc              --FQMLTERT--K----KGGRYRIGVEIIMEYTGEIITDEECERRMNE---EYKNNE-----CYYLMSFDQNMIIDAT-TGSIARFVNHS-CSPNCRMIKWIVSG--QP--RMALFAGDRPIQTGEELTYDYN-FDPFS
EAL92920 Af              IGVEVIKTAD--RGYGVRSNRTFEPNQIIVEYTGEIITQAECEKRMRT---IYKNNE-----CYYLMYFDQNMIIDAT-RGSIARFVNHS-CEPNCRMEKWTVAG--KP--RMALFAGDHGIMTGEELTYDYN-FDPYS
NP_524160 Dm ash1         -------TAD--KGWGVRTKLPIAKGTYILEYVGEVVTEKEFKQRMAS---IYLNDT-----HHYCLHLDGGLVIDGQRMGSDCRFVNHS-CEPNCEMQKWSVNG----LSRMVLFAK-RAIEEGEELTYDYN-F----
NP_180887 At              NRLEVFRSKE--TGWGVRTLDLIEAGAFICEYAGVVVTRLQAEILSMN----GDVMVYPG--VYPDFVLD--FSMDVSRMRNVACYISHS-KEPNVMVQFVLHDHNHL-MFPRVMLFALENISPLAELSLDYGLA-DEV
AAK28971 At              LPLEIFKTKS--RGWGVRCLKSIPIGSFICEYVGELLEDSEAERRIGN----DEYLFDIG--TQAGRSMA--FTIDAASKGNVGRFINHS-CSPNLYAQNVLYDHEDS-RIPHVMFFAQDNIPPLQELCYDYNYALDQV
NP_565056 At              SRLEVFKTRN--RGWGLRSWDSLRAGSFICEYAGEVKDNGNQEEDAYV---FDTSRVFN-----EDPSPE--LLISAKKFGNVARFMNHS-CSPNVFWQPVIREGNGE-SVIHIAFFAMRHIPPMAELTYDYGISPTSE
AAF00642 At              VSLKIVRD-EK-KGWCLYADQLIKQGQFICEYAGELLTTDEARRRQNI--KLRSTQSF----LVVREHL-LRINIDATRIGNVARFINHS-CDGGNLSTVLLRS--SGALLPRLCFFAAKDIIAEEELSFSYGDVSVAG
AAH45208 Mm              ------------GWGLRTLEFIPKGRFVCEYAGEVLGFSEVQRRIHL---QTSHDSN----IAVREHI-METFVDPTYIGNIGRFLNHS-CEP-NLLMIPVR---IDSMVPKLALFAAKDILPGEELSYDY-----SG
AAC17088 At              AKLEVFRT-ES-KGWGLRACEHILRGTFVCEYIGEVLDQQEANKRRNQ--YGNGDCSYI---DANINDIGLDYAIDATTHGNISRFINHS-CSP-NLVNHQVIVESMESPLAHIGLYASMDIAAGEEITRDYGRRPVPS
AAF63769 At              CQLQVYFTQEG-KGWGLRTLQDLPKGTFICEYIGEILTNTELYDRNVR--SSSERHTYP---DADWGSE-EALCLDATICGNVARFINHR-CEDANMIDIPIEIETPDRHYYHIAFFTLRDVKAMDELTWVSTMIKLP-
NP_073561 Mm              ------------GWGVKTLVKIKRMSFVMEYVGEVITSEEAERRGQF----YDNKGI-----TYLFDLD-EFTVDAARYGNVSHFVNHS-CDPNLQVFSVFIDNLDT-RLPRIALFSTRTINAGEELTFDYQ------
NP_524357 Dm Su(var)3-9   ---VLFKTANG-SGWGVRAATALRKGEFVCEYIGEIITSDEANERGKA----YDDNG------TYLFDLDSEYTIDAANYGNISHFINHS-CDPNLAVFPCWIEHLNV-ALPHLVFFTLRPIKAGEELSFDY------   Su(var)3-9
NP_766133 Mm              ------RTQD--MGWGVRSLQDIPLGTFVCEYVGELISDSEADVREE--------------DSYLFDL--VYCIDARFYGNVSRFINHH-CEPNLVPVRVFMSHQDL-RFPRIAFFSTRLIQAGEQLGFDYGERFWD-
NP_061365 Mm              VRLQLFKTQN--KGWGIRCLDDIAKGSFVCIYAGKILTDSGVDMKDQE----DGNSGSEDP-DSRPPDLGSCYIIDAKLEGNLGRYLNHS-CSPNLFVQNVFVDTHDL-RFPWVAFFASKRIRAGTELTWDYNYEVGSV
XP_382954 Fg              IPLEIFRTED--RGWGVRSPVSIRKGQFVDRYLGEIITSTEADRRRSQ---SAISQRK----DVYLFALDKFTEVDGEFMSGPTRFVNHS-CEPNMRIFARVGDHAHI--HDLALFAI-KDIPRGEELTFDYVDGVSHE
EAA28243 Nc              VPLQIFRTKD--RGWGVKCPVNIKRGQFVDRYLGEIITSEEADRRRAE---STIARRK----DVYLFALDKFSEVDGEYMSGPTRFINHS-CDPNMAIFARVGDHAHI--HDLALFAI-KDIPKGTELTFDYVNGLTGL
XP_370355 Mg              LPLQIFRTDDG-RGWGVRATVDIKCGQFVDTYIGEVITDSEAVERR------KATRKK----DLYLFDLDKFWVIDGEYRSGPSRFFNHS-CDPNMRIFARVGAHANL--HDLAFFAI-RDISNGEELTFDYVDGQVLP
NP_595186 Sp              LPLEIFKTKE--KGWGVRSLRFAPAGTFITCYLGEVITSAEAAKRD-K----NYD-DDG---ITYLFDLDMF-DVDAQNYGDVSRFFNHS-CSPNIAIYSAVRNHGTI--YDLAFFAI-KDIQPLEELTFDYAGAKDFS
XP_752474 Af              VRLEIFHTGN--RGFGLRSPDWIRAGQFIDCYLGEVITKQEADVRE-E----VVTSQHG---HSYLFELDFF-HVDGQKFGSPTRFMNHS-CNPNCKLFPVTRTYGRL--YDLAFFSL-HNIPPNTELTFDYNPNWEEG
NP_084517 Mm              --EGMKIDLIDGKGRGVIATKQFSRGDFVVEYHGDLIEITDAKKREAL----YAQDPSTGCM-YYFQYLSKTYCVDATQETNLGRLINHS-KCGNCQTKLHDIDGV----PHLILIAS-RDIAAGEELLYDYGDRSKAS
NP_650354 Dm pr-set7      ---------------GVVADRPFKRNEFVVEYVGDLISIGEAAEREKR----YALDENAGCM-YYFKHKSQQYCIDATVDTGLGRLINHS-RAGNLMTKVVLIKQR----PHLVLLAK-DDIEPGEELTYDY-------   SET8
XP_446364 Cg              -KVEIRECEELRKGRGLYATRDIQQGELLFHEKVPMDKLKLIRSGKSCS--MCGD-ITHPY---KHAKISQRVRYESSDSNNIGLYLNHN-CEPN-VRYDINNK------LELKVYAR--KIKKDEELVTTYVNPLHGV
NP_983709 Ag              -KVQVTWSE---KGKGLVARRPLAKGELVFAEDAPLEKSTLVHLSKACG--SCGD-TTHDF---KFAEVSQRVRLHAADTMNSGIYLNHS-CEPN-IYYELEG-------HHINVYAR--KIKSDEELTVSYVNPLHDV
P38890 Sc SET5           -KVEVKFIDD-EHGRGLFAKRDFSKGQIILKENKPLDKLFLISNGKACA--RCGHASLHEL---YYGSISQRERIKLRDASGIGVYINHD-CEPN-YIEQVEEH------EELRLHAR--KIKKGEQIRITYVNPLHGV   SET5
EAK96662 Ca              -NVHIIMTS--KRGKGLYAKRDIAKGDLIWSEEPPLANVNLMKTASACT--YCGDGNLHSL---KYARVSQDVRYKALSS--SAVFLNHN-CASNTVETELNRT------AGLKVIAG--RIKSGEELTTTYVNPSHTV
XP_449432 Cg              EYFDINETSW--GGRACFSKRDLNNGQVVLEVEDSIDYDFRKEVCHYCY--KYNYLSIHNI---ELIQISEDEYNCCRFVSETLVFFNHS-CDPNITKTRIDRK-------MVFTLNR--DVACGEELNIDYSGVLDLP
NP_015160 Sc SET6         PFFQVRQTKW--GGRACFSNGNIPKGTTVLQVSNSISYEFRKEVCHNCF--AYAYLQIPNI---ELIPTCEDEYCCIRFVCESLVFFNHS-CNPNITKYRKGNS-------MLFTMNR--DIKKDEQICIDYSGVLDLP
NP_982445 Ag              ELFEVKLTDY--GGRACFARQSQQRGDTVLKLTQSIAYEYRKEVCHFCF--AYDYMQMESA---ELVAIDEEVYSCARFVTECLVLFNHS-CAPNLAKKRVGRE-------MYFVLTS--DVAAGEQLCIDYKGILDLP   SET6
EAK94622 Ca              PWFIVDETKY--GGRGCFAPKVIPQHTVIHSCQTTLSKPFKKEVCSQCY--TYTFTHQDIN---VLVRIDDNEYLEIKYVIGVLVYFNHS-CSPNIVKTRNNSE-------MVFTTSK--DIEIGEELCISYGNYTDEP
NP_572675 Dm              LPYRVEHSDIY--GRYLVANRQLEAGETLIRAIGMIVRILLLRQHDP-EQ--FLIARMESHTAEQVHEVCGILDVNCFEIGQSAFLLAHD-CTPNTAHTDDPS--S----FEILLRTS-RRVRERERALTLSYAYTLQGT
NP_609464 Dm              NRTAVQWSPVC--GRYLVAKGAIRGHGLLIEAVGMPLRVLLAKEANP-ER--WEVAPMEHHKEELIMQVVGVLEVNAFEARSYTGILAHN-CVPNTSRSIYPSE-G----YKIRLRAM-VDLEEGQPLHHSYTYTLDGT
NP_649084 Dm              QCFEIATNEVL--GRHLRATRDIKIGEQILKVLGMLLRCMHLKDKDP-DA--FKLYNLEDHLEMDILRIAAILDTNTFEVRQGAAMISHD-CVPNMRHRFDDD-------MNIVFLAK-RKIAKGEILSISYTQPLRST
NP_610944 Dm              HPFKIAHNEQL--GRHLVATRTIKPYEIVLKVRGSTVRCLLIGETST-EK--AKFQDLESLEEEEIMKAVGALQINGHEV--TASFTENS-CLPNLAKSFNKN-------GHCILWAP-REIKKNAHLSICYSDAMWGT   SET-Dm
NP_001014717 Dm          PNWTISSSTVA--GRGVFATRDIAAGELIFQVTGTAVRVFHLGKEQR-HL--VMQANAERAYMDQLFRIVGVLNTNAFEAPCLTAIMNHE-CTPNASHYFENGR-------LAVVRAA-RDIPKGGEITTTYTKILWGN
CAA15694 Dm              PSWRVADSPIS--GRGIFATREIAAGEELFRLVGSVVRCFFLDEASR-KL--LMQANMDRYYLDYFYRTICAFNTNAFESRSLAGLLNHQ-CTPNAAHHFENGE-------TIVVCAT-ERIPAGAEITMSYAKLLWST
NP_572539 Dm              PAWEIGVSKIA--GRGVVATRSLKRGEIIFRLIGCVARAINLSKEQR-DL--ILQANLDNNHIEIMNRTVAVLRTNGFDKTTLFGVVNHD-CIPNAYYTFEEKTN------NMIVRAA-VDIPEGFEVTTTYTKLFTGN
NP_081072 Mm              --GGLERFCSAGKGRGLRALRPFHVGDLLFSCPDVECQKEDW-PLHKLEC-QFYS-KYLEFPHSSLVVLFAQVNCNGFTIEDEE-LMNHS-CCPNVIVTYKGT--------LAEVRAV-QEIHPGDEVFTSYIDLLYPT
NP_033892 Mm              --ENVEVFTSEGKGRGLKATKEFWAADVIFAERDRTCQKDAW-LNHKNEC-QYWPPQSQQFSMQYISHIFGVINCNGFTLSDQRGLVNHD-CWPNCTVIFNNG--------KIELRAL-GKISEGEELTVSYIDFLHLS
XP_381344 Fg              APFRVKSHR---KGRGIFSTKSFAPGDVILPFTDAACQAANWTAVHSKEC-AFAGLGTGQEDVQKALTILCKIQTNAFHRYDTD-MANHS-CIPNAMVQFIGR--------RAILRAE-KPIKIDDEIEISYTDYTFPL
EAA36113 Nc              PKTRIGGIPGVDRGRSLFSTERFGAGETIAIIENAECQRAHWKLVHKAEC-VYAGLLQSDETLKQAMEVLCKIQTNAFNRFDAD-MVNHS-CVPNAYIAFEKR--------KAFLKAE-RDLEPGDEILISYIGMACGV
NP_849969 At              RCLGVSNLP--QKGRSLFTARDFRPGEVILSQKGSSCQKSEW-KLHRDEC-VNLILQFPSVDLREIAENFSKFSCNAHSICDSE-IINHS-CSPNAVLVFEEQ--------MAVVRAM-DNISKDSEITISYIETAGST   SET-MYND
XP_755341 Af              --TRGYPSPSPGAGTGIFAATNIPVGGTVMLIQNKTCQAKDWKFVHSFEC-AVKKYSGCALDEEMLCHYGAKLELNSFN-FHSPLLINHS-CDYNSVVGSDGD--------ALYVKAL-RPIRKGEEILVSYIDATNPY
NP_596514 Sp              -APLIASVILPEFGKGTVATDNIPIGKIIIRKRSKGCQKADW-PFHKLEC-HYTQIY----QAELFQKLFCRLAVNAMNLVTSS-RLNHS-CDPNCQIIFDGA--------IVQLVSK-RDIKKDEQLFISYIDIRLPK
XP_365031 Mg              RPLEIRESNIPNAGSGLFTSAPIPVGVEIFRSSSQACQRNAWKTFHKLEC-GWGRRMGTDVDFMTVWELLCIIFTNSMNVRPAEAMINHD-CSPNAHVFFEGS--------QVRVRSL-KAIAAGGEITVSYCDPRLDV
XP_364335 Mg              GTYAVVPVPGK--GLGVVATRHIRAGEILMADDPLLVADVAVASRG-------ELMRLAVERSGGD-AVEDVLNTNTFAVGVEIARINHA-CSPNSFSRFHPSDLT------MDVGAM-RDIMPGEEITISYIPLGLPS
XP_370301 Mg              PSWEVKKVPGK--GLGMVAKRRIPRGETFLVDYAAILASVEFPGRVR-RA--QELLDRAAGQTQGAPVVEDVMRTNTFSLEITISRINHA-CGPNSQVKFDPATLS------QKAFAS-HDIEAGEEITISYAEFGMTY
EAA49663 Mg              RPYVAVPVPGK--GIGLRATQPIRTGHLVTARTPAVMVDERALTGLA-PR--DQLLVPAVGGKDHTDKVHKIYSTNAYRTSIEVSRMNHE-CSPNCAAYFDPMTMS------QRIYAI-RDIMPGEELTVSYIDPVQTR   SET-Mg
XP_362227 Mg              AVYKSVMVPGK--GVGLVAARTLYRGDRIMSSTPAIVIDYGFFETMP-KK--IEIQTAAVDIENYQQKVDKILSTNAFDIDAEISRCNHD-CRPNADYYYDPETLT------QHVHAV-RQINIGEEISLSYLDPLQSQ
XP_364537 Mg              RPFRAQAIPGK--GVGLVAKRPIAKGELVMAETPAILVHKLALEEAT-DV--FSLELAVSKMQGAMGIMDRLF-TNSFQMALNVSKLNHD-CRPNMVFHID-NNLV------HRTHAV-RDIKPGEELTISYVDQMDPA
XP_384827 Fg              RPVEIRDSPGR--GRGLFTTKSVSAGDLLLVEKAKLYHDSDSLSAFQDLV-G--YQKVTV--ECDGAPVVDSFLVEKIISLNLAARINHS-CVGNCRRSFIGD--------IQIVRAA-RDIPAGTELTFPYCPTGDSE
XP_380216 Fg              APVEIRESPGR--GKALFTTKAVSAGELLLCKKAKLYHNSSLSAEFGDLH-D--YQKAMV--ETDGTPVVDSFLVEKIASLNLASRINHS-CVGNCRPSFIGD--------MQIVRAT-KDVPAKTEIFFCYRPPVPFE
EAL91344 Af              GPVAVRPTESR--GRGLFTTEAVKAGDLLFCEKAKLYKNPSTIPAFIDLY-S--YQPVAV--EVDDTPIVDTFLVEQIVALNLASYINHS-CCSNARRSFIGD--------MMIVRAA-QDLAAGTEITFWYQSPLNSD
XP_367445 Mg              NRTRIAKTDNR--GRGLFATVPLCEGDVIFVEKAKMTWNPSLAKRYYDLF-GK-FGDDKQP-VVDGKVAVDTFRVQSIAELNHAAYANHT-CIPNATRAFIGD--------MMIVRAA-RDIPAGAEIFMGYASLAEPF   SET-TPR
XP_366224 Mg              RRTEERPSATH--GRGLFATERIAAGELVFVEKARMLDSPTLARRLCRMH-DYAH-ATGREGVVDGAAVVDVFHAELLRVRNLAAAMNHA-CVASTVRAFVGD--------VFITRAL-RDIEPGEELTQQYVPVRADV
EAA35550 Nc              RNTRVADSGFH--GRGLFATRPLKAGDLVYVEKAQLCDNPSLADTMLKLY-QLPYDLTGAEGMVDGVPVVDVFVVEGIRTKNHSSLMNHS-CVPNTMRSFVGD--------MLICRAT-RDVQEGEELFQQYVPVKTLV
AAF87042 At              GSIEIKKSELS--GRGLFATKNIVAGTLVLVTKARVVSALSTGQGEDSLE-EIALFRPDE--FETCGDWKQSLDTEKLLSILLASFINHS-CIPNARRLHVGD--------YVIVHAS-RDIKTGEEISFAYFDVLSPL
NP_542983 Mm SET7         ERVYVADSLISSAGEGLFSKVAVGPNTVMSFYNGVRITHQEVDSRD------WALNGNTLS-EETVIDVPEPYNHVSKYCASLGHKANHS-FTPNCVYDLFVHPRFG----PIKCIRTLRAVEAEEELTVAYGYDHSPP   SET7/9
```
