# Supplementary material for: Evolution of SET-domain protein families in the unicellular and multicellular Ascomycota fungi
Source: BMC Evol Biol. 2008 Jul 1;8:190. doi: 10.1186/1471-2148-8-190 (PMC2474616; doi:10.1186/1471-2148-8-190)
Supplement: Additional file 9 — The maximum parsimony tree reconstructed from the 113 representative SET-domain sequences. [file 1471-2148-8-190-S9.pdf]

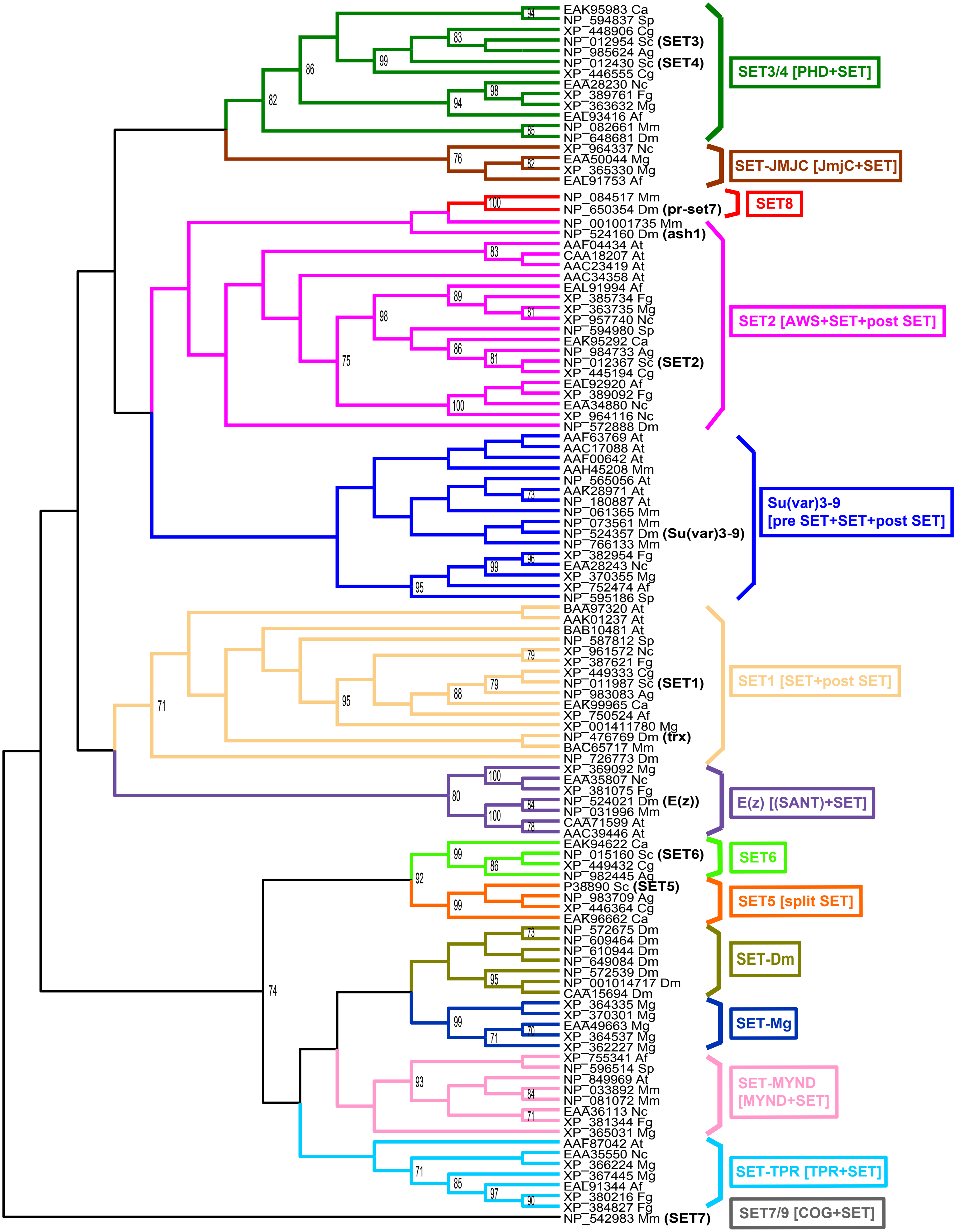

Maximum parsimony phylogeny of 113 representative SET-domain sequences. The consensus phylogeny from bootstrap analysis with 500 pseudoreplicates is shown with bootstrap values greater than 70%.
